# Prediction criterion and numerical validation for the interaction between hydraulic fractures and bedding planes

**DOI:** 10.1371/journal.pone.0294993

**Published:** 2023-12-21

**Authors:** Xiaoxi Men, Zhihui Han

**Affiliations:** 1 College of Civil Engineering, Dalian JiaoTong University, Dalian, China; 2 ACRE Coking And Refractory Engineering Consulting Corporation, MCC, Dalian, China; Xi’an University of Science and Technology, CHINA

## Abstract

Shale is a kind of sedimentary rock with an obvious bedding structure. The effect of the bedding plane on hydraulic fracture initiation, propagation, and complex fracture network formation is remarkable and a major problem in hydraulic fracturing and shale oil and gas development. In this study, a criterion is established to predict the evolution behavior of hydraulic fractures (HF) under different confining pressure differences and intersection angles. This criterion is intended to predict the types of interaction between HFs and bedding planes (BPs): penetrating, slipping, or dilating. The dependence of crossing on the intersection angle and the principal stress difference is quantitatively presented using the criterion. Meanwhile, 20 simulations with principal stress differences of 2, 4, 6, and 8 MPa and intersection angles of 30°, 45°, 60°, 75°, and 90° were simulated using the RFPA^2D^-Flow code. Simulation results exhibit good agreement with the criterion results for a wide range of angles. The investigation showed that HFs tend to penetrate BPs under high confining pressure differences and intersection angles and open BPs under low confining pressure differences and intersection angles. In addition to the above two forms, HFs slip due to shear. The criterion can provide relevant reference about the formation of complex fracture networks in shale layers.

## Introduction

Shale oil and gas are a kind of clean, efficient, and environmentally energy. As one of the unconventional oil and gas resources, shale is receiving increasing attention around the world because it is one of the most realistic alternative resource replacement for conventional oil and gas [1–5]. As a kind of sedimentary rock, shale is characterized by significant bedding and its formations are known to have a fine layered structure [6]. The existence of bedding planes (BP) affects the stress distribution, thus affecting the fracture propagation trajectory [7]. Many studies have shown that the existence of bedding promotes the formation of fracture networks in shale reservoirs [8–13], thus promoting shale reservoir permeability and oil and gas production [14–16]. Since 1947, the year of the first experimental hydraulic fracturing treatment in the United States in the Hugoton gas field in Grant County, Kansas [17], hydraulic fracturing treatments have become an essential part in gas and oilfield development [18–20]. Therefore, the investigation of the interaction between hydraulic fractures (HFs) and BPs is of great significance for the complex network formation [13], the reconstruction of shale reservoir, and even the exploitation of shale oil and gas.

**Fig 1:**
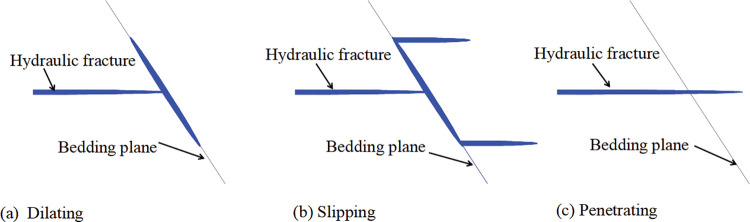
Three main modes of interaction between HF and BP.

In recent years, some scholars studied hydraulic fracturing problems, especially the HF evolution in discontinuous shale reservoirs and have drawn some valuable conclusions. Weak discontinuities, such as natural fracture(NF) and moderate development bedding, are beneficial to oil-gas recovery because the interaction between HFs and discontinuous is an important factor of the hydraulic fracture network’s complexity [12, 21–23]. The interaction between HFs and discontinuities(i.e. BPs in this study) can be observed in three main modes [6, 7, 11, 19, 24, 25]: 1. HF opens the BP and propagates along the BP (dilate); 2. HF is captured by the BP, and then the BP shear slips (slip); 3. HF passes through the BP (penetrate), as shown in Fig 1. Through numerous theoretical analyses, physical experiments, and numerical simulations, several criteria have been proposed to predict the occurrence of these three interaction modes, such as the Blanton [26], Warpinski [27], Renshaw [28], Zhou [25], Gu [19], Cheng [29], Sarmadivaleh [30], and Gong [31] criteria. Some of these criteria depend on the output parameters in the construction process, some only consider whether HF penetrate the discontinuities, some are extremely complex to use directly, and most studies focus on the interaction between HF and NF.

This paper proposes a usable criterion of the interaction between HF and BP on the basis of fracture mechanics to predict the dilating, slipping, or penetrating behavior of HF after encountering BP using some parameters as input. The HF evolution of the shale layer is simulated at different principal stress differences and intersection angles to verify the criterion.

### Interaction criterion between HF and BP

The interaction between a HF and a BP is a complex process. On the basis of the experiment results and theoretical analysis, three modes may form during the propagation of the HF toward the BP and beyond. First, when the fracture tip reaches the interface, the BP will dilate (Fig 1A) and the HF will propagate along the BP if the pressure in the HF exceeds the sum of the normal stress acting on the BP and the tensile strength of the bedding. On the contrary, if the pressure in the HF is less than the sum of the normal stress acting on the BP and the tensile strength of the bedding, two possible outcomes form the interaction between the HF and the BP, namely, slipping (Fig 1B) and penetration (Fig 1C).

**Fig 2:**
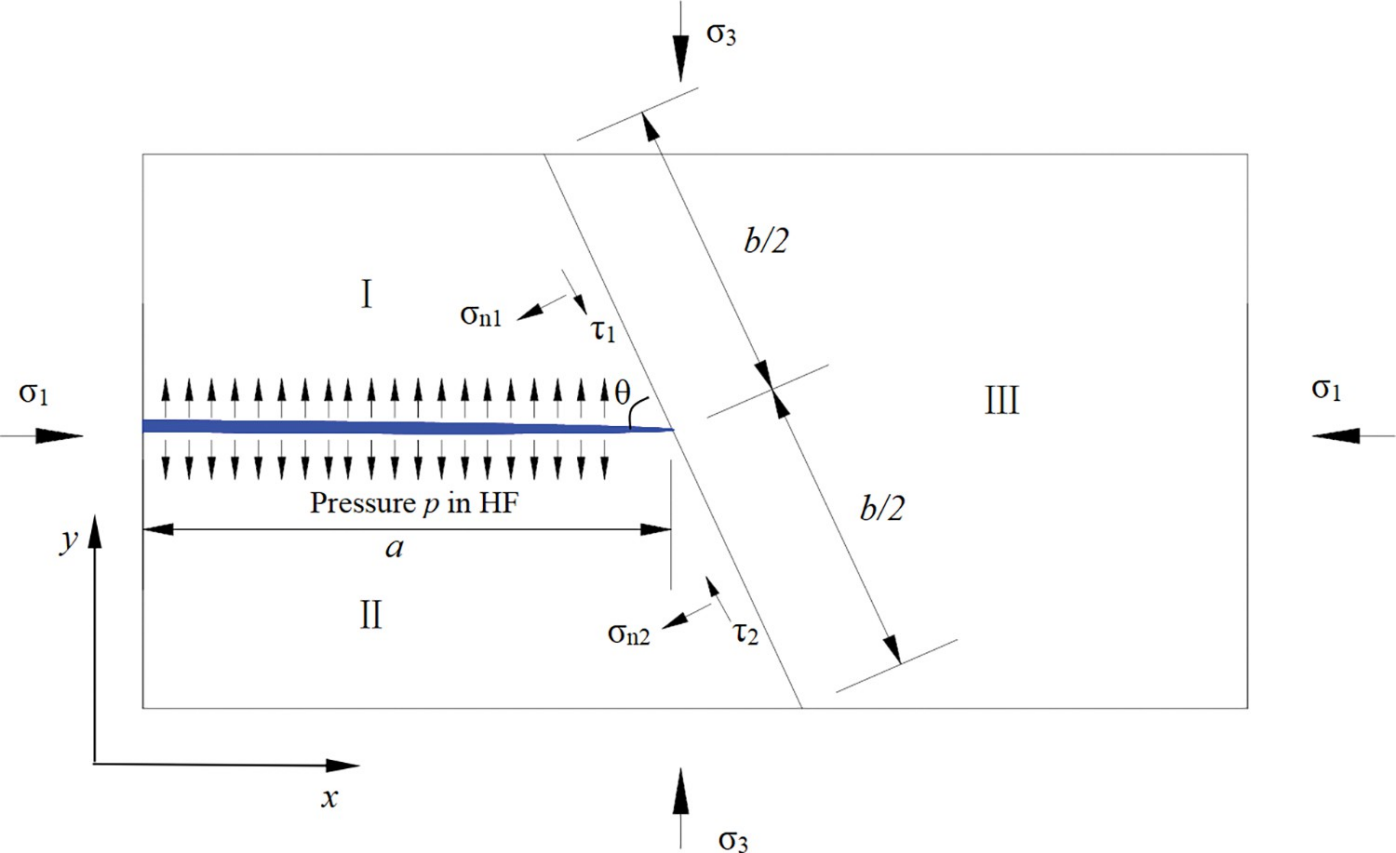
Schematic of HF encountering BP.

To derive the prediction criterion, a rectangle model is set up. As shown in Fig 2, the minimum principal stress (*σ*_3_) is applied in the vertical direction, and the maximum principal stress (*σ*_1_) is applied in the horizontal direction. The BP approaches HF at an arbitrary angle (*θ*). The half-length of the HF is denoted as *a*, and length of the BP is *b*. The model is divided into three regions by HF and BP, and the stress on the BP can be calculated by the equilibrium equations of Regions Ⅰ and Ⅱ. In this derivation, the stress in the HF and the BP is uniform to simplify the calculation.

The model is in an equilibrium state, and the boundary stress distribution of Regions Ⅰ and Ⅱ is shown in Fig 2. The equilibrium equations of Regions Ⅰ and Ⅱ are as follows.

In Region Ⅰ,

$$\begin{array}{l} \sum F(x)=0\\ \frac{b}{2}\sigma_{1}\sin\theta =\sin\theta \int_{0}^{b/2}\sigma_{n1}ds-\cos\theta \int_{0}^{b/2}\tau_{1}ds\\ \sum F(y)=0\\ \int_{0}^{a}p(x)dx-\cos\theta \int_{0}^{b/2}\sigma_{n1}ds-\sin\theta \int_{0}^{b/2}\tau_{1}ds=\sigma_{3}(a-\frac{b}{2}\cos\theta ) \end{array},$$ 1

and in Region Ⅱ,

$$\begin{array}{l} \sum F(x)=0\\ \frac{b}{2}\sigma_{1}\sin\theta =\sin\theta \int_{0}^{b/2}\sigma_{n2}ds+\cos\theta \int_{0}^{b/2}\tau_{2}ds\\ \sum F(y)=0\\ \int_{0}^{a}p(x)dx-\cos\theta \int_{0}^{b/2}\sigma_{n2}ds-\sin\theta \int_{0}^{b/2}\tau_{2}ds=\sigma_{3}(a+\frac{b}{2}\cos\theta ) \end{array}.$$ 2

The stress in the HF and the BP is considered uniform. Therefore,

$$\begin{array}{l} \sigma_{n1}=\frac{\int_{0}^{b/2}\sigma_{n1}ds}{b/2}\;\sigma_{n2}=\frac{\int_{0}^{b/2}\sigma_{n2}ds}{b/2}\\ \tau_{1}=\frac{\int_{0}^{b/2}\tau_{1}ds}{b/2}\;\tau_{2}=\frac{\int_{0}^{b/2}\tau_{2}ds}{b/2} \end{array}.$$ 3

Then, solutions can be obtained by solving the above equilibrium Eqs (1 and 2):

$$\begin{array}{l} \sigma_{n1}=\frac{\sigma_{1}+\sigma_{3}}{2}‐\frac{\sigma_{1}‐\sigma_{3}}{2}\cos2\theta +\frac{2a(p-\sigma_{3})}{b}\sin\theta \\ \tau_{1}=\frac{\sigma_{3}‐\sigma_{1}}{2}\sin2\theta +\frac{2a(p-\sigma_{3})}{b}\sin\theta \end{array},$$ 4

$$\begin{array}{l} \sigma_{n2}=\frac{\sigma_{1}+\sigma_{3}}{2}‐\frac{\sigma_{1}‐\sigma_{3}}{2}\cos2\theta -\frac{2a(p‐\sigma_{3})}{b}\cos\theta \\ \tau_{2}=\frac{\sigma_{1}-\sigma_{3}}{2}\sin2\theta +\frac{2a(p-\sigma_{3})}{b}\sin\theta \end{array}.$$ 5

When the fracture tip reaches the interface, the BP will dilate if the pressure in the HF exceeds the sum of the normal stress acting on the BP and the tensile strength of the bedding. The dilation condition is *p*>*σ*_*n*1_+*T*_*O*_ or *p*>*σ*_*n*2_+*T*_*O*_. Given that *σ*_*n*1_>*σ*_*n*2_, the dilation condition is simplified to

$$p>\sigma_{n2}+T_{O},$$ 6

where *T*_*O*_ is the tensile strength of the bedding. Then, Eq 5 is substituted into Formula 6 to obtain the following:

$$p‐\sigma_{3}>\frac{b[(\sigma_{1}-\sigma_{3})(1-\cos2\theta )+2T_{O}]}{2(b+2a\cos\theta )}.$$ 7

When the fracture tip reaches the interface, shear slip may occur on the BP if the pressure in the HF is less than the sum of the normal stress acting on the BP and the tensile strength of the bedding. The slip conditions are *p*<*σ*_*n*1_+*T*_*O*_ and *p*<*σ*_*n*2_+*T*_*O*_, *τ*_1_>*S*_*O*_+*f*(*σ*_*n*1_−*p*), or *τ*_2_>*S*_*O*_+*f*(*σ*_*n*2_−*p*). Given that *σ*_*n*1_>*σ*_*n*2_ and *τ*_2_>*τ*_1_, the slip condition is simplified to

$$p<\sigma_{n2}+T_{O},$$ 8

$$\tau_{2}>S_{O}+f(\sigma_{n2}-p),$$ 9

where *S*_*O*_ is the shear strength of the bedding, and *f* is the friction coefficient of the bedding. Then, Eq 5 is substituted into Formulas 8 and 9 to obtain the following:

$$p‐\sigma_{3}<\frac{b[(\sigma_{1}-\sigma_{3})(1-\cos2\theta )+2T_{O}]}{2(b+2a\cos\theta )},$$ 10

$$\mathrm{And}\;p‐\sigma_{3}>\frac{2bS_{O}+b(\sigma_{1}-\sigma_{3})[f(1-\cos2\theta )-\sin2\theta ]}{4a(\sin\theta +f\cos\theta )+2bf}.$$ 11

When the fracture tip reaches the interface, the HF may cross the BP if the pressure in the HF is less than the sum of the normal stress acting on the BP and the tensile strength of the bedding and shear slip does not occur. The penetration conditions are *p*<*σ*_*n*1_+*T*_*O*_ and *p*<*σ*_*n*2_+*T*_*O*_, *τ*_1_<*S*_*O*_+*f*(*σ*_*n*1_−*p*) and *τ*_2_<*S*_*O*_+*f*(*σ*_*n*2_−*p*). Given that *σ*_*n*1_>*σ*_*n*2_ and *τ*_2_>*τ*_1_, the penetration condition is simplified to Formula 8 and

$$\tau_{2}<S_{O}+f(\sigma_{n2}-p).$$ 12

By substituting Eq 5 into Formulas 8 and 12, Formula 10 and

$$p‐\sigma_{3}<\frac{2bS_{O}+b(\sigma_{1}-\sigma_{3})[f(1-\cos2\theta )-\sin2\theta ]}{4a(\sin\theta +f\cos\theta )+2bf}$$ 13

be obtained.

In fracture mechanics, the propagation of HF is controlled by the fracture toughness (*K*_*IC*_)_._ The half length of the HF is *a*, and stress intensity factor $K_{I}=\sigma \sqrt{\pi a}$. When the HF approaches the BP,

$$p‐\sigma_{3}=\frac{K_{IC}}{\sqrt{\pi a}}$$ 14

is always satisfied.

By substituting Eq 14 into Formulas 7, 10, 11, and 13, the interaction criterion can be obtained.

Dilation criterion

$$\sigma_{1}-\sigma_{3}<\frac{2\frac{K_{IC}}{\sqrt{\pi a}}(b+2a\cos\theta )-2bT_{O}}{b(1-\cos2\theta )}$$ 15

Slip criterion

$$\sigma_{1}-\sigma_{3}>\frac{2\frac{K_{IC}}{\sqrt{\pi a}}(b+2a\cos\theta )-2bT_{O}}{b(1-\cos2\theta )}$$ 16

$$\sigma_{1}-\sigma_{3}<\frac{4\frac{K_{IC}}{\sqrt{\pi a}}a(\sin\theta +f\cos\theta )+2bf\frac{K_{IC}}{\sqrt{\pi a}}-2bS_{O}}{b[f(1-\cos2\theta )-\sin2\theta ]}$$ 17

Penetration criterion

$$\sigma_{1}-\sigma_{3}>\frac{2\frac{K_{IC}}{\sqrt{\pi a}}(b+2a\cos\theta )-2bT_{O}}{b(1-\cos2\theta )}$$ 18

$$\sigma_{1}-\sigma_{3}>\frac{4\frac{K_{IC}}{\sqrt{\pi a}}a(\sin\theta +f\cos\theta )+2bf\frac{K_{IC}}{\sqrt{\pi a}}-2bS_{O}}{b[f(1-\cos2\theta )-\sin2\theta ]}$$ 19

The criterion is the function of the principal stress difference and the intersection angle. Parameters *a*,*b*,*To*,*f*, and *K*_*IC*_ in the criterion can be input according to the actual situation.

## Numerical simulation of interaction between HF and BP

### Model set

**Fig 3:**
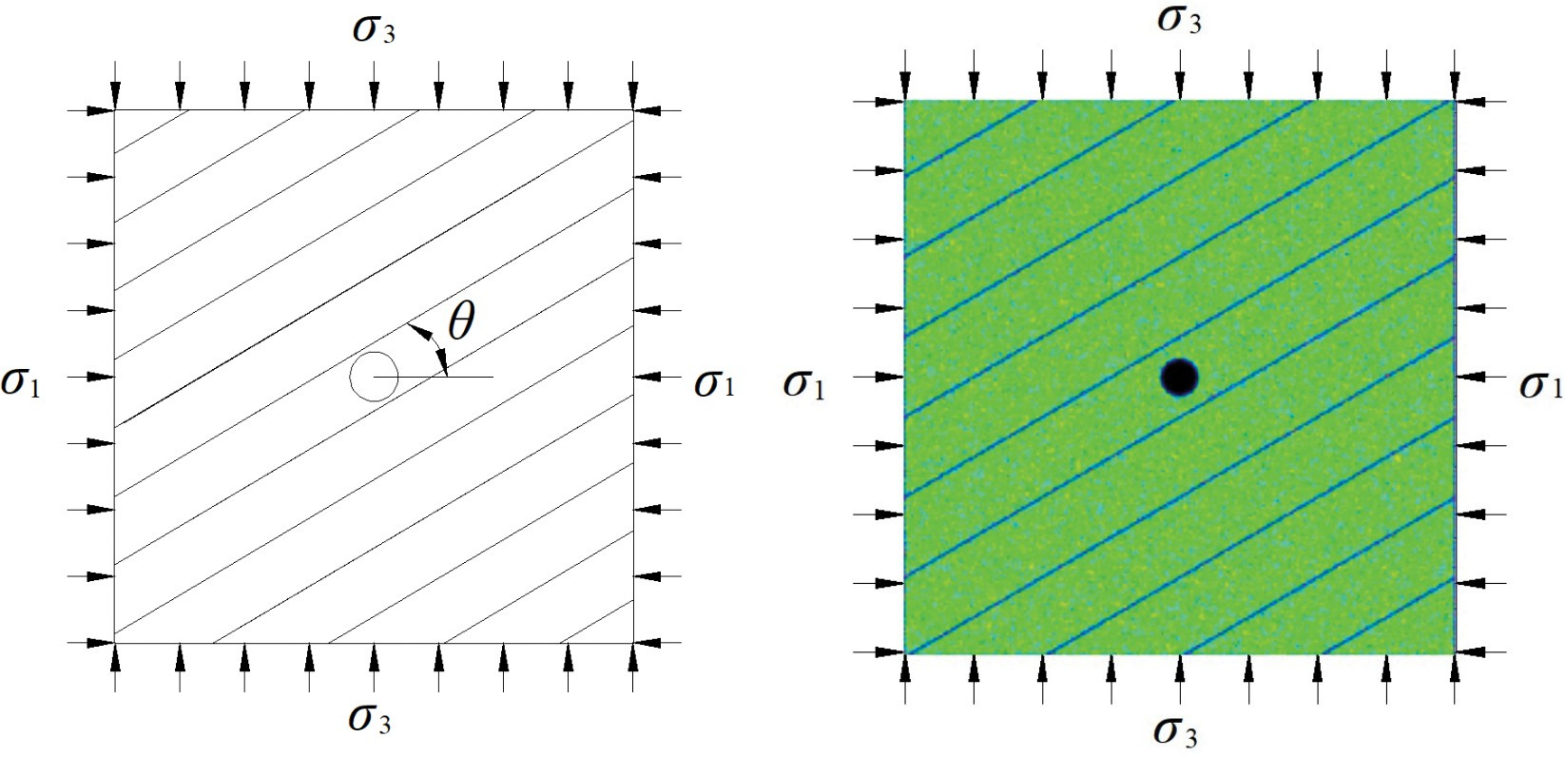
Schematic of the model set and the RFPA model.

The numerical simulation of the interaction between the HF and the BP can be divided into three parts. The first part refers to the experiment in [32] for verifying the reliability of the RFPA-Flow code [9, 10, 33–35]. The second part refers to the verification of the above prediction criterion. The third part refers to the investigation on the influence of the absolute value of principal stress on the interaction. As shown in Fig 3, the dimension of the model is 300 mm × 300 mm, which is divided to form a 300 × 300 mesh. The spacing of the parallel bedding is 37.5 mm, and the thickness of the bedding is 1 mm (single element). The wellbore is located in the center of the model, with a radius of 10 mm. In the first part, the intersection angle (*θ*) is 90° and the principal stress difference is 0.5 MPa, which are according to the experiment. In the second part, the intersection angle is 30°, 45°, 60°, 75°, and 90° at principal stress differences of 2(*σ*_1_ = 4MPa, *σ*_3_ = 2MPa), 4(*σ*_1_ = 6MPa, *σ*_3_ = 2MPa), 6(*σ*_1_ = 8MPa, *σ*_3_ = 2MPa), and 8 MPa(*σ*_1_ = 10MPa, *σ*_3_ = 2MPa). In the third part, the intersection angle and the principal stress difference are same (*θ* = 30°, △*σ* = 2MPa,) but the maximum and the minimum principal stress are different (*σ*_1_ = 4MPa, *σ*_3_ = 2MPa; *σ*_1_ = 6MPa, *σ*_3_ = 4MPa; *σ*_1_ = 8MPa, *σ*_3_ = 6MPa; *σ*_1_ = 10MPa, *σ*_3_ = 8MPa; *σ*_1_ = 12MPa, *σ*_3_ = 10MPa; *σ*_1_ = 14MPa, *σ*_3_ = 12MPa; *σ*_1_ = 16MPa, *σ*_3_ = 14MPa; *σ*_1_ = 18MPa, *σ*_3_ = 16MPa). The HFs involved in this study initiated and propagated in the direction of the maximum principal stress, so the intersection angle (*θ*) is the angle between the BP and the direction of the maximum principal stress. A plane–strain component is employed for calculation.

Rock mass is a highly heterogeneous material containing geological discontinuities such as faults, bedding planes, joints and microcracks [6]. Therefore, the consideration of heterogeneity is particularly important in the numerical simulation of rock. The mesoscopic elements in RFPA models are assumed to be isotropic and homogeneous, and their mechanical properties (*e*.*g*., Young’s modulus, Compressive strength, among others) are also assumed to be linear. The mesoscopic elements are statistically distributed (*e*.*g*., normal, Poisson, and Weibull distributions) to describe the mechanical properties of the nonlinear macro models. In the statistical distribution density of the mechanical parameters of the mesoscopic elements in RFPA codes, a heterogeneity index *m* is used to describe the heterogeneity of solid materials. A high *m* value indicates the presence of highly homogeneous materials whereas a small *m* value denotes the existence of highly inhomogeneous materials. The meso-mechanical parameter values which are input in modeling should be obtained through the simulations of mechanical property experiments by RFPA^2D-^Basic. During this period, the suitable mesoscopic values would be got when macro mechanical properties of the simulations are close to the experimental values through multiple adjustments of the mesoscopic mechanical parameters.

### Numerical simulation of layered shale fracturing experiment

According to the experiment by [32], the value of *σ*_1_ (corresponding to *σ*_*V*_ in the experiment) is 20 MPa and that of *σ*_3_ (corresponding to *σ*_*H*_ in the experiment) is 19.5 MPa. In the experiment, the direction of perforation is normal to the bedding planes. The vertical in situ stress (*σ*_*V*_) is loaded normal to the bedding planes and the greatest horizontal in situ stress (*σ*_*H*_) is loaded along the beddings. The list of rock and bedding parameters used in the simulations is presented in Table 1. The simulation conditions are summarized in Table 2. As shown in Fig 5, the HF initiated and propagated in the vertical direction due to the influence of the in-situ stress. Therefore, the principal stress difference is 0.5 MPa, and the intersection angle θ is 90°.

**Table 1:** Physicomechanical parameters of the rock and bedding in the simulations.

Parameters	Unit	Rock matrix	Bedding
**Heterogeneity index (*m*)**		3	3
**Young’s modulus (*E*)**	GPa	30(meso value is 37)	3(meso value is 3.7)
**Poisson ratio (ν)**		0.34(m = 100)	0.34(m = 100)
**Friction angle (φ)**	°	37(m = 100)	37(m = 100)
**Compressive strength (*f***_***c***_ **)**	MPa	120 (meso value is 355)	40(meso value is 120)
**Permeability coefficient (*K*)**	md^-1^	0.000864(m = 100)	0.00864(m = 100)

**Table 2:** Simulation conditions.

Model setting		Variables	Values
**Model size**	300 mm × 300 mm	θ	90°
**Mesh**	300 × 300		
**Bedding distance**	37.5 mm		
**Bedding thickness**	1 mm	△σ	0.5 MPa
**Wellbore radius**	10 mm		
**Boundary conditions**	Bottom fixed; Confining pressure; Water pressure load in the wellbore		

Acoustic emission (AE) monitoring and the specimen cut were used in the hydraulic fracturing experiment to analyze the results in detail. The AE energy is low when the HF propagates along weak beddings; thus, the local complex sub-fractures are difficult to capture by AE monitoring [32]. RFPA simulation solves this problem well because it can simulate the failure process of heterogeneous and permeable geo-materials and capture the AE events throughout the entire failure process, and it even determines whether the AE event belongs to tensile or compressive failure.

**Fig 4:**
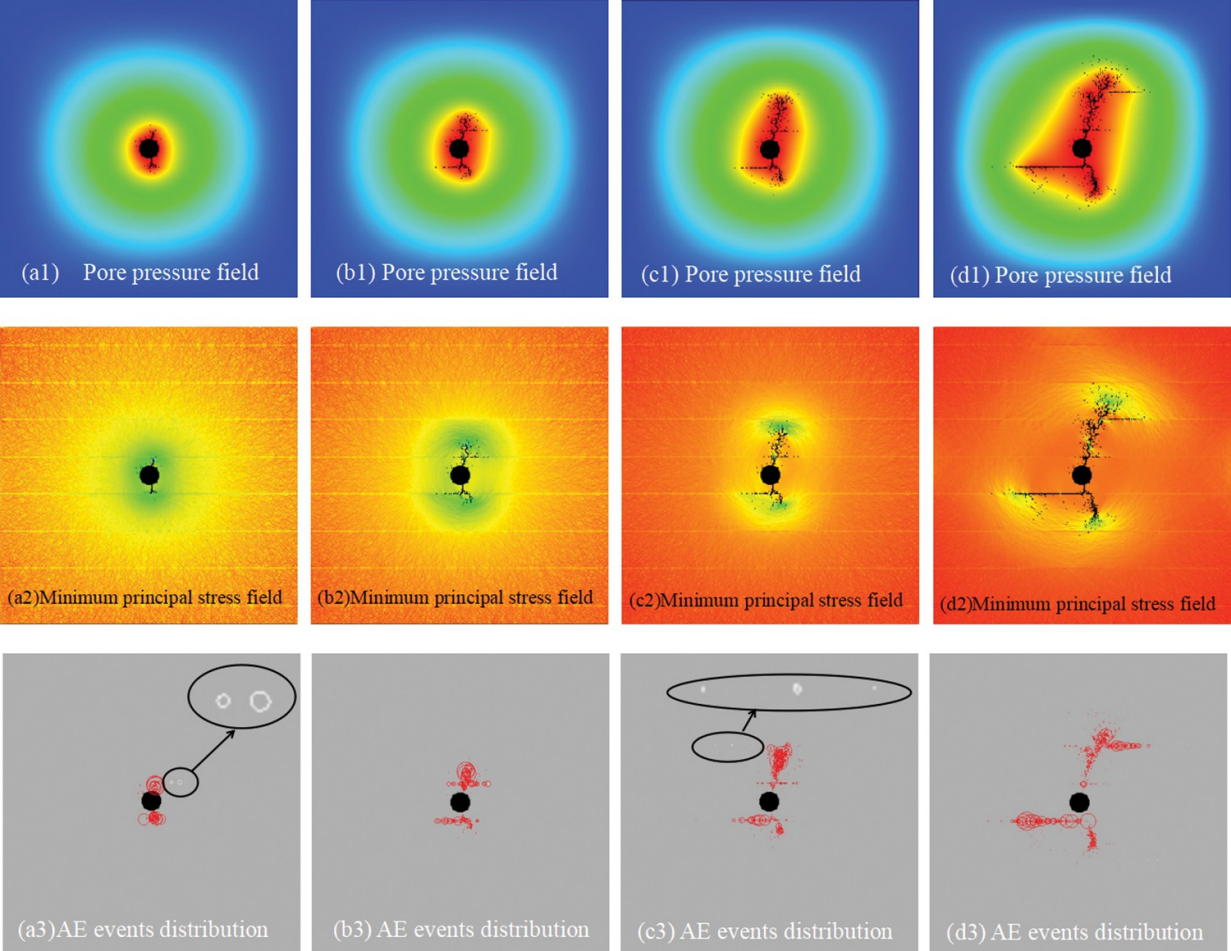
Spatio-temporal evolution of hydraulic fractures of the RFPA model at different injection pressures expressed by pore pressure field, minimum principal stress field and AE events distribution: (a) 31 MPa, (b) 33.7 MPa, (c) 34.5 MPa, and (d) 34.7 MPa, S1 Fig.

The spatio-temporal evolution of HFs is expressed by the pore pressure field, the minimum principal stress field, and the AE events distribution in Fig 4. As shown in the figure, the HFs initiated at the top and bottom of the wellbore and propagated at an angle of approximately 20° with the greatest in situ stress, mainly because of the low confining pressure difference. Then, the HFs approached the BP at an intersection angle of 90° for a period of growth; the top HF penetrated one BP and then the next BP shear slip, while the bottom one slipped directly. Thus, slipping was mainly caused by tensile-shear failure induced by the presence of BPs, which is weaker than rock the matrix. The HF at the bottom had a slow propagation speed than that at the bottom due to shear slip, so the fracture was shorter. Furthermore, the HFs that extended at low speed communicated with the weak beddings by slipping and penetration, thus producing a complex fracture network. The green region around the fracture tip in the minimum principal stress pictures show that the HFs propagation is due to the tensile stress. However, the white circle in the BP in the AE events distributions (a3 and c3 in Fig 4), which indicates that the compression failure shows that tensile failure (red circle in AE events distributions) is the main but not the only form of failure in hydraulic fracturing in the shale layer.

**Fig 5:**
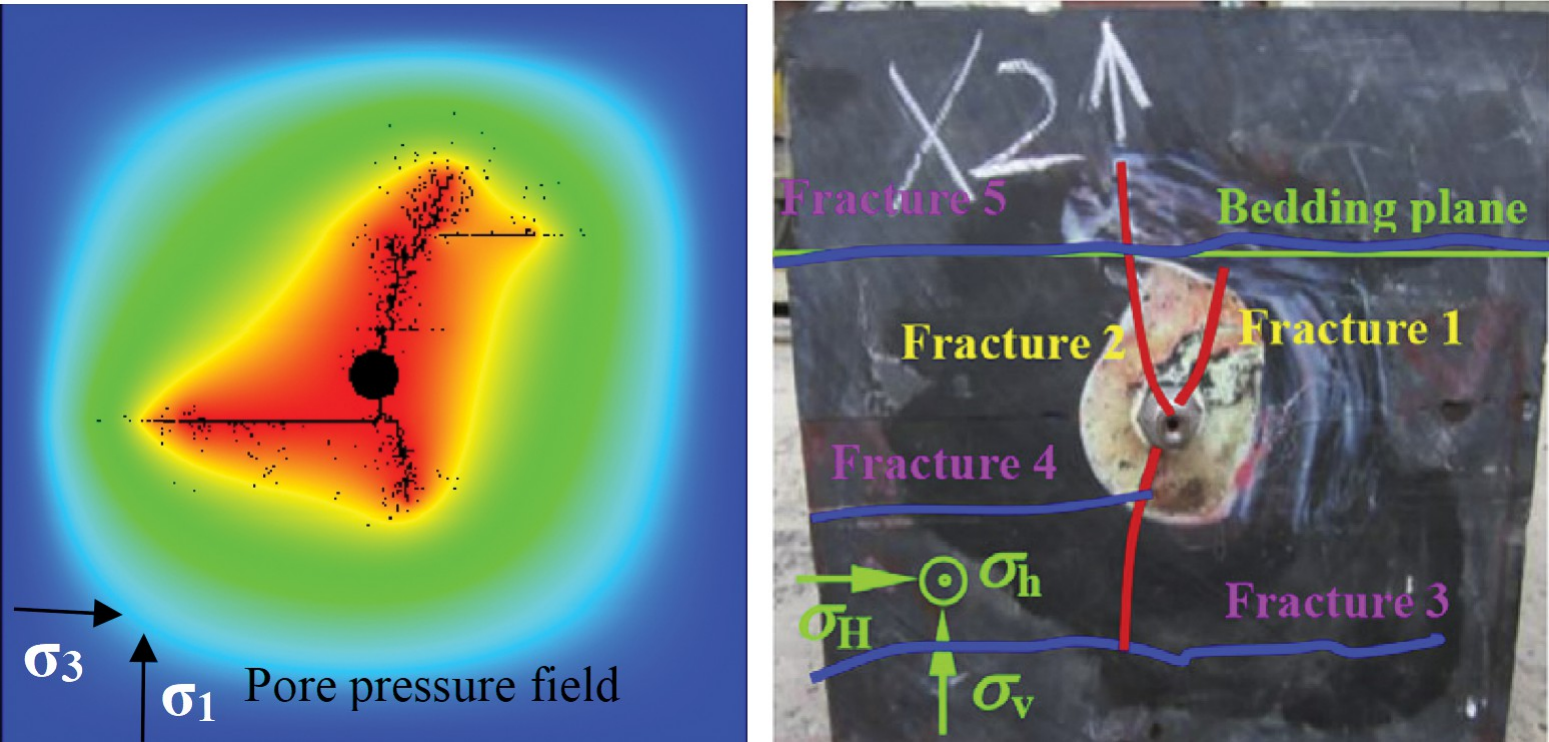
Comparsion of numerical and experimental result [32].

In the simulation, plane strain calculation was used to reduce the influence of the two-dimensional model. The intersection between the HF and the BP, such as penetration and the slip in the simulation during the HF evolution, is basically consistent with the experiment (Fig 5). The result also verifies the reliability of the RFPA code.

### Numerical simulations for verifying the prediction criterion

**Fig 6:**
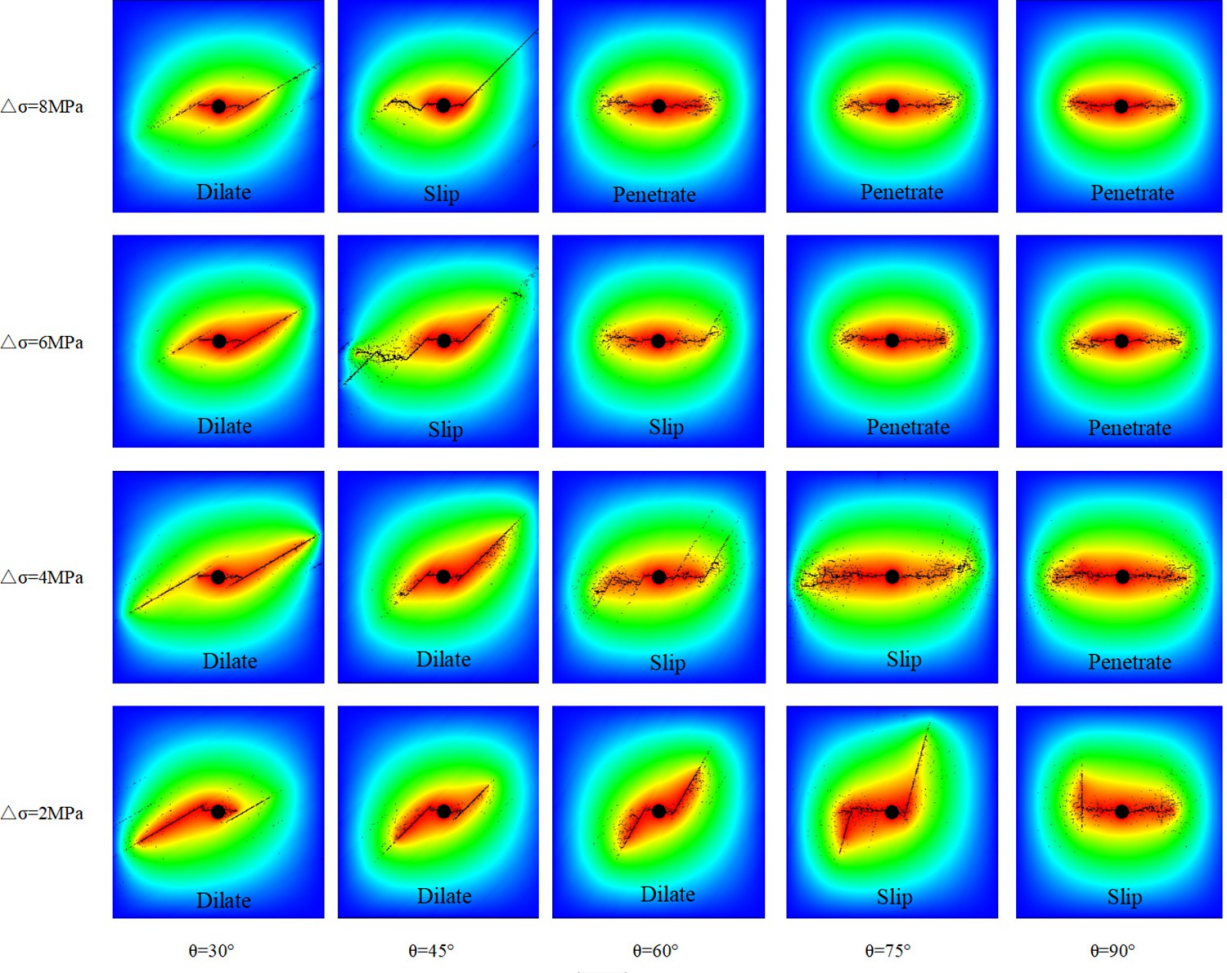
Pore pressure field of different △σ (σ_1_-σ_3_) models with different θ S2 Fig.

In this part, twenty simulations with principal stress differences of 2, 4, 6, and 8 MPa and intersection angles of 30°, 45°, 60°, 75°, and 90° were performed using the RFPA^2D^-Flow code. The list of rock and bedding parameters used in the simulations is presented in Table 3. The simulation conditions are summarized in Table 4. The spatio-temporal evolutions of the HFs of 20 models are shown in the pore pressure field in Fig 6.

**Table 3:** Physicomechanical parameters of the rock and bedding in the simulations.

Parameters	Unit	Rock matrix	Bedding
**Heterogeneity index (*m*)**		3	3
**Young’s modulus (*E*)**	GPa	30(meso value is 37)	3(meso value is 3.7)
**Poisson ratio (ν)**		0.25(m = 100)	0.25(m = 100)
**Friction angle (φ)**	°	37(m = 100)	37(m = 100)
**Compressive strength (*f***_***c***_ **)**	MPa	80 (meso value is 250)	20 (meso value is 65)
**Permeability coefficient (*K*)**	md^-1^	0.000864(m = 100)	0.00864(m = 100)

**Table 4:** Simulation conditions.

Model setting		Variables	Values
**Model size**	300 mm × 300 mm	θ	30°, 45°, 60°, 75°, 90°
**Mesh**	300 × 300		
**Bedding distance**	37.5 mm		
**Bedding thickness**	1 mm	△σ	2, 4, 6,and 8 MPa
**Wellbore radius**	10 mm		
**Boundary conditions**	Bottom fixed; Confining pressure; Water pressure load in the wellbore		

As shown in Fig 6, the HF initiated and propagated with the greatest in situ stress at the beginning. When θ was 30° (△*σ* = 2, 4, 6, 8 MPa), 45°(△*σ* = 2, 4 MPa), and 60° (△*σ* = 2 MPa), the HF dilated the BP and formed a simplex bi-wing fracture as the HFs approached the weak BP. When θ was 45° (△*σ* = 6, 8 MPa), 60° (△*σ* = 4, 6 MPa), 75° (△*σ* = 2 MPa), and 90° (△*σ* = 2 MPa), the BP shear slipped and formed a jagged fracture with a tortuous appearance. In this situation, a complex fracture network was formed by the frequent deflection of HFs and the penetration of the bedding. When θ was 60° (△*σ* = 8 MPa), 75° (△*σ* = 6, 8 MPa), and 90° (△*σ* = 4, 6, 8 MPa), the HF penetrated the BPs and formed a horizontal HF, which propagated in the maximum principal stress direction. Overall, the smaller the intersection angle and the principal stress difference are, the easier the BP dilation by the HF is; the bigger the intersection angle and the greater the principal stress difference are, the easier the BP penetration by the HF will be. In addition, the weaker the bedding is, the easier the BP dilation by the HF will be. In general, the hydraulic fracture evolution was mainly controlled by the principal stress difference, the intersection angle, and the strength of the BP, which explains why these parameters are considered in the criterion.

**Fig 7:**
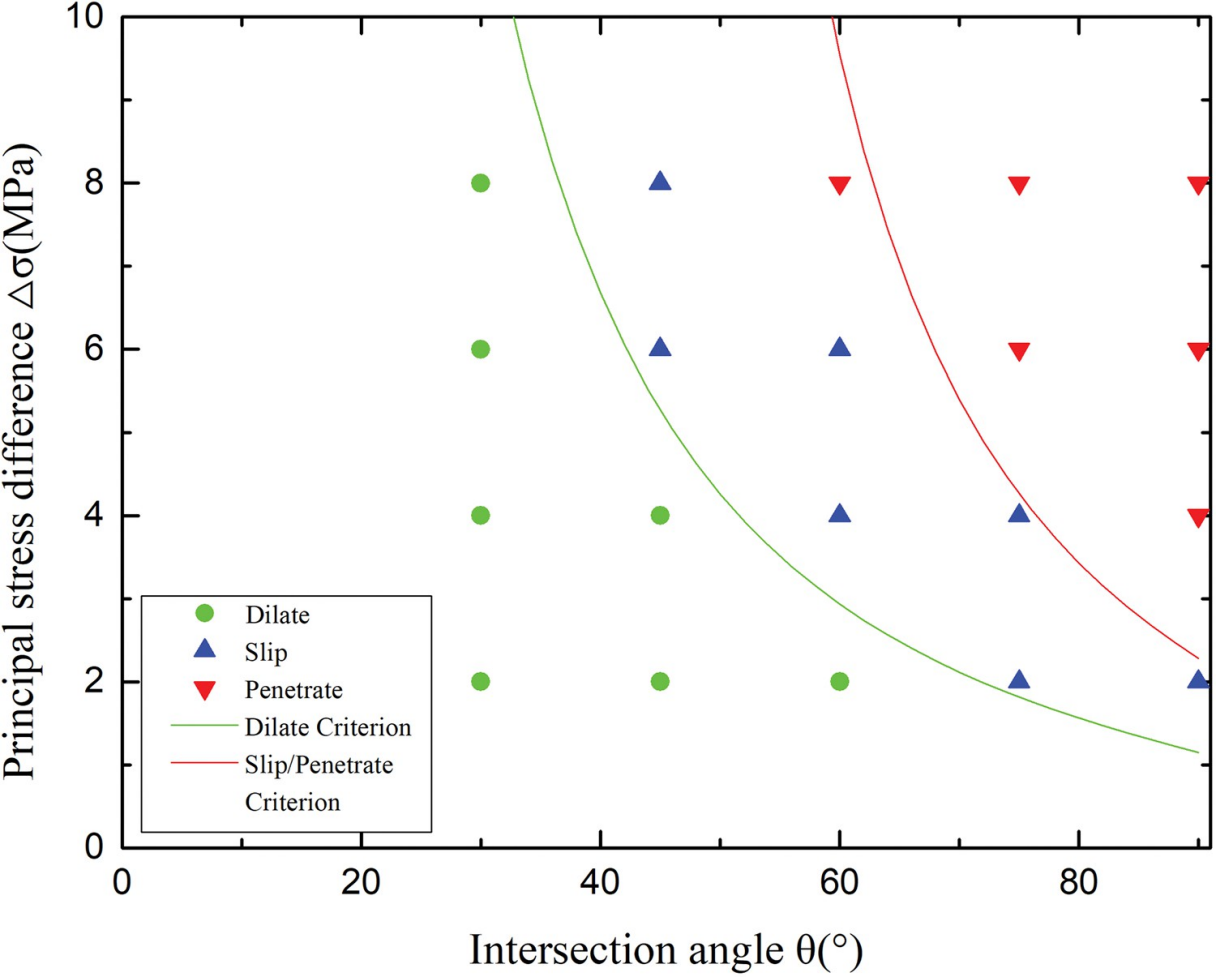
Comparison of criterion and numerical results S1 File.

Fig 7 presents a comparison of the simulation results and the prediction criterion. According to the numerical model, the parameters in the criterion are taken as $\frac{b}{a}=3$ (based on the simulation results in Fig 6, *a* = 0.04m and *b* = 0.12m), $\frac{K_{IC}}{\sqrt{\pi a}}=3.15$ (*K*_*IC*_ = 0.313+0.027*E* which established by Whittaker in 1992 [36]), *T*_*O*_ = 2 (rock’s compressive-tensile strength ratio is taken as 10), *S*_*O*_ = 2.5 (the shear strength is taken as 12.5% of the compressive strength), and friction coefficient *f =* 1.21 due to the thickness of the BP [25]. As shown in the figure, the dilation criterion and slip/penetration criterion curves were obtained after substitution. The simulations are consistent with the criterion. In Fig 7, the simulations and the criterion indicate that the HF would dilate the BP under low principal stress difference and intersection angle and penetrate the BP under high principal stress difference and intersection angle. The BP would shear slip under appropriate intersection angle and principal stress difference, which is conducive to the formation of a complex fracture network.

### Numerical simulations for the influence of absolute value of principal stress on interaction

**Fig 8:**
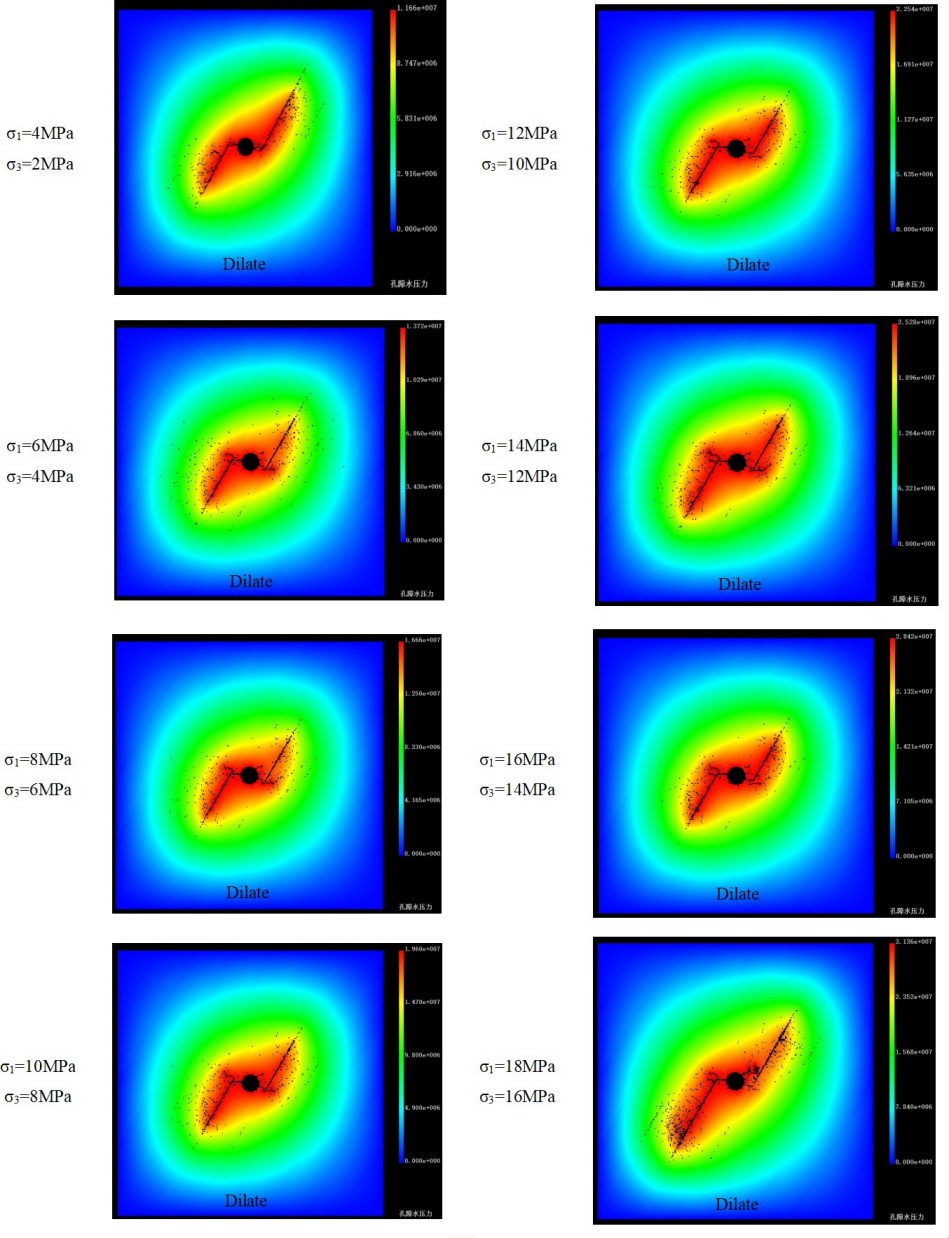
Pore pressure fields of models with different σ_1_ and σ_3_ but same θ (60°) and △σ (2 MPa) S3 Fig.

Considering the absolute value of principal stress would affect the stress state of the specimen, eight simulations with different maximum and minimum principal stress but same principal stress difference and intersection angle were performed using the RFPA^2D^-Flow code. In this part, take the principal stress difference of 2 MPa and intersection angle of 60°as an example to study the influence of the absolute value of principal stress on interaction. The list of rock and bedding parameters used in the simulations and the simulation conditions are the same as Section 3.3. The spatio-temporal evolutions of the HFs of 8 models are shown in the pore pressure field in Fig 8.

As shown in Fig 8, as the maximum principal stress and the minimum principal stress increased from *σ*_1_ = 4MPa and *σ*_3_ = 2MPa to *σ*_1_ = 18MPa and *σ*_3_ = 16MPa uniformly, the fracture evolutions are basically unchanged. The fractures initiate and propagate in the maximum principal stress direction, and then the HF dilates the BP and forms a simplex bi-wing fracture as the HFs approach the weak BP.

**Fig 9:**
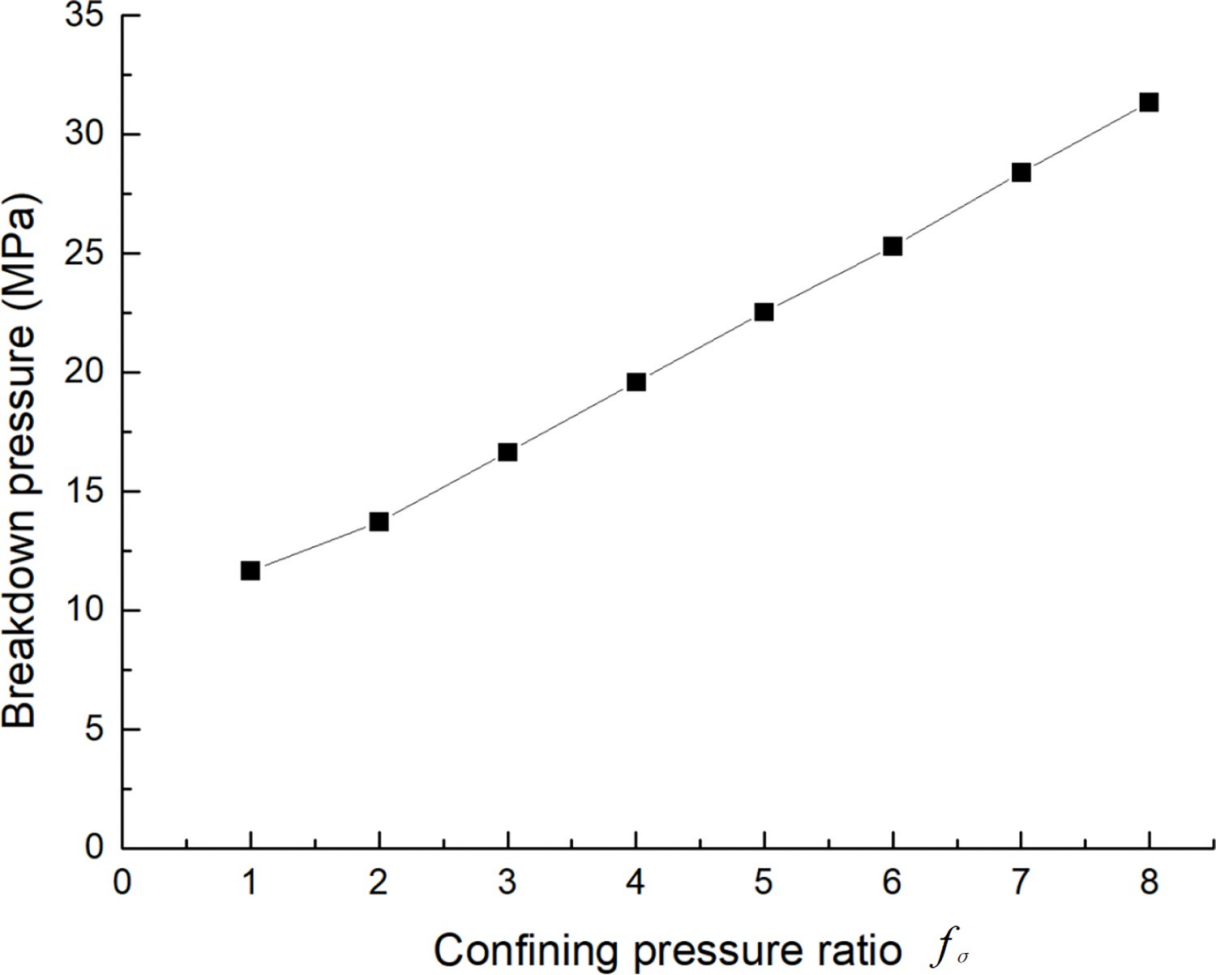
Breakdown pressure curve of models with different confining pressure ratios S2 File.

It also can be seen from Fig 8 that the breakdown pressure of the specimens increases with the absolute values of the principal stress increasing although the fracture evolution does not change. In order to describe the relationship between the absolute values of the maximum and the minimum principal stress, a confining pressure ratio $f_{\sigma }=\frac{\sigma_{3}}{\sigma_{1}‐\sigma_{3}}$ is proposed. The larger *f*_*σ*_ is, the smaller the confining pressure difference and the larger the absolute principal stress value become. As shown in Fig 9, the breakdown pressure of the specimens increases linearly with the increasing of the confining pressure ratio. In summary, it can be concluded that the absolute value of principal stress would not affect the interaction between BP and HF but influence the breakdown pressure of the specimen.

## Discussions

In this study, a criterion is proposed to predict the interaction behaviors between HF and BP. In comparison with other criteria, the proposed prediction criterion considers three interactions (Dilate, Slip and Penetrate) but not only the penetration, and it is easy to use directly. In addition, other studies mostly focus on the interaction between HF and NF rather than HF and BP. Moreover, twenty simulations with different principal stress differences and intersection angles are simulated using the RFPA^2D^-Flow code which is more suitable for rock than most of other finite element codes because it considers the heterogeneity of materials. The simulation results exhibit good agreement with the criterion results for a wide range of angles. In order to study the influence of the absolute value of principal stress on interaction, eight simulations with the same principal stress difference and intersection angle but different absolute values of the principal stress are simulated. The simulation results show that the absolute value of principal stress would not affect the interaction between BP and HF, which normalizes the application of prediction criterion. This study can provide relevant reference about the formation of complex fracture networks in shale layers.

### Conclusions

1. A criterion is proposed to predict the interaction behavior between HF and BP. The criterion is the function of the principal stress difference and the intersection angle, and it is consistent with the RFPA simulations in the verification. The criterion can provide relevant reference about the formation of complex fracture networks in the shale layer and help determine efficient fracturing scenarios and production designs in field operations.
2. HFs propagate due to tensile stress, but the AE events showed that tensile failure is the main but not the only form of failure in hydraulic fracturing in the shale layer. Compression failure is rare but also exists.
3. The smaller the intersection angle and principal stress difference are, the easier the BP dilation by the HF will be; the larger the intersection angle and principal stress difference are, the easier the BP be penetration by the HF will be. The BP shear slip can form a complex fracture network through the deflection of HFs and the penetration of beddings, helping improve the reservoir permeability.

## Supporting information

S1 Fig Fracture evolution of numerical simulation of layered shale fracturing experiment. (ZIP)

S2 Fig Fracture evolutions of numerical simulations for verifying the prediction criterion. (ZIP)

S3 Fig Fracture evolutions of models with different σ_1_ and σ_3_ but same θ and △σ. (ZIP)

S1 File Comparison of criterion and numerical results. (XLSX)

S2 File Breakdown pressure curve of models with different confining pressure ratios. (XLSX)
