# Supplementary figures and images for: Prediction criterion and numerical validation for the interaction between hydraulic fractures and bedding planes (part 1 of 2)
Source: PLoS One. 2023 Dec 21;18(12):e0294993. doi: 10.1371/journal.pone.0294993 (PMC10735180; doi:10.1371/journal.pone.0294993)

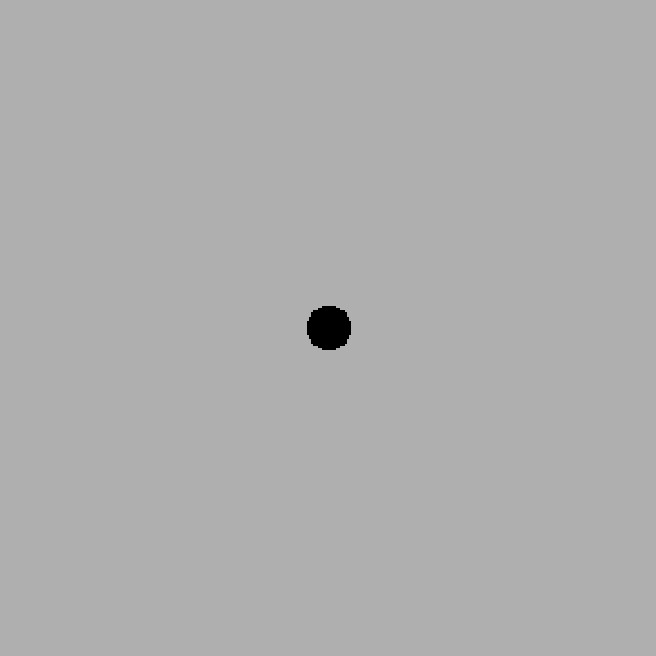

Supplement: S1 Fig — (ZIP) [file pone.0294993.s001.zip › S1_Fig/AEPic/0001-0001.jpg]

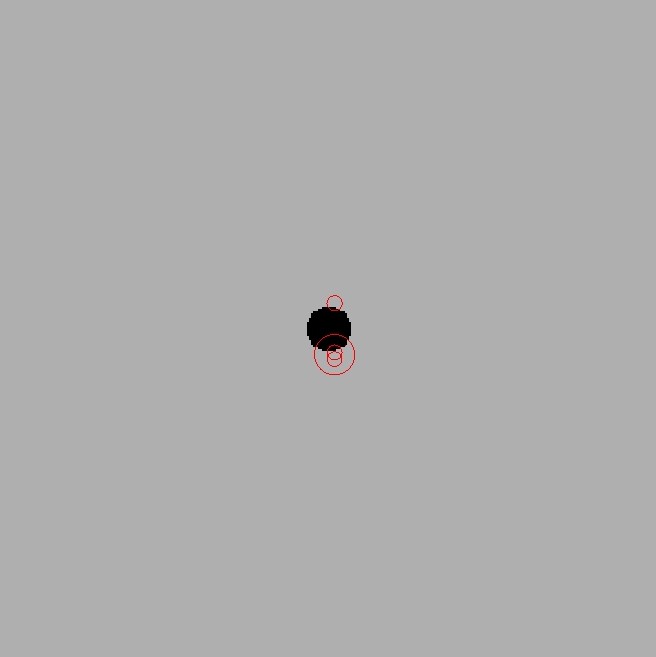

Supplement: S1 Fig — (ZIP) [file pone.0294993.s001.zip › S1_Fig/AEPic/0053-0001.jpg]

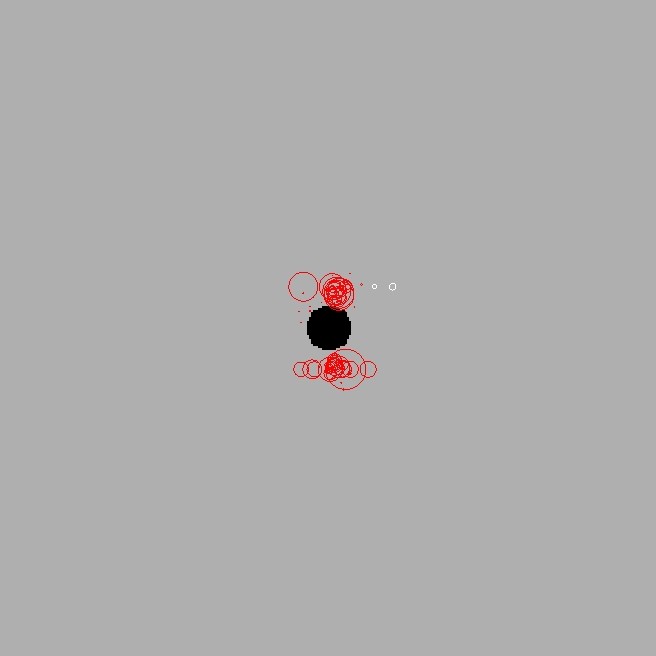

Supplement: S1 Fig — (ZIP) [file pone.0294993.s001.zip › S1_Fig/AEPic/0065-0002.jpg]

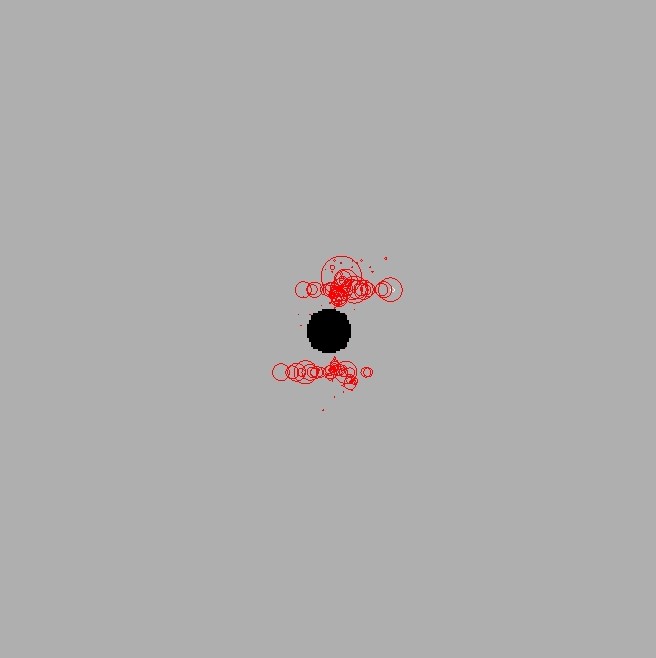

Supplement: S1 Fig — (ZIP) [file pone.0294993.s001.zip › S1_Fig/AEPic/0069-0002.jpg]

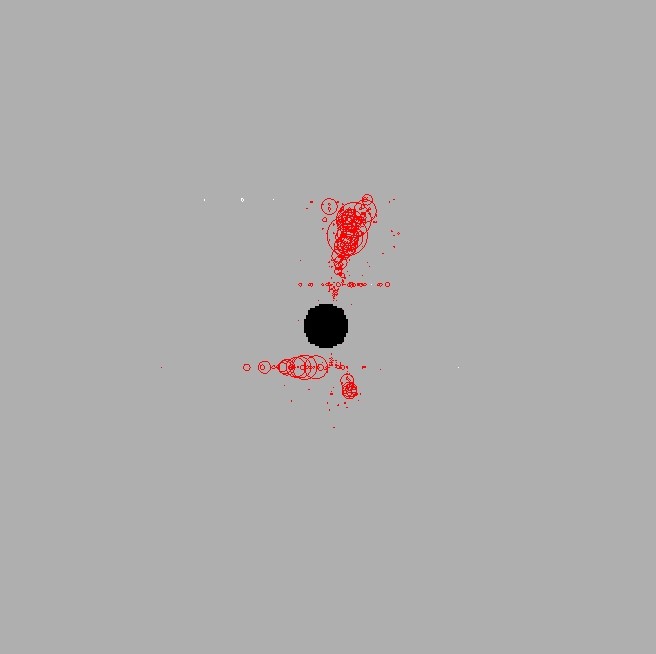

Supplement: S1 Fig — (ZIP) [file pone.0294993.s001.zip › S1_Fig/AEPic/0077-0002.jpg]

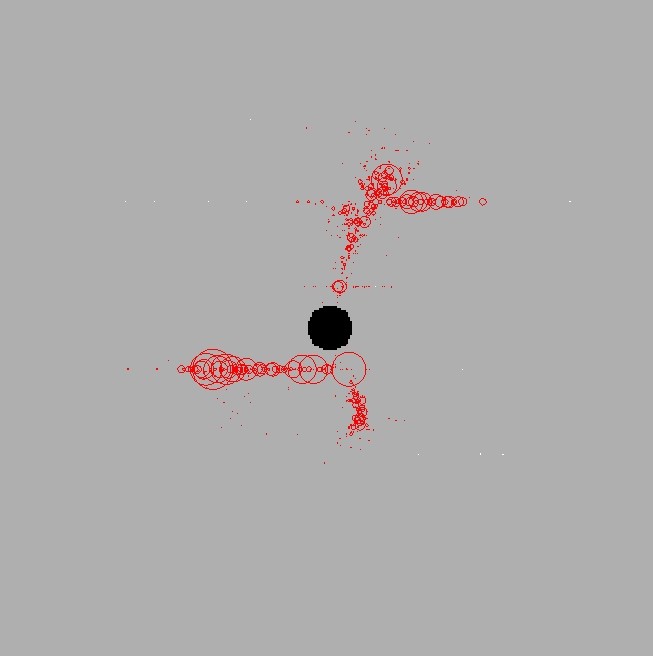

Supplement: S1 Fig — (ZIP) [file pone.0294993.s001.zip › S1_Fig/AEPic/0078-0006.jpg]

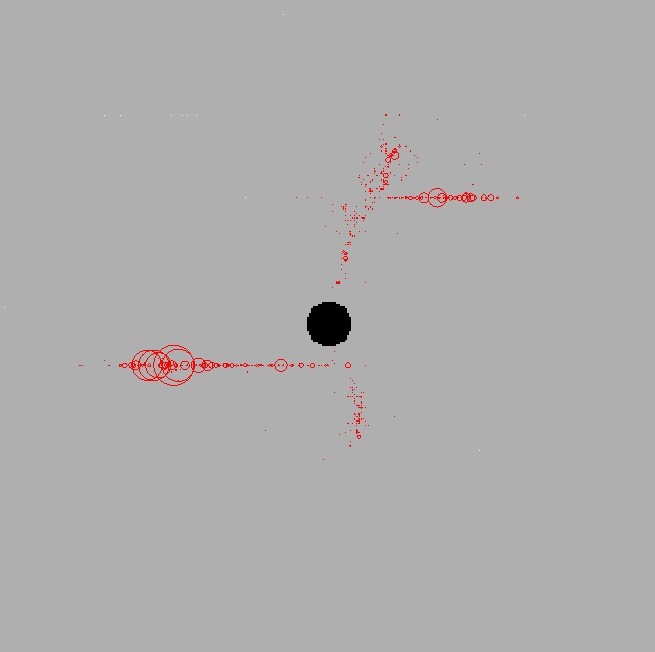

Supplement: S1 Fig — (ZIP) [file pone.0294993.s001.zip › S1_Fig/AEPic/0078-0009.jpg]

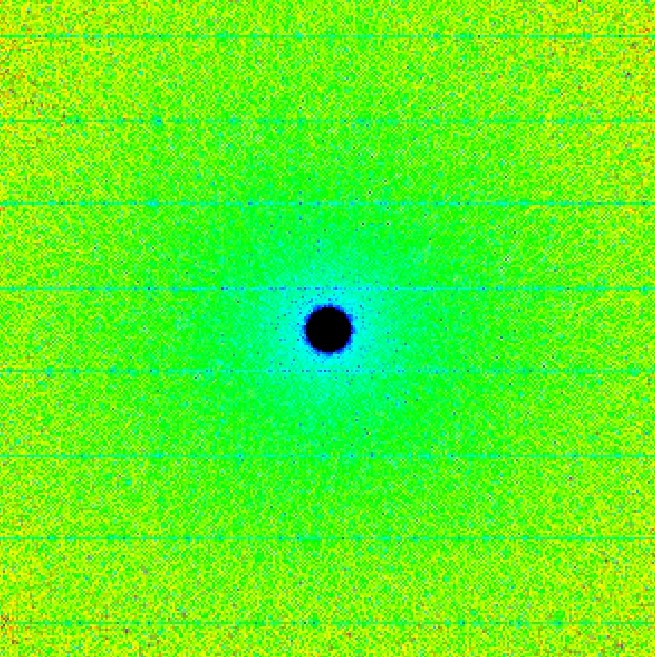

Supplement: S1 Fig — (ZIP) [file pone.0294993.s001.zip › S1_Fig/MinStressPic/0001-0001.jpg]

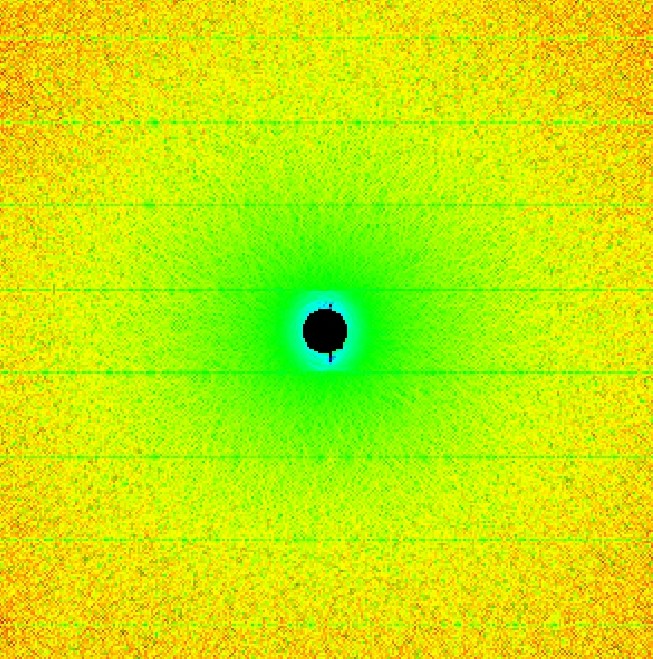

Supplement: S1 Fig — (ZIP) [file pone.0294993.s001.zip › S1_Fig/MinStressPic/0053-0001.jpg]

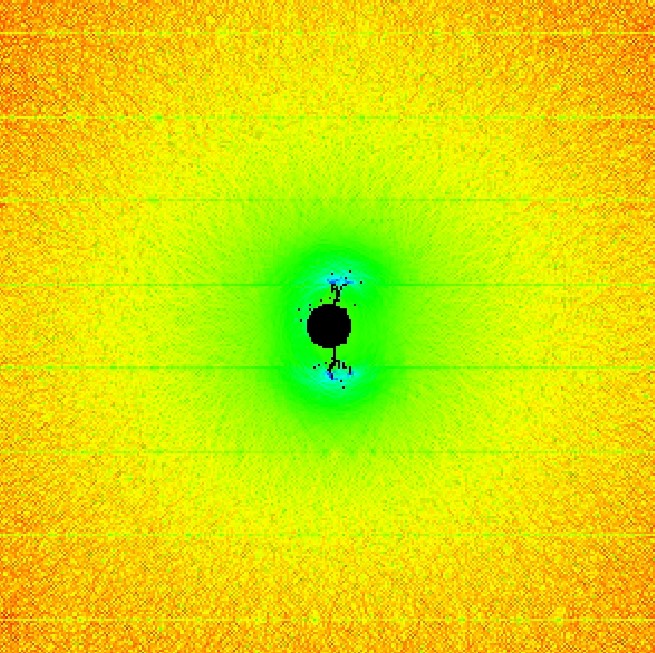

Supplement: S1 Fig — (ZIP) [file pone.0294993.s001.zip › S1_Fig/MinStressPic/0065-0002.jpg]

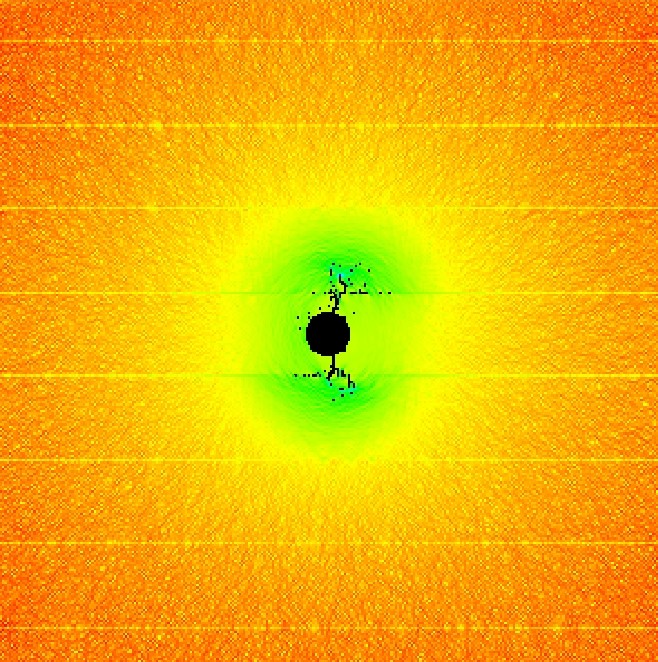

Supplement: S1 Fig — (ZIP) [file pone.0294993.s001.zip › S1_Fig/MinStressPic/0069-0002.jpg]

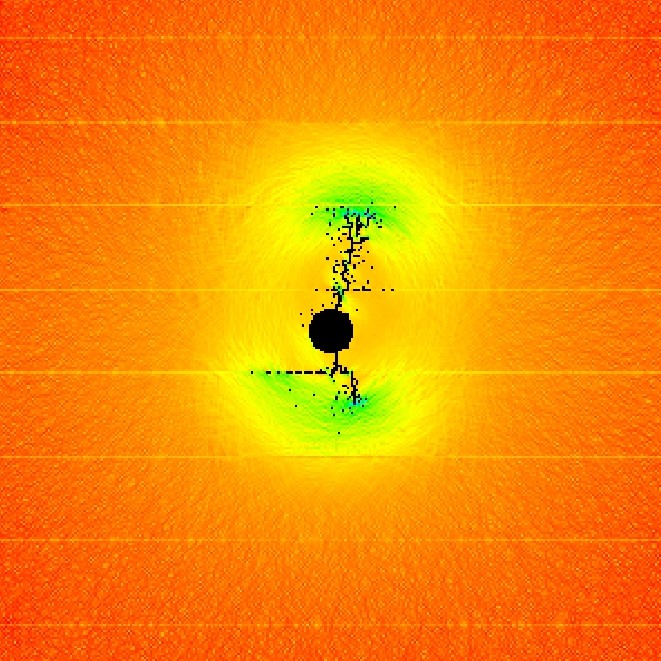

Supplement: S1 Fig — (ZIP) [file pone.0294993.s001.zip › S1_Fig/MinStressPic/0077-0002.jpg]

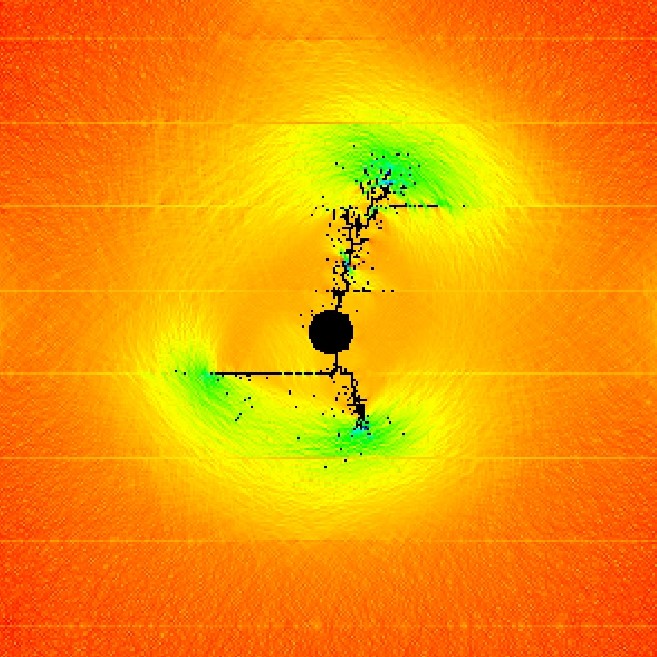

Supplement: S1 Fig — (ZIP) [file pone.0294993.s001.zip › S1_Fig/MinStressPic/0078-0006.jpg]

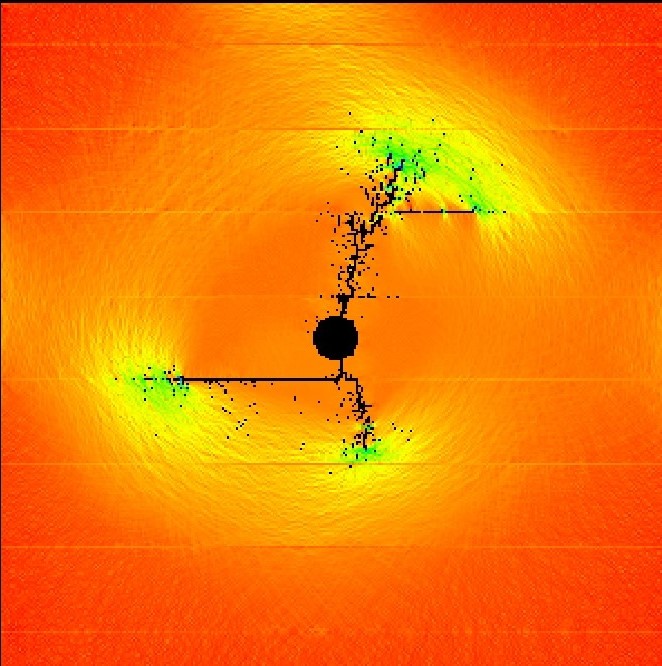

Supplement: S1 Fig — (ZIP) [file pone.0294993.s001.zip › S1_Fig/MinStressPic/0078-0009.jpg]

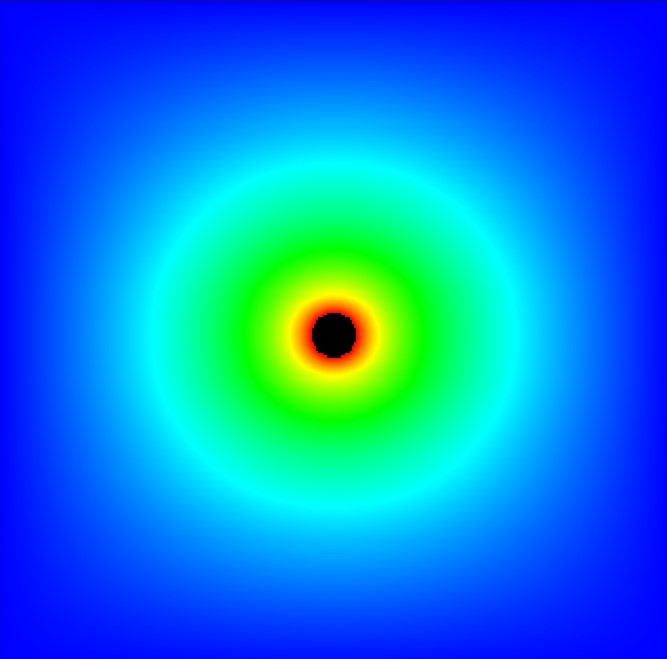

Supplement: S1 Fig — (ZIP) [file pone.0294993.s001.zip › S1_Fig/PorosityPressurePic/0001-0001.jpg]

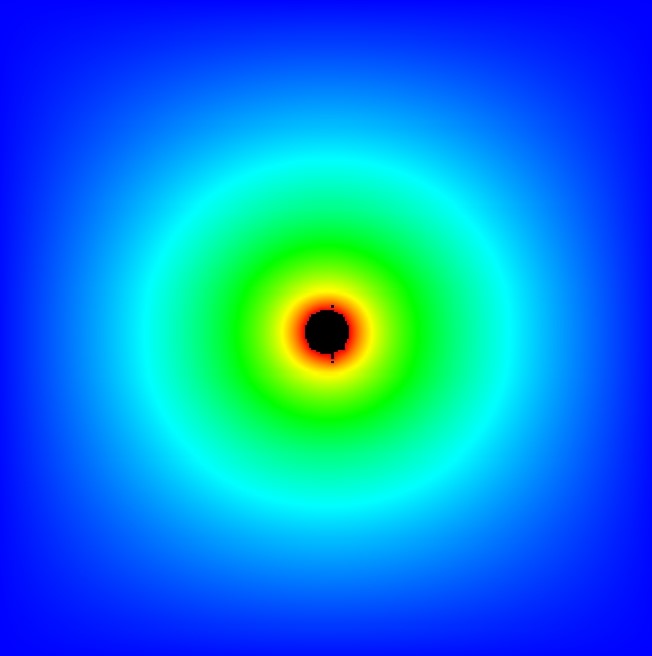

Supplement: S1 Fig — (ZIP) [file pone.0294993.s001.zip › S1_Fig/PorosityPressurePic/0053-0001.jpg]

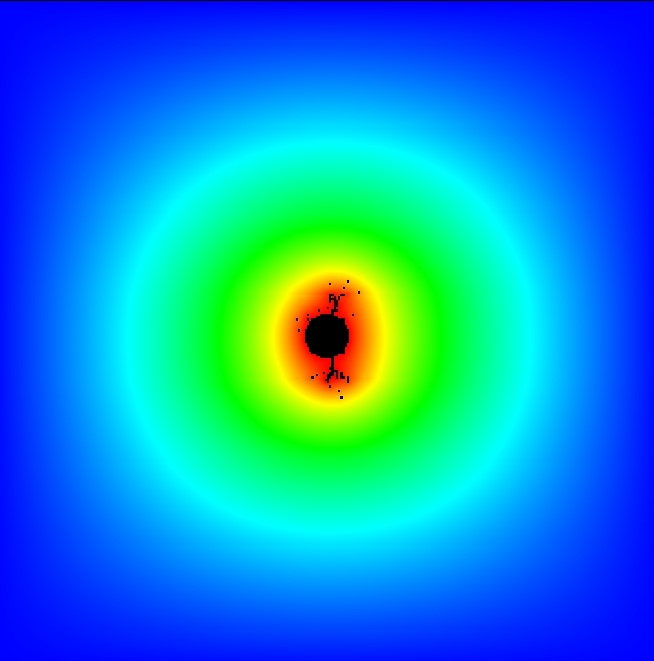

Supplement: S1 Fig — (ZIP) [file pone.0294993.s001.zip › S1_Fig/PorosityPressurePic/0065-0002.jpg]

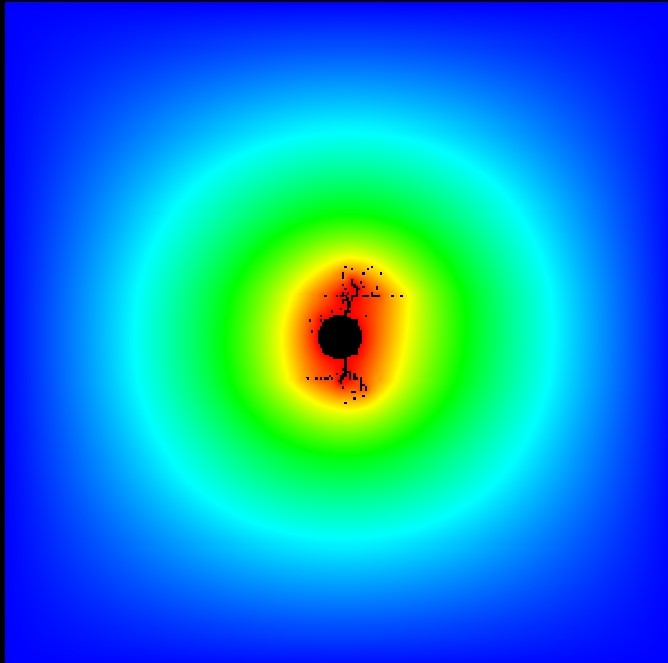

Supplement: S1 Fig — (ZIP) [file pone.0294993.s001.zip › S1_Fig/PorosityPressurePic/0069-0002.jpg]

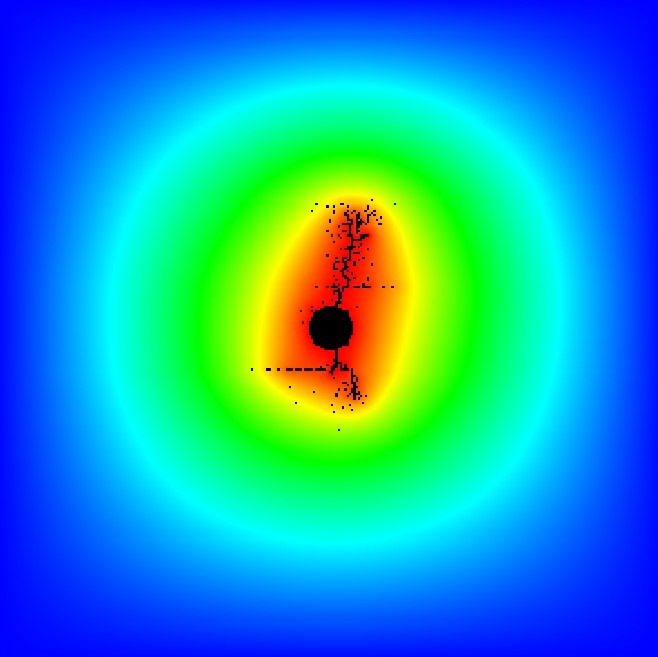

Supplement: S1 Fig — (ZIP) [file pone.0294993.s001.zip › S1_Fig/PorosityPressurePic/0077-0002.jpg]

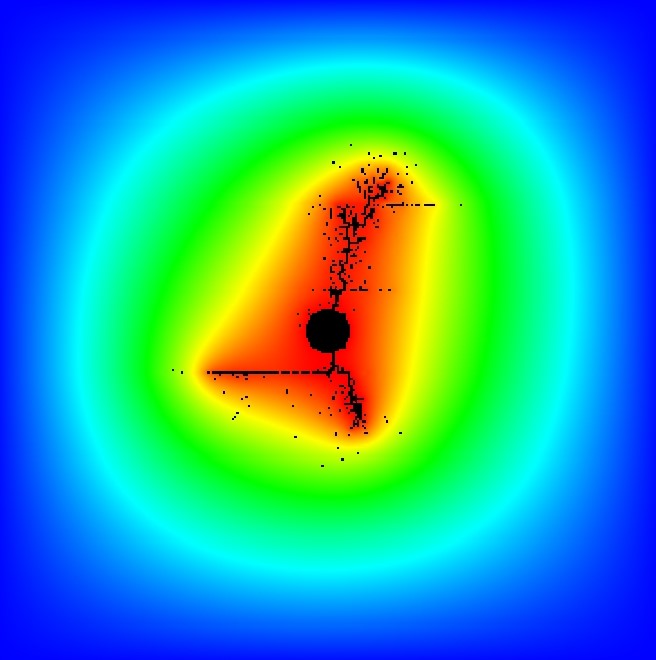

Supplement: S1 Fig — (ZIP) [file pone.0294993.s001.zip › S1_Fig/PorosityPressurePic/0078-0006.jpg]

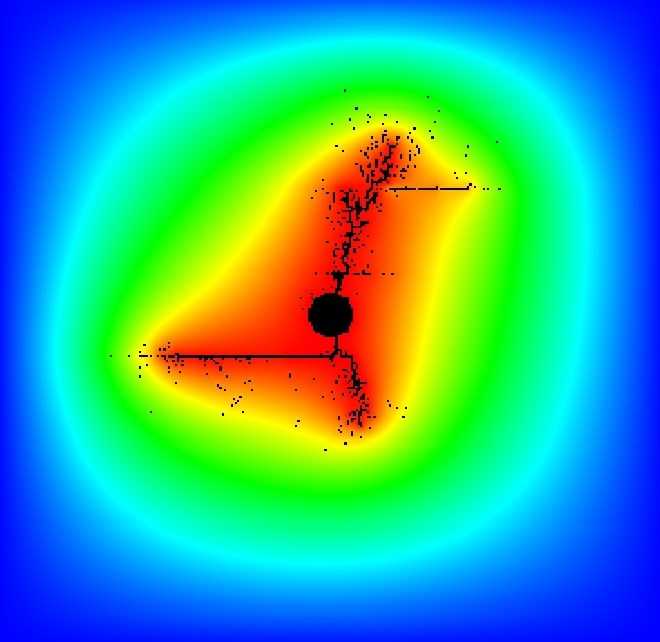

Supplement: S1 Fig — (ZIP) [file pone.0294993.s001.zip › S1_Fig/PorosityPressurePic/0078-0009.jpg]

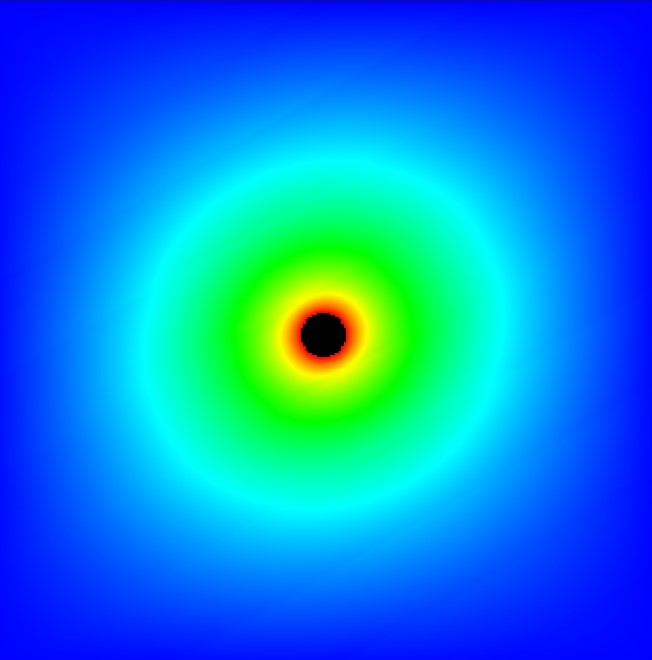

Supplement: S2 Fig — (ZIP) [file pone.0294993.s002.zip › S2_Fig/30°/△σ=2MPa/0001-0001.jpg]

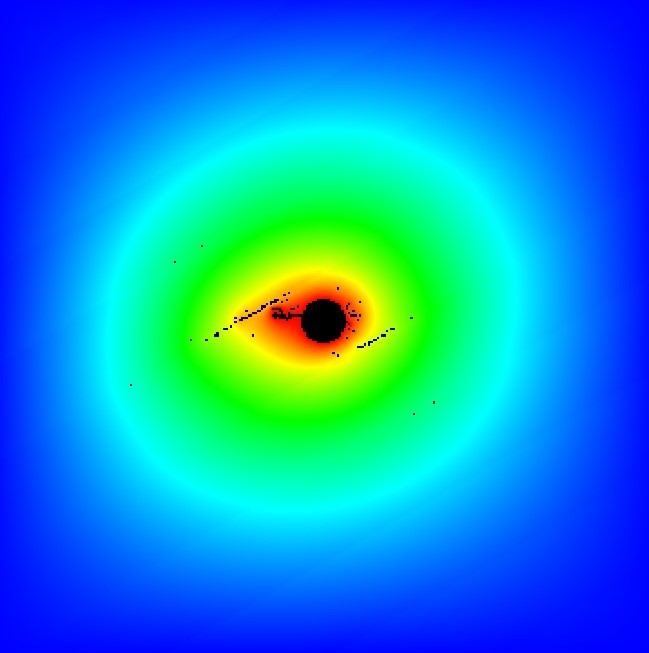

Supplement: S2 Fig — (ZIP) [file pone.0294993.s002.zip › S2_Fig/30°/△σ=2MPa/0044-0001.jpg]

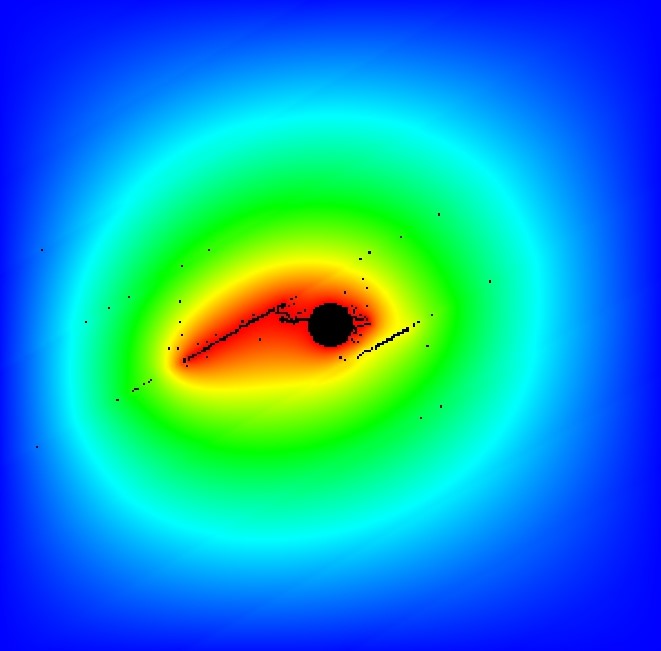

Supplement: S2 Fig — (ZIP) [file pone.0294993.s002.zip › S2_Fig/30°/△σ=2MPa/0047-0001.jpg]

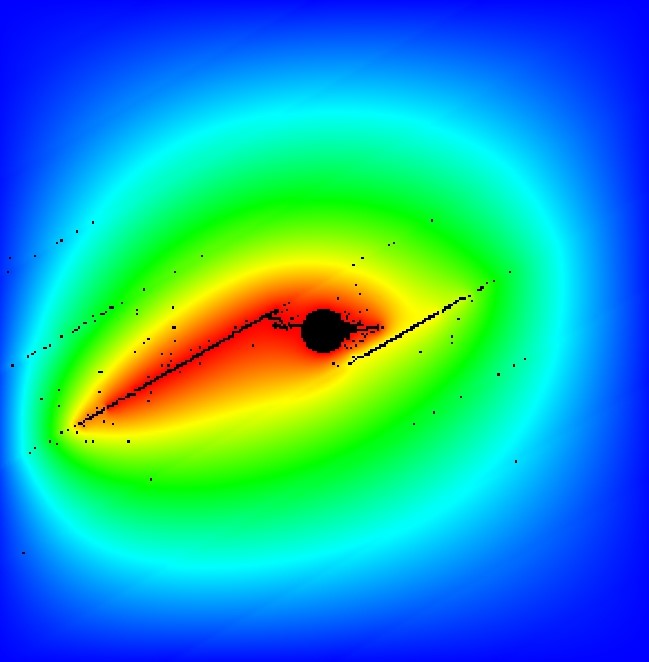

Supplement: S2 Fig — (ZIP) [file pone.0294993.s002.zip › S2_Fig/30°/△σ=2MPa/0049-0003.jpg]

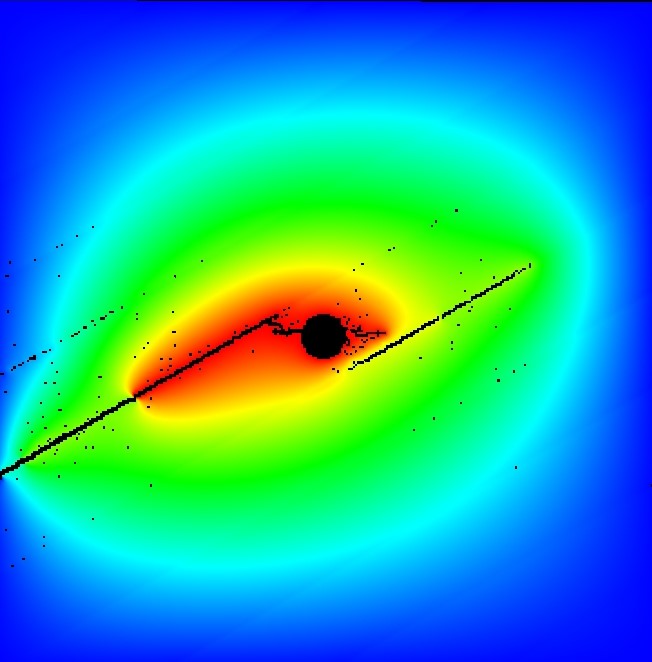

Supplement: S2 Fig — (ZIP) [file pone.0294993.s002.zip › S2_Fig/30°/△σ=2MPa/0049-0007.jpg]

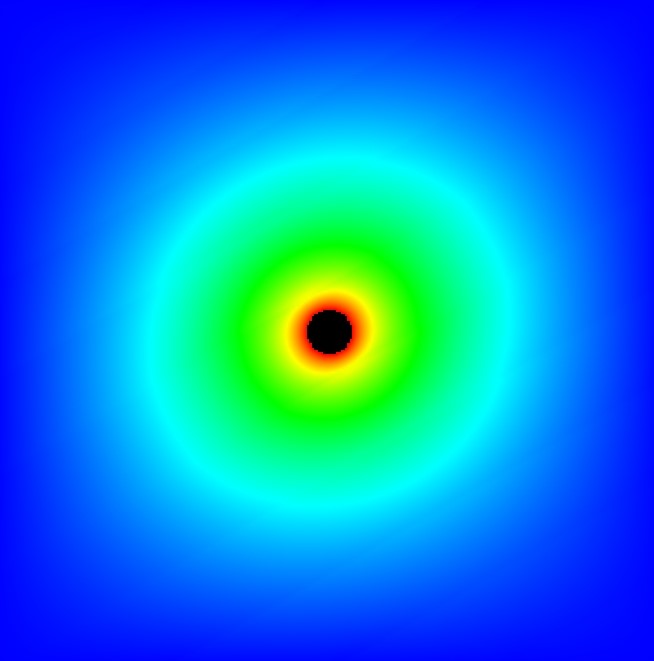

Supplement: S2 Fig — (ZIP) [file pone.0294993.s002.zip › S2_Fig/30°/△σ=4MPa/0001-0001.jpg]

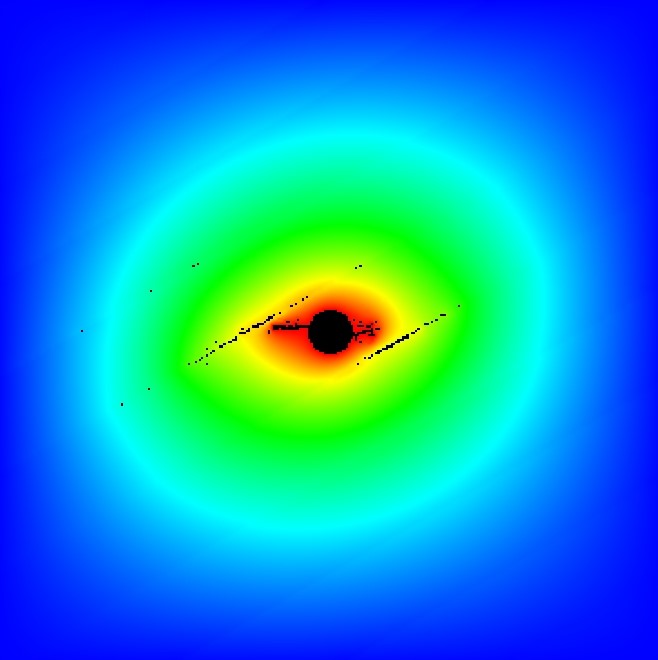

Supplement: S2 Fig — (ZIP) [file pone.0294993.s002.zip › S2_Fig/30°/△σ=4MPa/0040-0001.jpg]

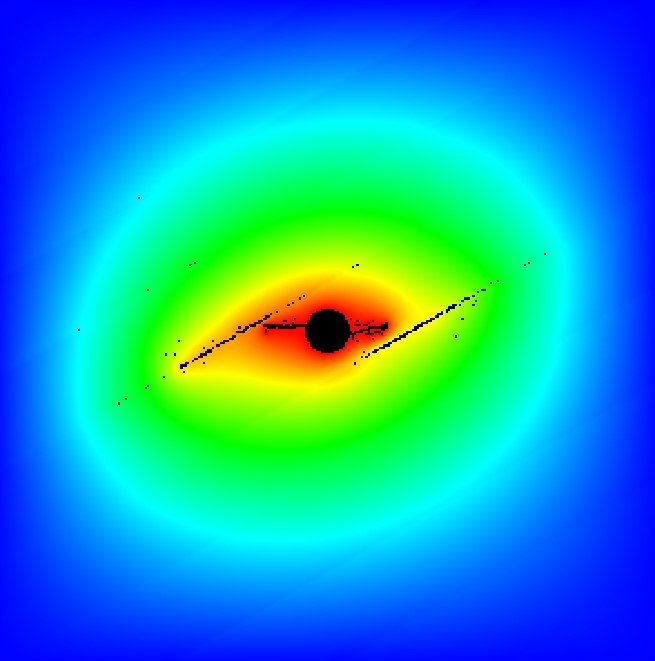

Supplement: S2 Fig — (ZIP) [file pone.0294993.s002.zip › S2_Fig/30°/△σ=4MPa/0042-0002.jpg]

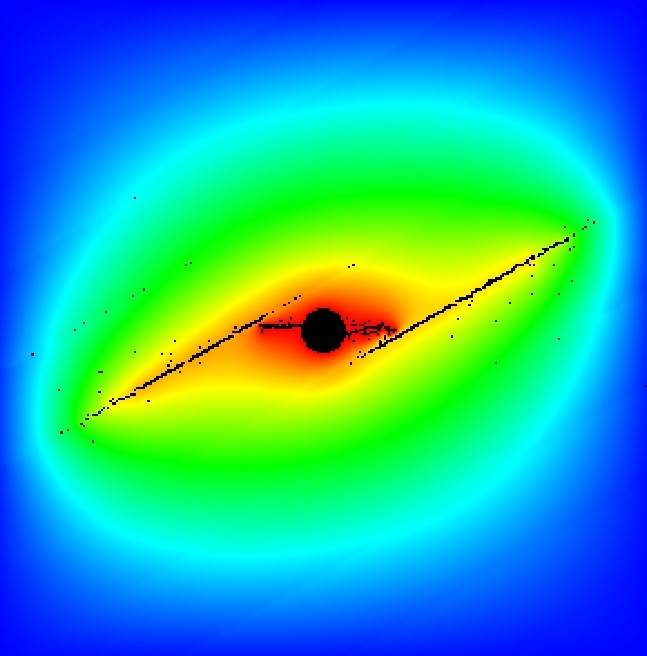

Supplement: S2 Fig — (ZIP) [file pone.0294993.s002.zip › S2_Fig/30°/△σ=4MPa/0044-0004.jpg]

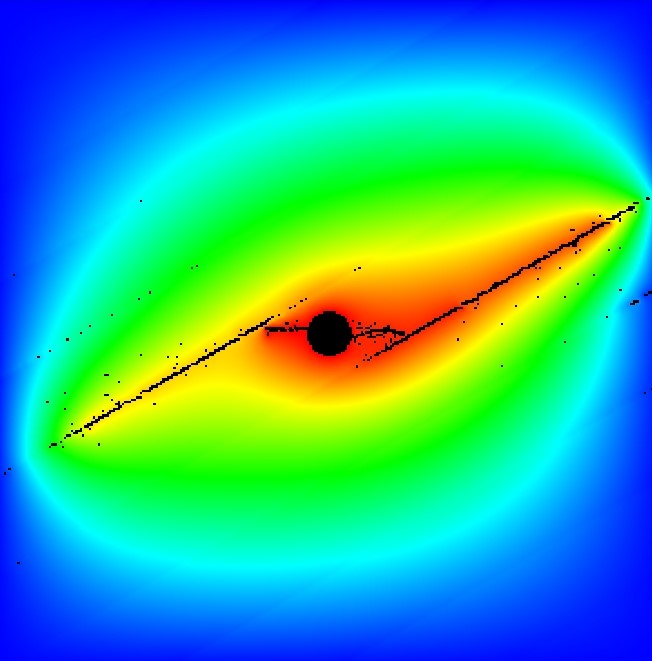

Supplement: S2 Fig — (ZIP) [file pone.0294993.s002.zip › S2_Fig/30°/△σ=4MPa/0045-0004.jpg]

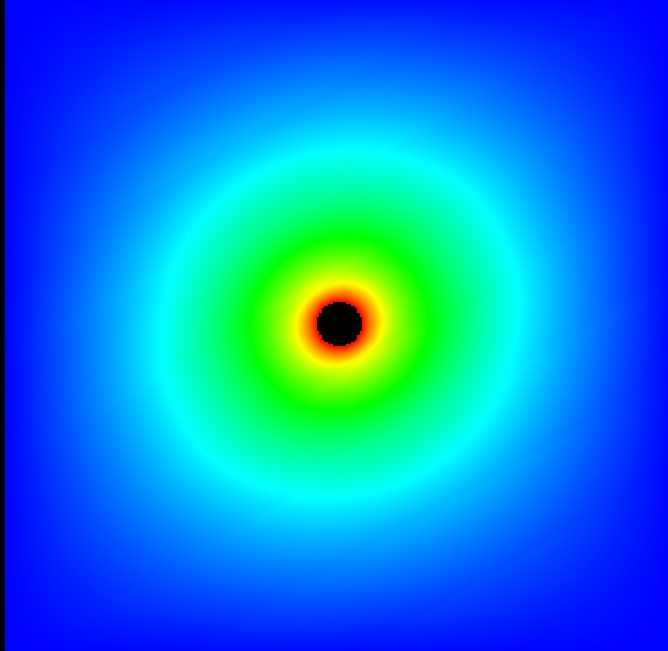

Supplement: S2 Fig — (ZIP) [file pone.0294993.s002.zip › S2_Fig/30°/△σ=6MPa/0001-0001.jpg]

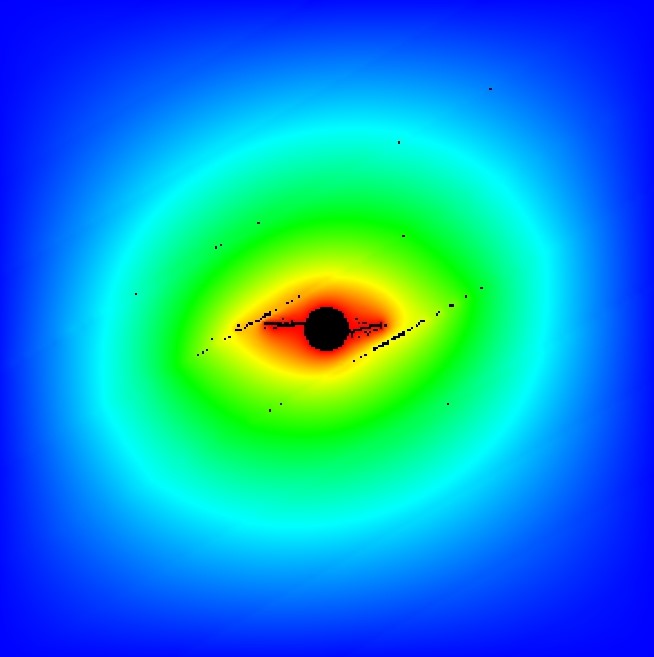

Supplement: S2 Fig — (ZIP) [file pone.0294993.s002.zip › S2_Fig/30°/△σ=6MPa/0043-0002.jpg]

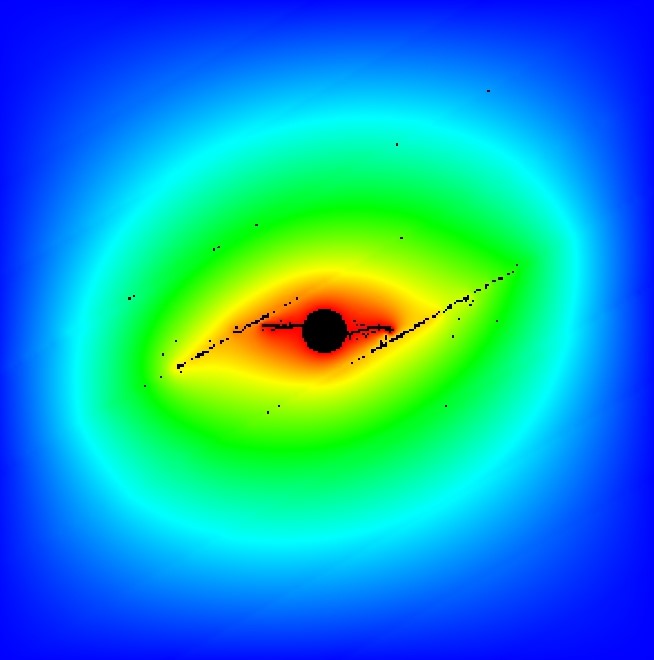

Supplement: S2 Fig — (ZIP) [file pone.0294993.s002.zip › S2_Fig/30°/△σ=6MPa/0045-0007.jpg]

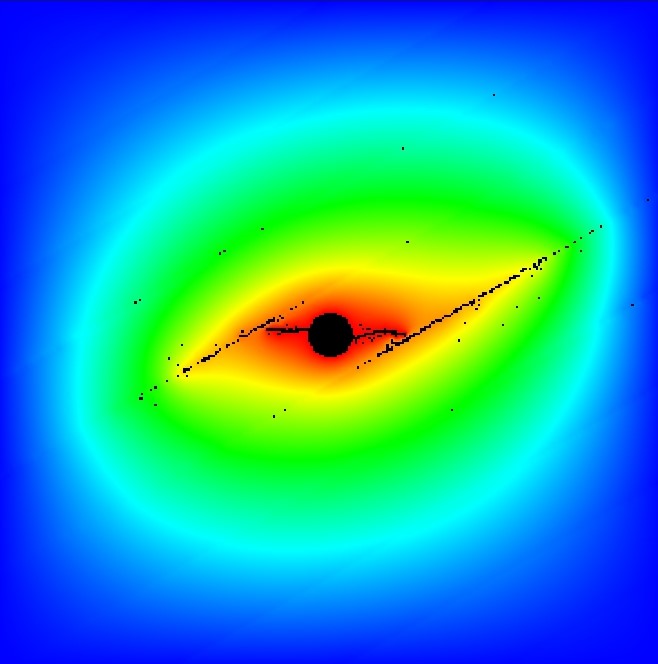

Supplement: S2 Fig — (ZIP) [file pone.0294993.s002.zip › S2_Fig/30°/△σ=6MPa/0046-0004.jpg]

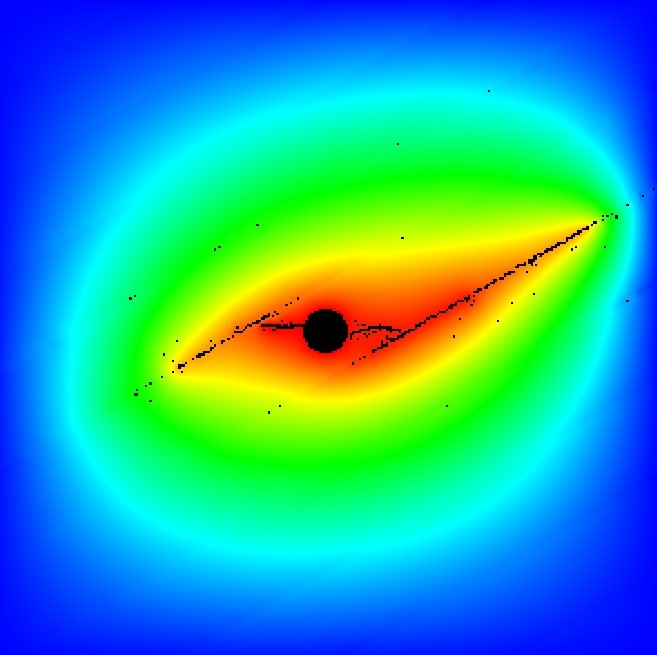

Supplement: S2 Fig — (ZIP) [file pone.0294993.s002.zip › S2_Fig/30°/△σ=6MPa/0046-0008.jpg]

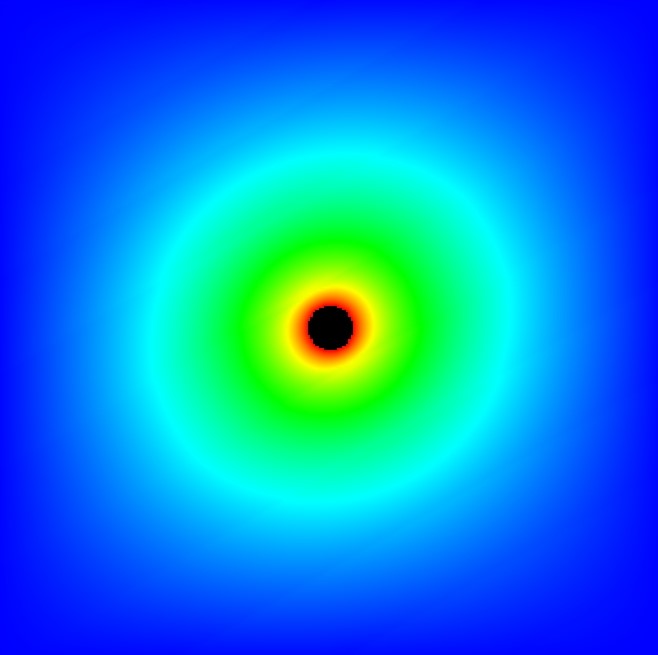

Supplement: S2 Fig — (ZIP) [file pone.0294993.s002.zip › S2_Fig/30°/△σ=8MPa/0001-0001.jpg]

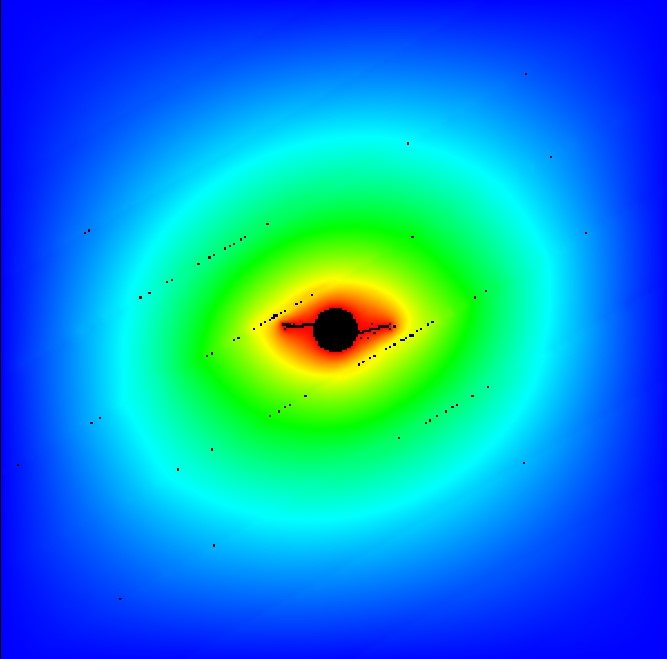

Supplement: S2 Fig — (ZIP) [file pone.0294993.s002.zip › S2_Fig/30°/△σ=8MPa/0033-0001.jpg]

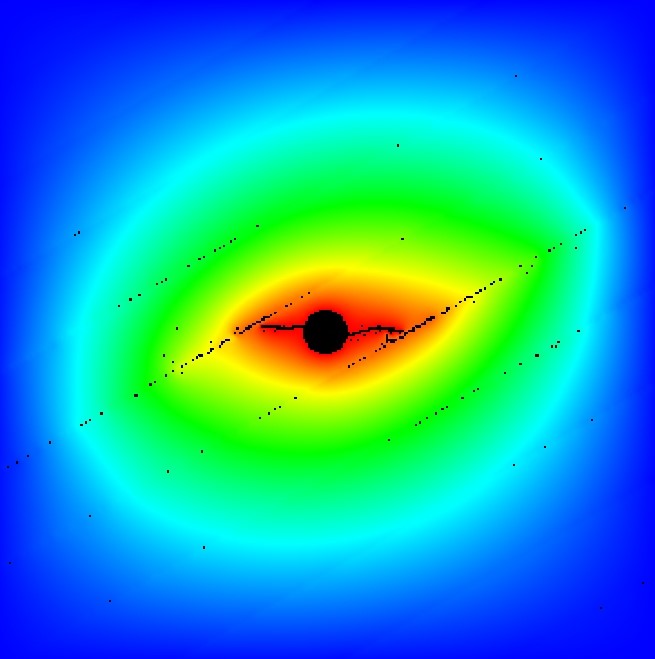

Supplement: S2 Fig — (ZIP) [file pone.0294993.s002.zip › S2_Fig/30°/△σ=8MPa/0039-0002.jpg]

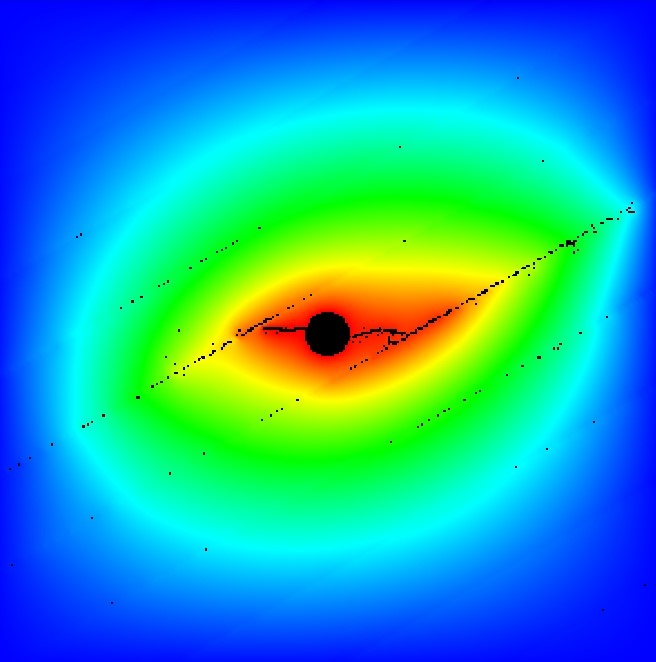

Supplement: S2 Fig — (ZIP) [file pone.0294993.s002.zip › S2_Fig/30°/△σ=8MPa/0040-0002.jpg]

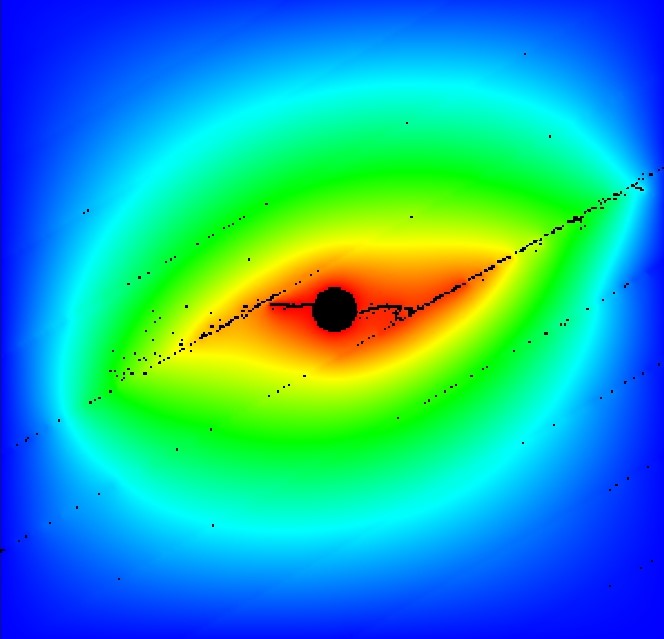

Supplement: S2 Fig — (ZIP) [file pone.0294993.s002.zip › S2_Fig/30°/△σ=8MPa/0042-0007.jpg]

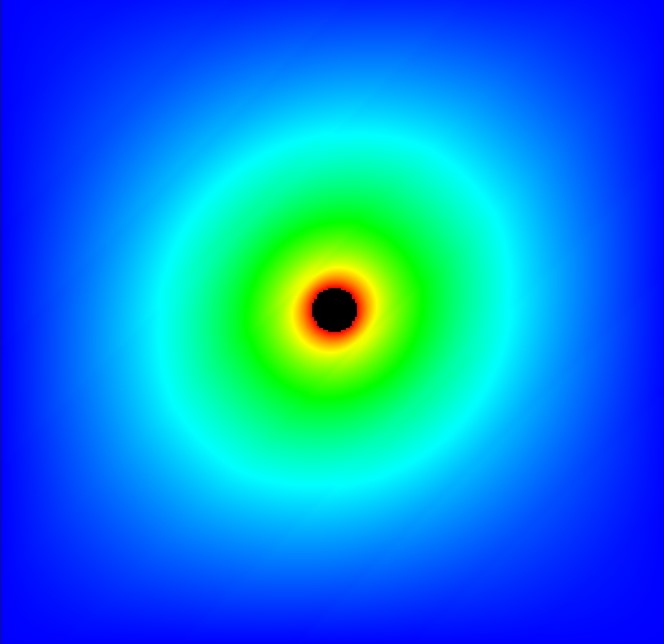

Supplement: S2 Fig — (ZIP) [file pone.0294993.s002.zip › S2_Fig/45°/△σ=2MPa/0001-0001.jpg]

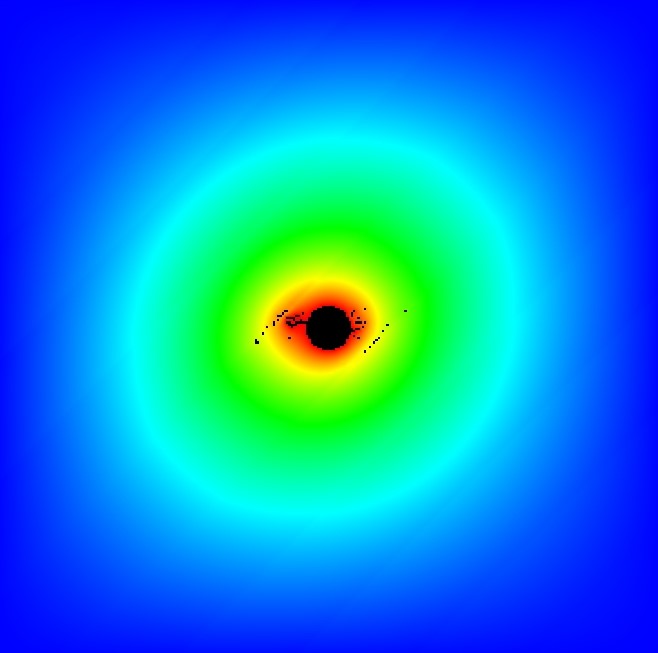

Supplement: S2 Fig — (ZIP) [file pone.0294993.s002.zip › S2_Fig/45°/△σ=2MPa/0032-0004.jpg]

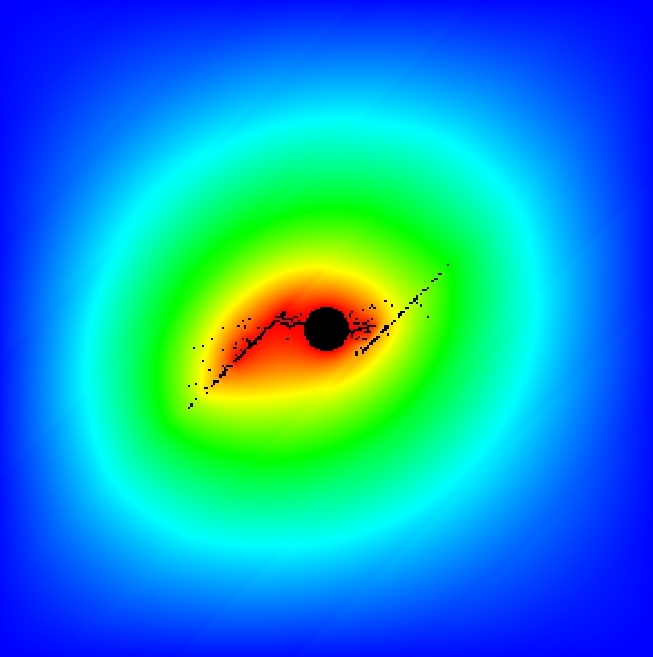

Supplement: S2 Fig — (ZIP) [file pone.0294993.s002.zip › S2_Fig/45°/△σ=2MPa/0032-0008.jpg]

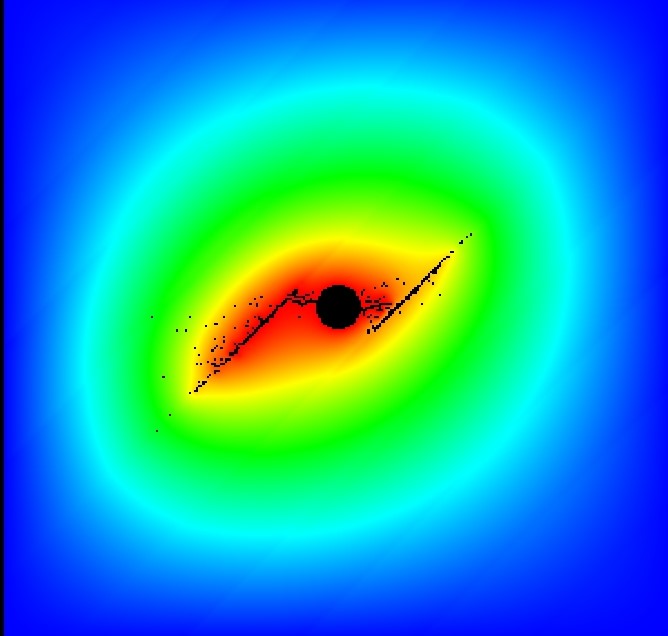

Supplement: S2 Fig — (ZIP) [file pone.0294993.s002.zip › S2_Fig/45°/△σ=2MPa/0032-0009.jpg]

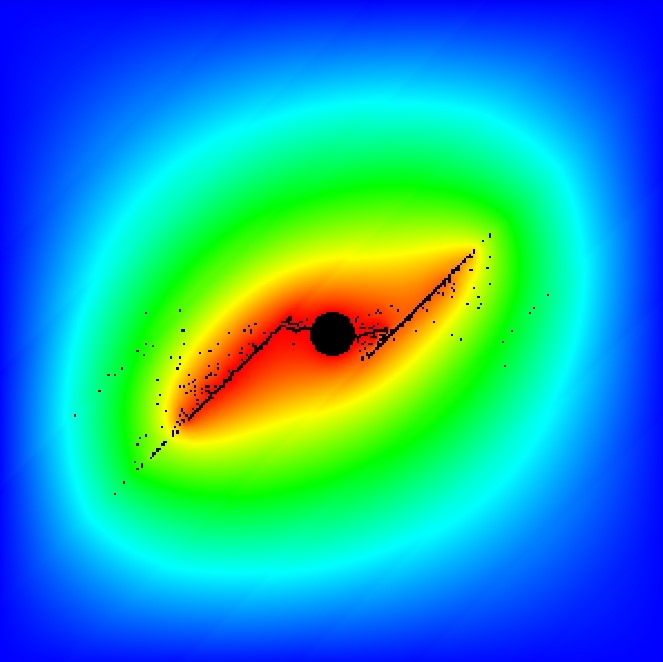

Supplement: S2 Fig — (ZIP) [file pone.0294993.s002.zip › S2_Fig/45°/△σ=2MPa/0032-0010.jpg]

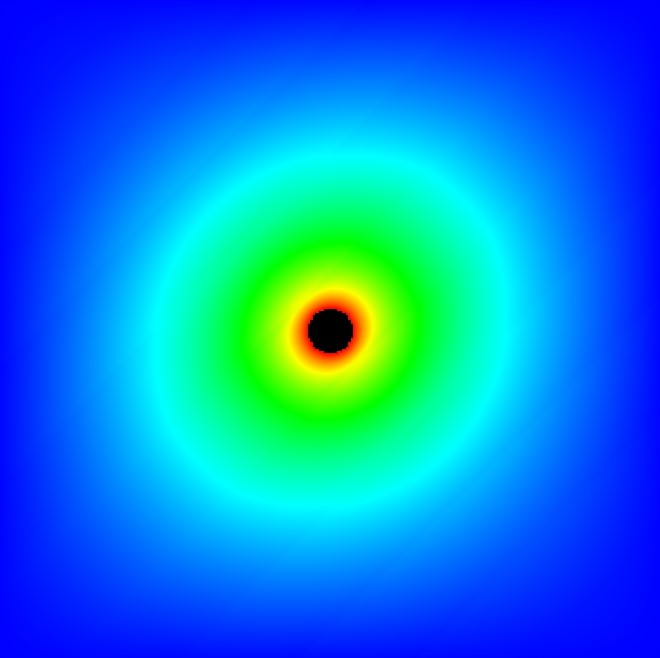

Supplement: S2 Fig — (ZIP) [file pone.0294993.s002.zip › S2_Fig/45°/△σ=4MPa/0001-0001.jpg]

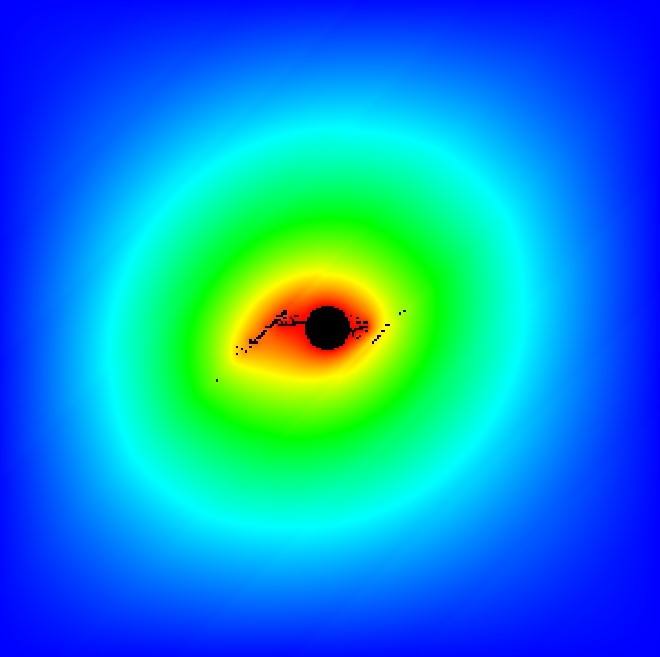

Supplement: S2 Fig — (ZIP) [file pone.0294993.s002.zip › S2_Fig/45°/△σ=4MPa/0026-0005.jpg]

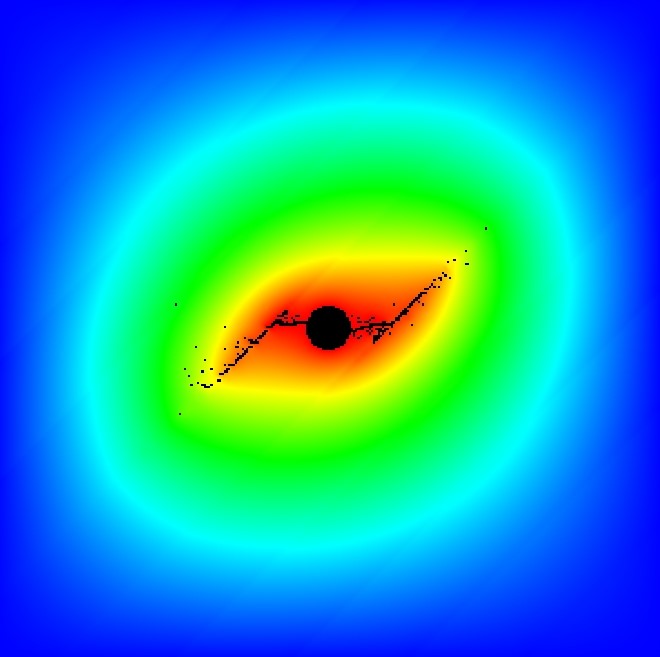

Supplement: S2 Fig — (ZIP) [file pone.0294993.s002.zip › S2_Fig/45°/△σ=4MPa/0026-0010.jpg]

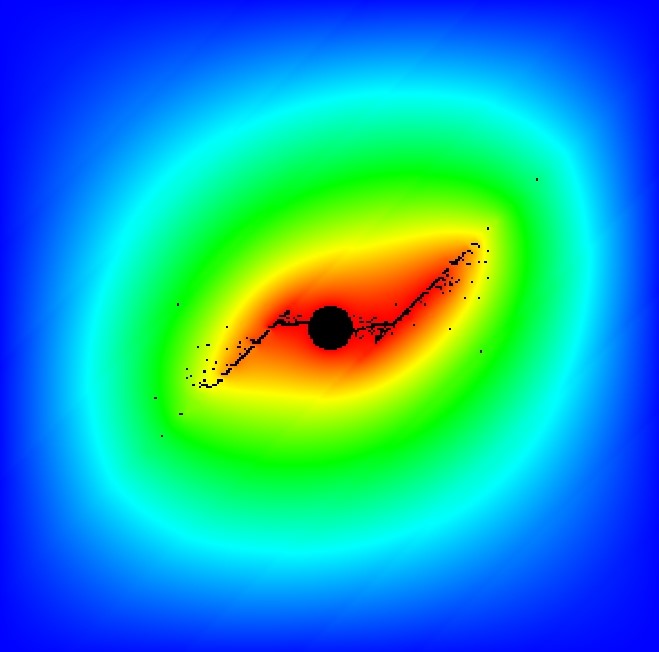

Supplement: S2 Fig — (ZIP) [file pone.0294993.s002.zip › S2_Fig/45°/△σ=4MPa/0026-0011.jpg]

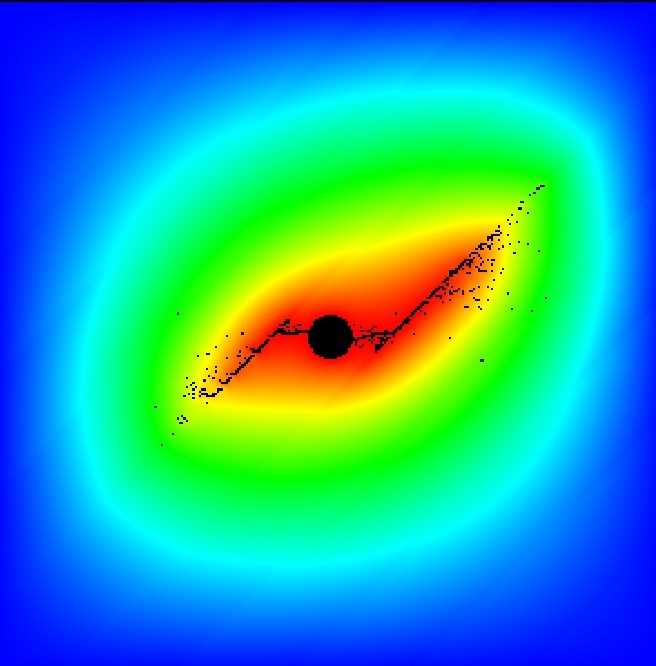

Supplement: S2 Fig — (ZIP) [file pone.0294993.s002.zip › S2_Fig/45°/△σ=4MPa/0026-0012.jpg]

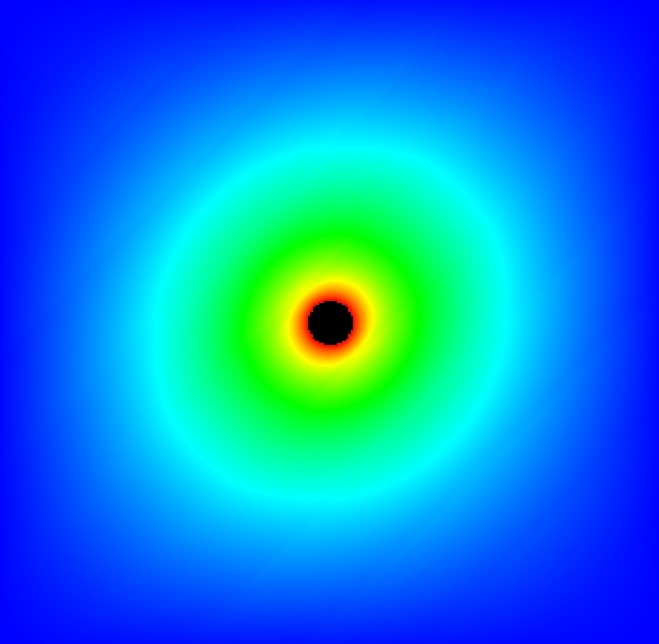

Supplement: S2 Fig — (ZIP) [file pone.0294993.s002.zip › S2_Fig/45°/△σ=6MPa/0001-0001.jpg]

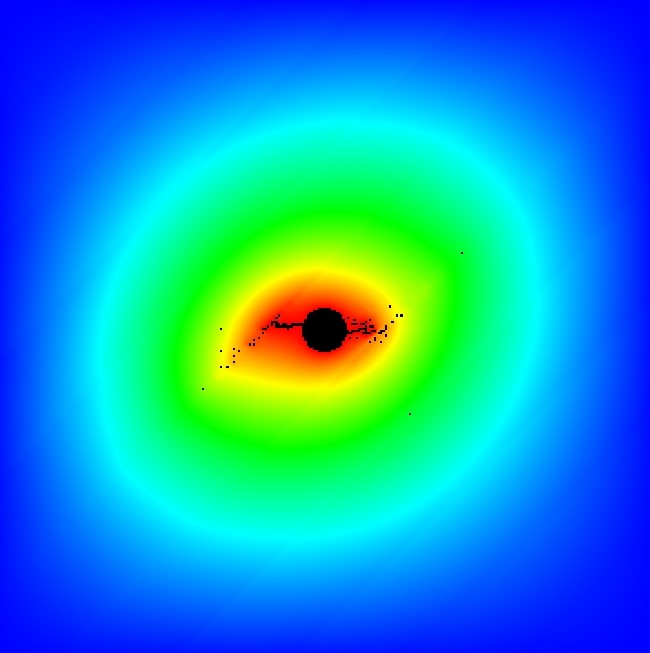

Supplement: S2 Fig — (ZIP) [file pone.0294993.s002.zip › S2_Fig/45°/△σ=6MPa/0027-0001.jpg]

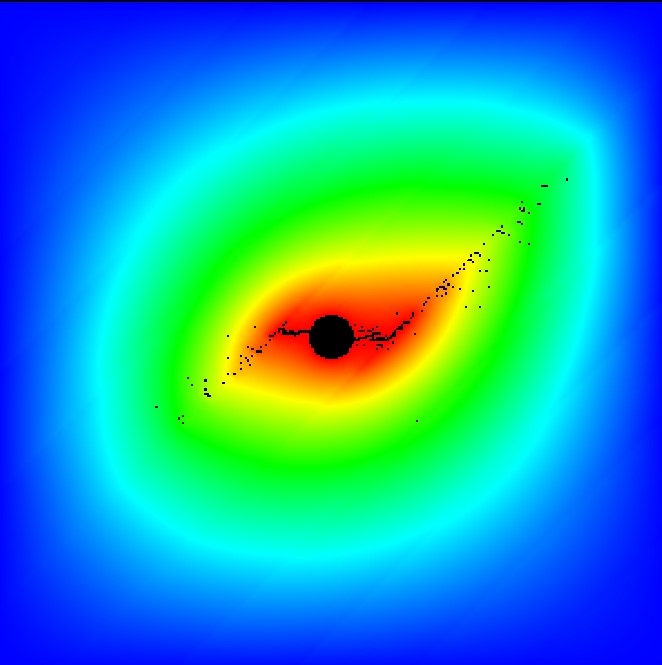

Supplement: S2 Fig — (ZIP) [file pone.0294993.s002.zip › S2_Fig/45°/△σ=6MPa/0028-0008.jpg]

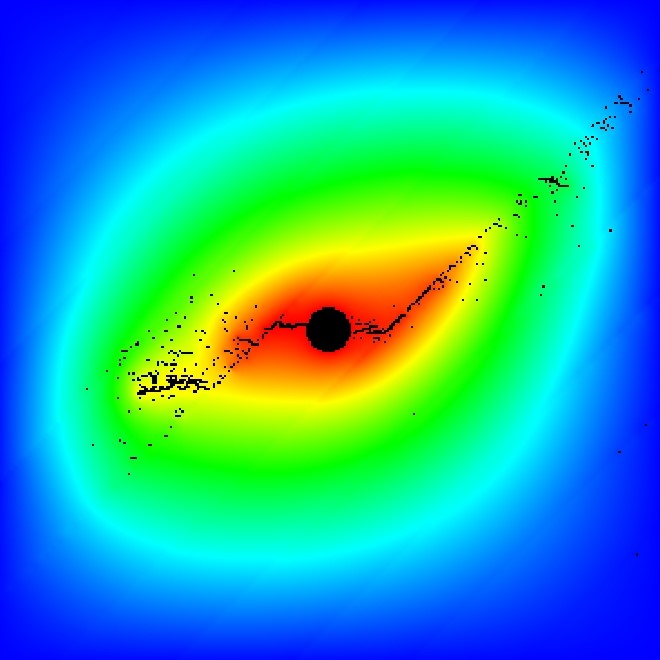

Supplement: S2 Fig — (ZIP) [file pone.0294993.s002.zip › S2_Fig/45°/△σ=6MPa/0032-0005.jpg]

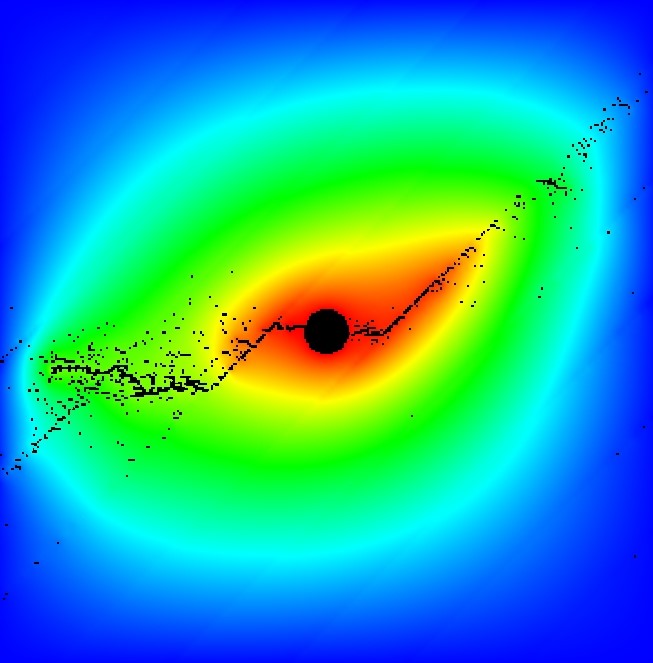

Supplement: S2 Fig — (ZIP) [file pone.0294993.s002.zip › S2_Fig/45°/△σ=6MPa/0036-0003.jpg]

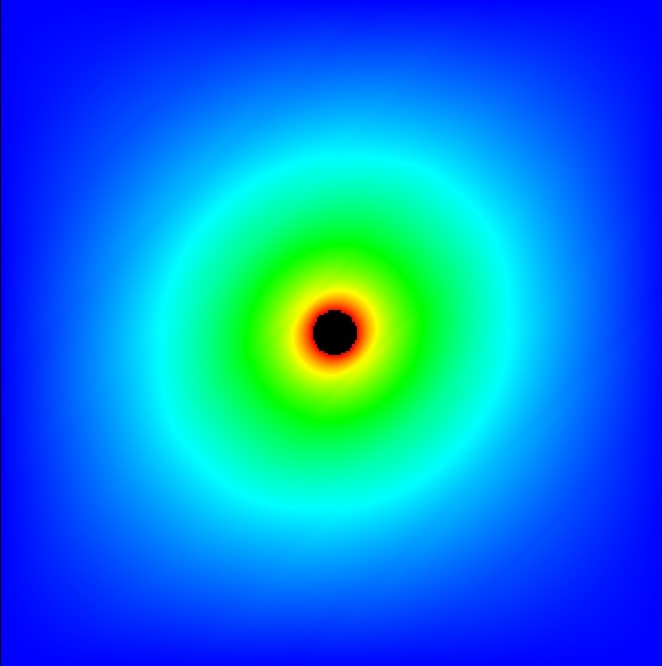

Supplement: S2 Fig — (ZIP) [file pone.0294993.s002.zip › S2_Fig/45°/△σ=8MPa/0001-0001.jpg]

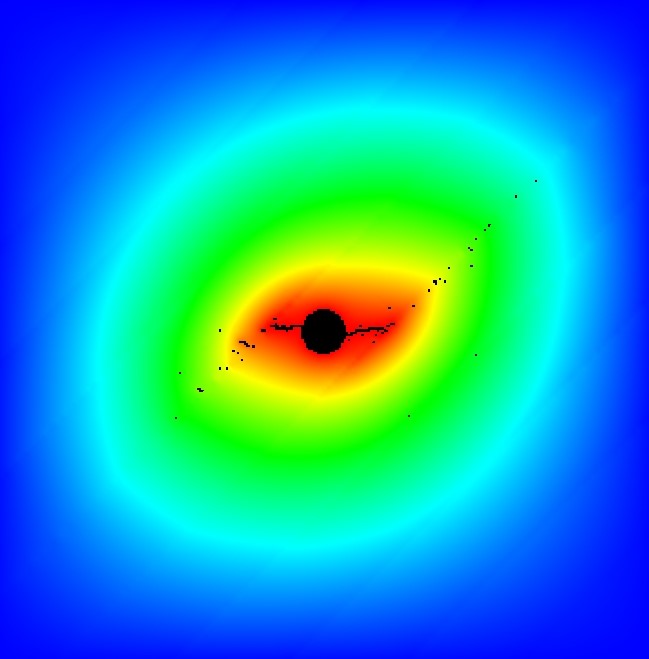

Supplement: S2 Fig — (ZIP) [file pone.0294993.s002.zip › S2_Fig/45°/△σ=8MPa/0034-0001.jpg]

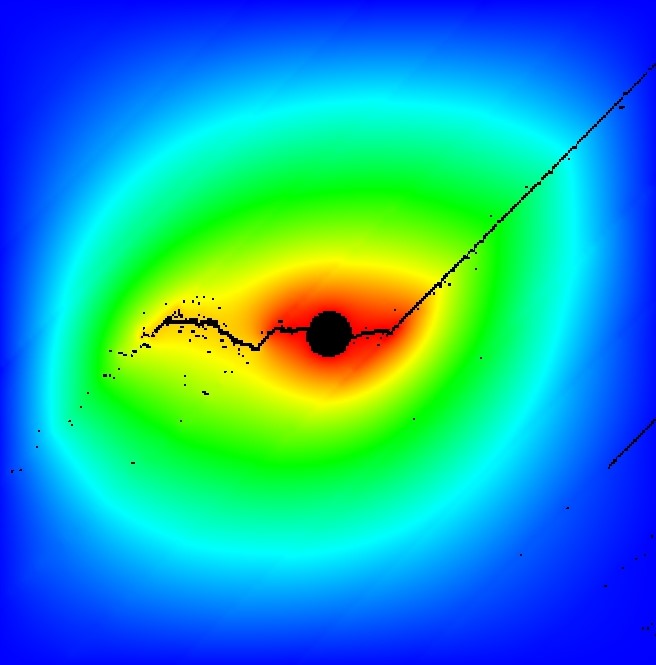

Supplement: S2 Fig — (ZIP) [file pone.0294993.s002.zip › S2_Fig/45°/△σ=8MPa/0038-0012.jpg]

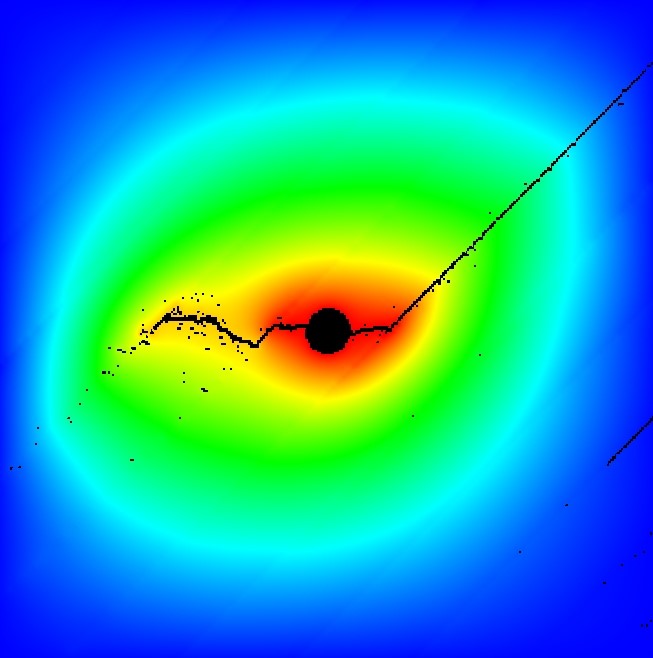

Supplement: S2 Fig — (ZIP) [file pone.0294993.s002.zip › S2_Fig/45°/△σ=8MPa/0040-0001.jpg]

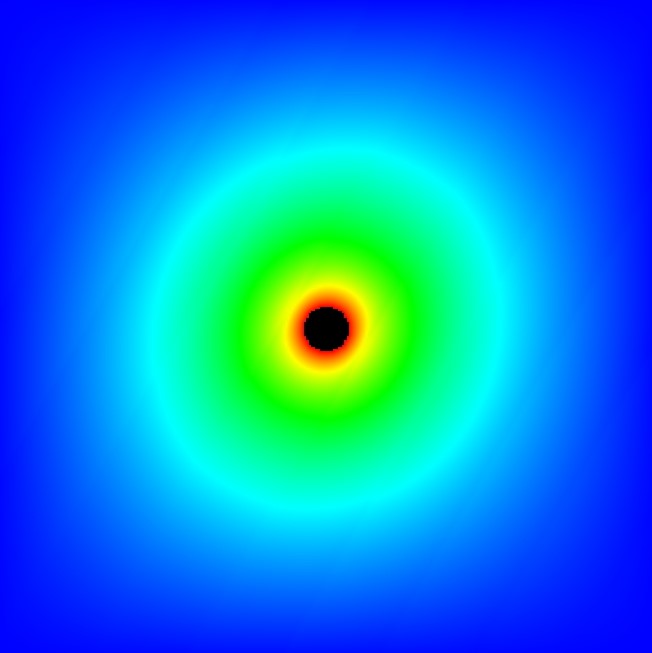

Supplement: S2 Fig — (ZIP) [file pone.0294993.s002.zip › S2_Fig/60°/△σ=2MPa/0001-0001.jpg]

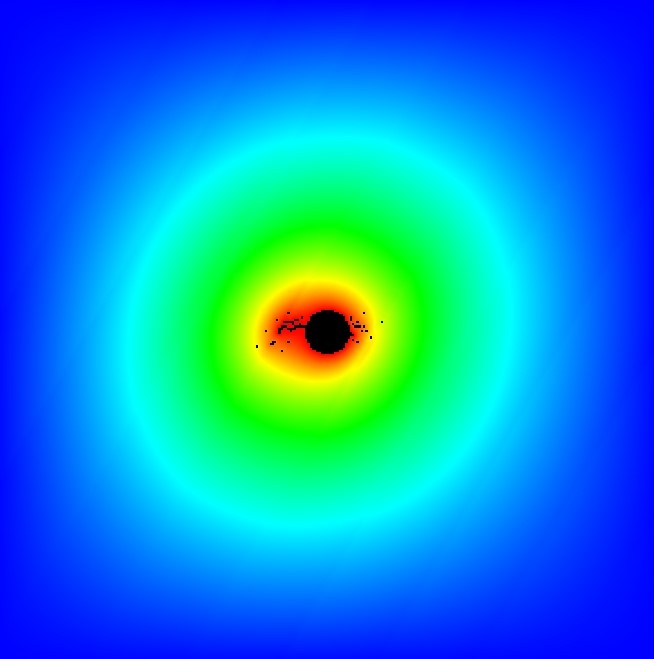

Supplement: S2 Fig — (ZIP) [file pone.0294993.s002.zip › S2_Fig/60°/△σ=2MPa/0022-0004.jpg]

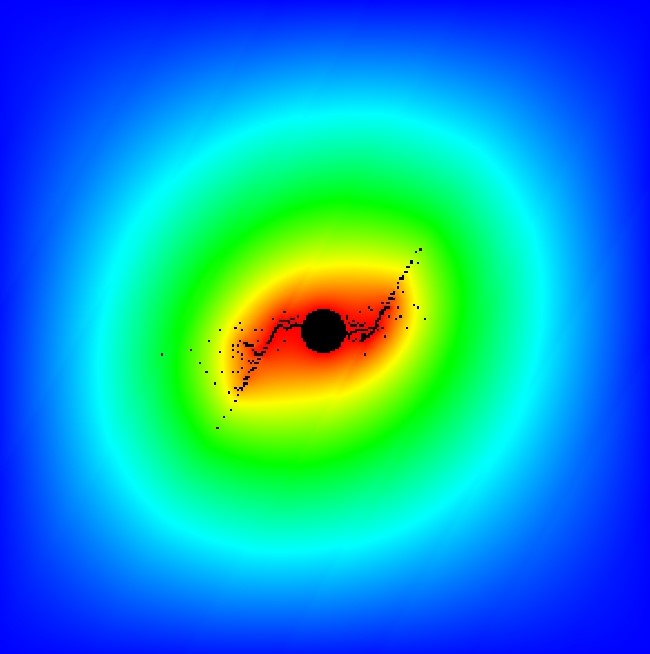

Supplement: S2 Fig — (ZIP) [file pone.0294993.s002.zip › S2_Fig/60°/△σ=2MPa/0022-0010.jpg]

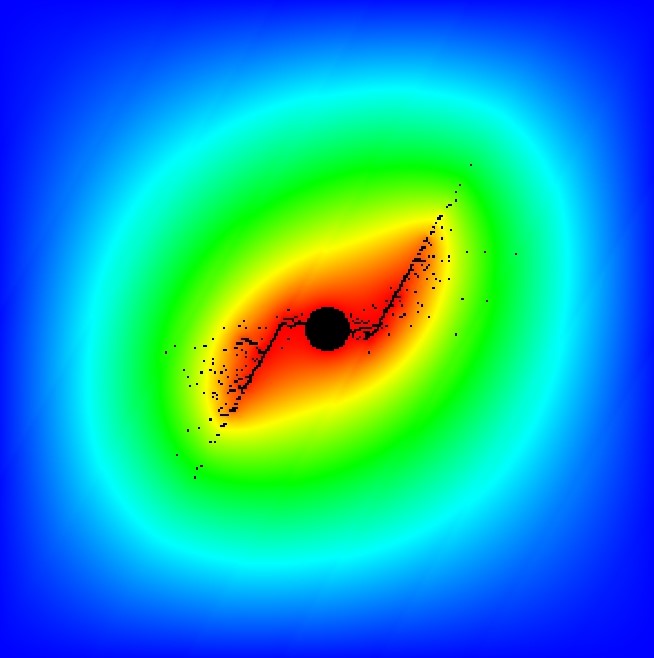

Supplement: S2 Fig — (ZIP) [file pone.0294993.s002.zip › S2_Fig/60°/△σ=2MPa/0022-0012.jpg]

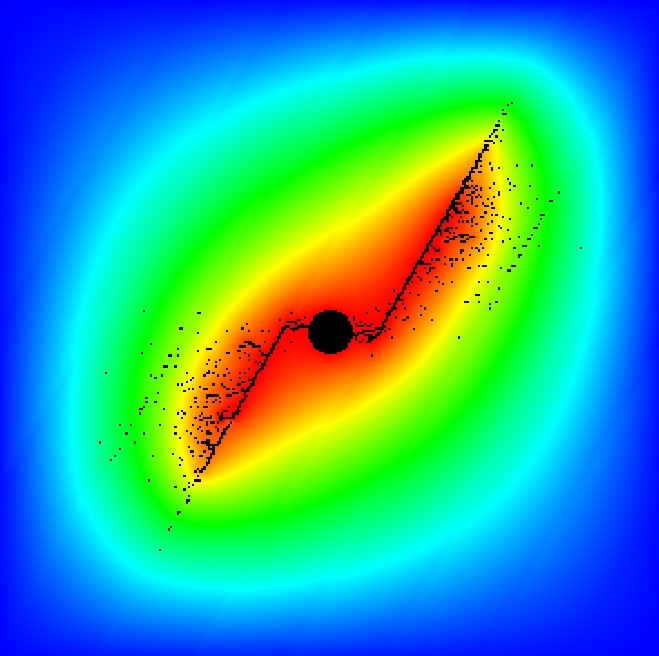

Supplement: S2 Fig — (ZIP) [file pone.0294993.s002.zip › S2_Fig/60°/△σ=2MPa/0022-0014.jpg]

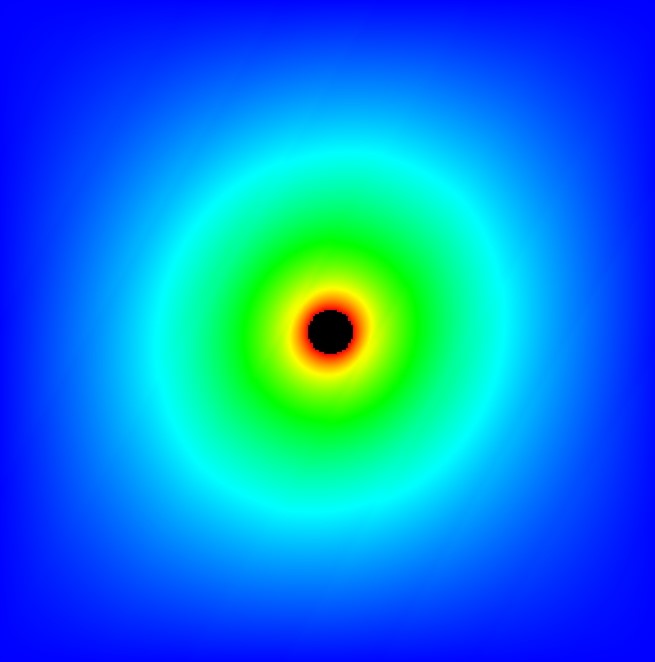

Supplement: S2 Fig — (ZIP) [file pone.0294993.s002.zip › S2_Fig/60°/△σ=4MPa/0001-0001.jpg]

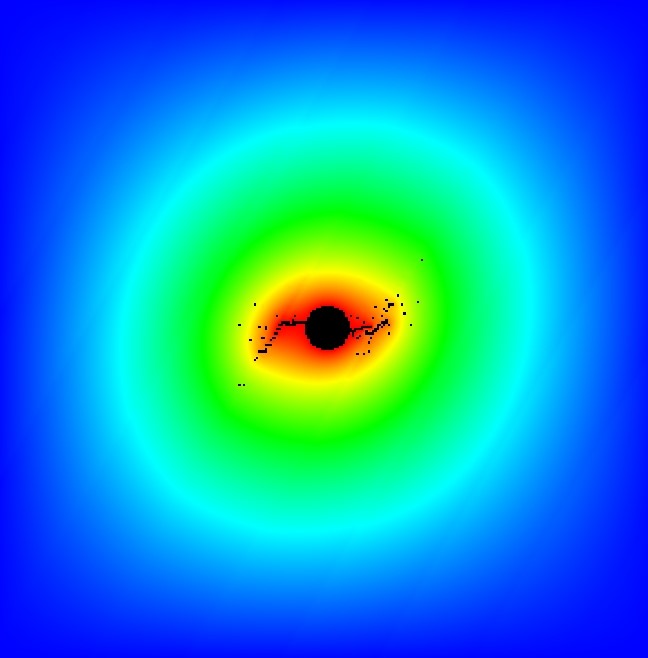

Supplement: S2 Fig — (ZIP) [file pone.0294993.s002.zip › S2_Fig/60°/△σ=4MPa/0032-0002.jpg]

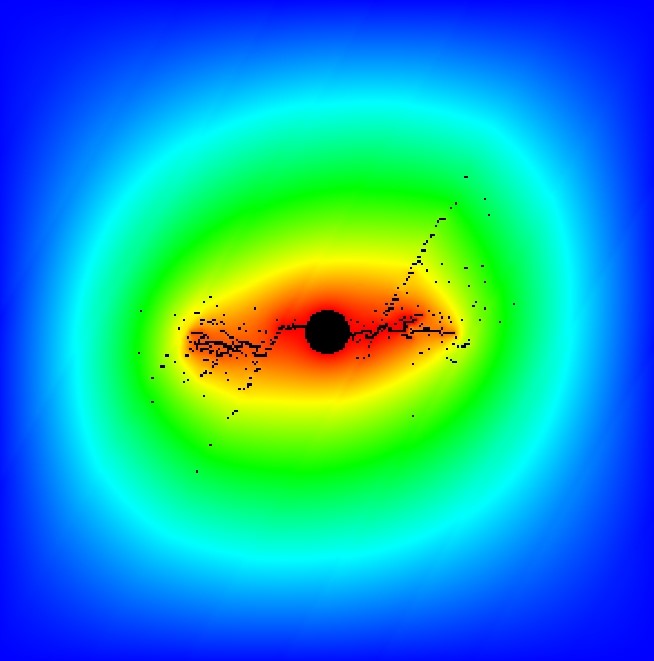

Supplement: S2 Fig — (ZIP) [file pone.0294993.s002.zip › S2_Fig/60°/△σ=4MPa/0044-0001.jpg]

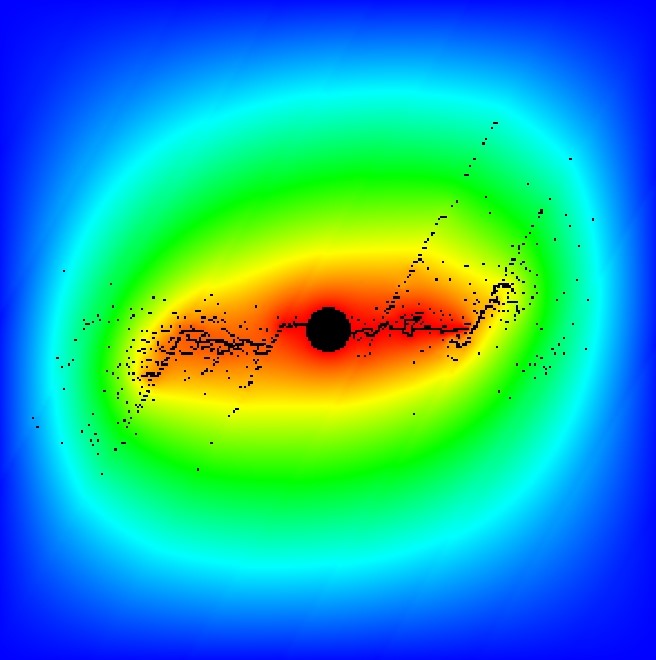

Supplement: S2 Fig — (ZIP) [file pone.0294993.s002.zip › S2_Fig/60°/△σ=4MPa/0046-0002.jpg]

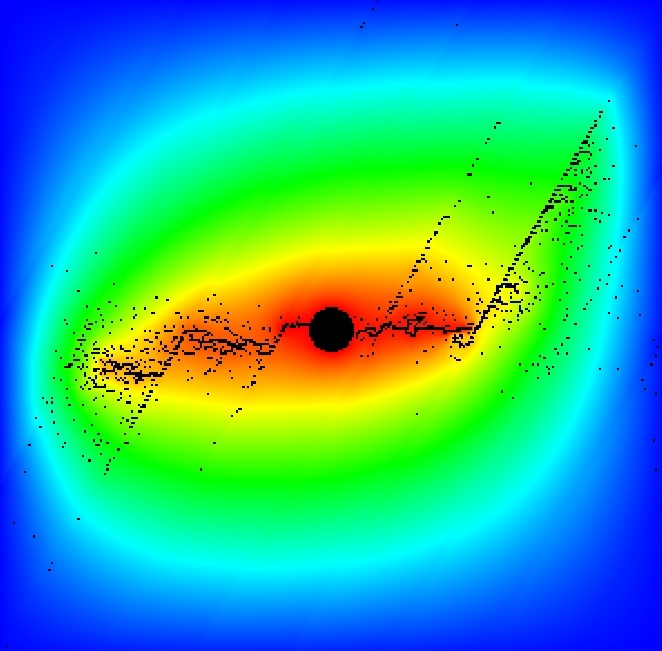

Supplement: S2 Fig — (ZIP) [file pone.0294993.s002.zip › S2_Fig/60°/△σ=4MPa/0046-0009.jpg]

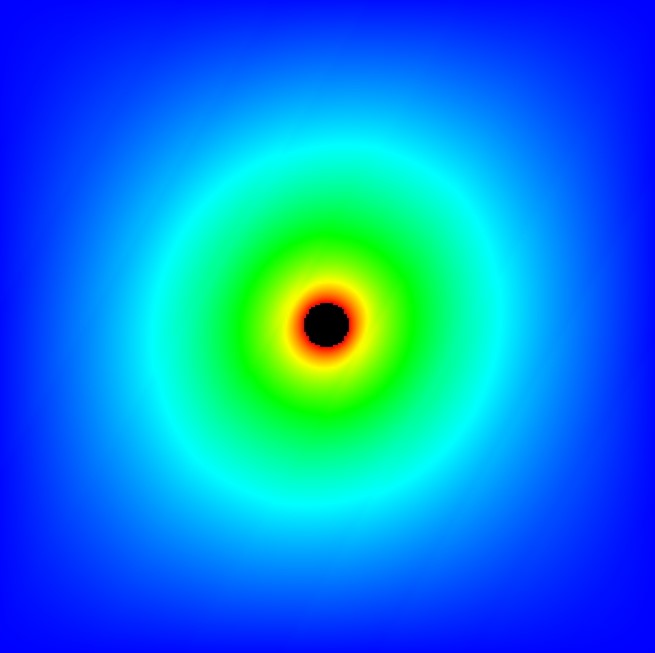

Supplement: S2 Fig — (ZIP) [file pone.0294993.s002.zip › S2_Fig/60°/△σ=6MPa/0001-0001.jpg]

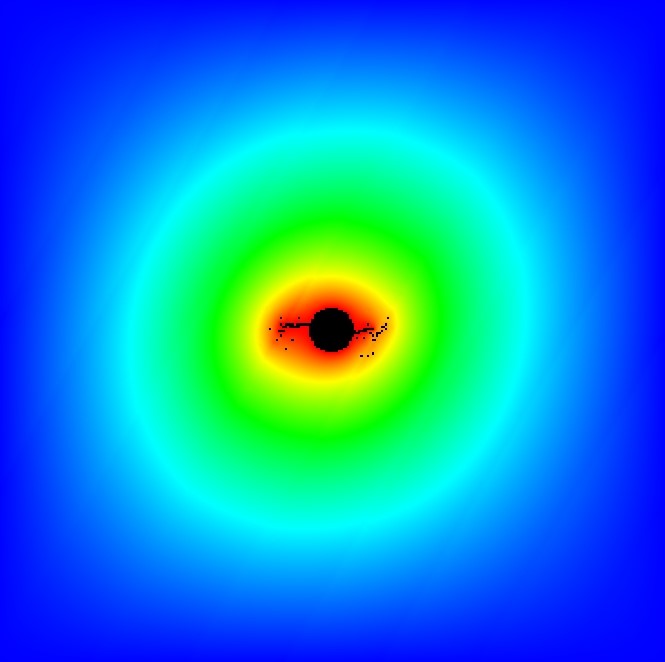

Supplement: S2 Fig — (ZIP) [file pone.0294993.s002.zip › S2_Fig/60°/△σ=6MPa/0072-0002.jpg]

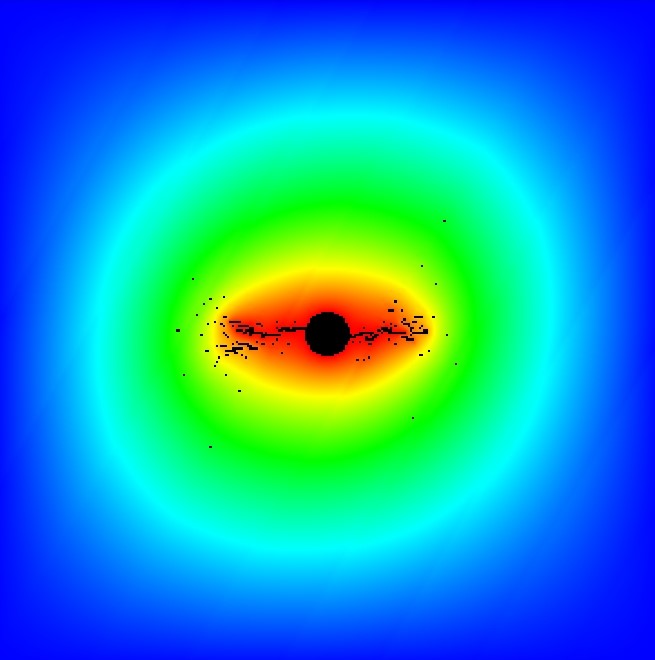

Supplement: S2 Fig — (ZIP) [file pone.0294993.s002.zip › S2_Fig/60°/△σ=6MPa/0084-0002.jpg]

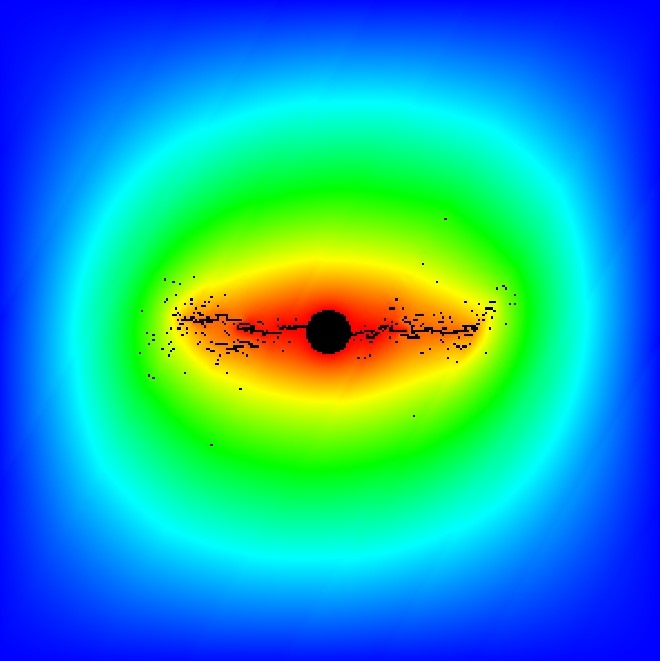

Supplement: S2 Fig — (ZIP) [file pone.0294993.s002.zip › S2_Fig/60°/△σ=6MPa/0089-0004.jpg]

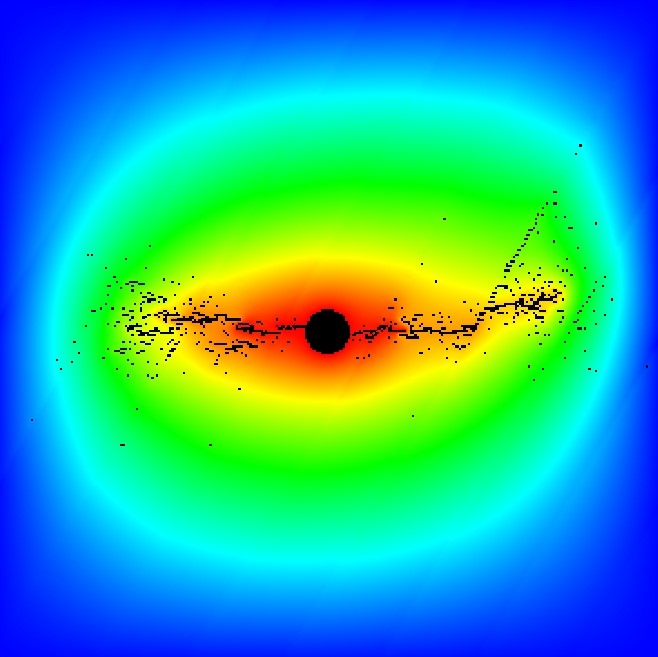

Supplement: S2 Fig — (ZIP) [file pone.0294993.s002.zip › S2_Fig/60°/△σ=6MPa/0090-0007.jpg]

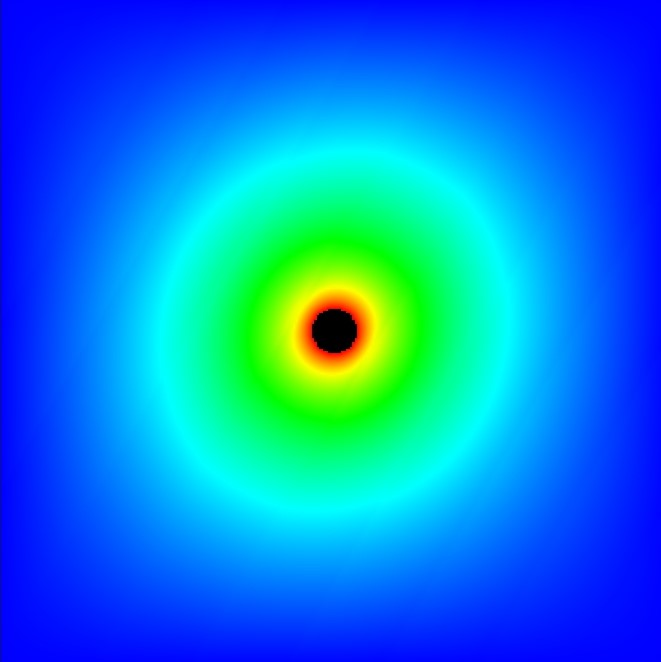

Supplement: S2 Fig — (ZIP) [file pone.0294993.s002.zip › S2_Fig/60°/△σ=8MPa/0001-0001.jpg]

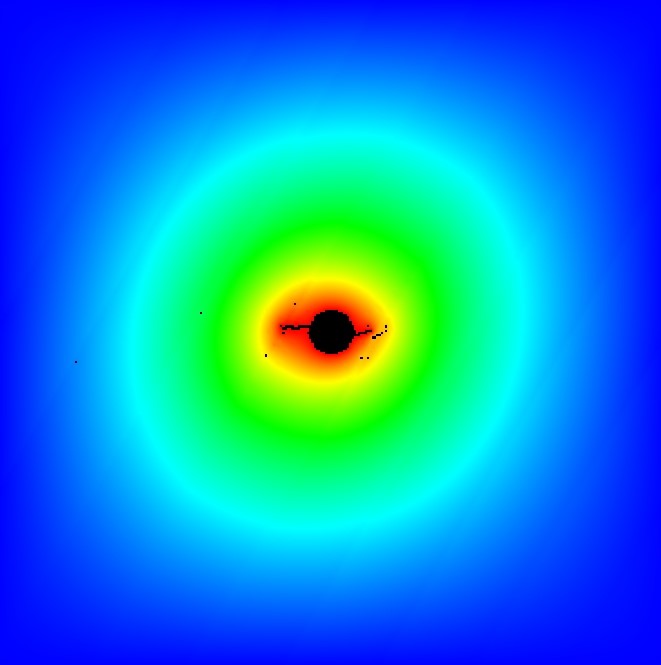

Supplement: S2 Fig — (ZIP) [file pone.0294993.s002.zip › S2_Fig/60°/△σ=8MPa/0045-0001.jpg]

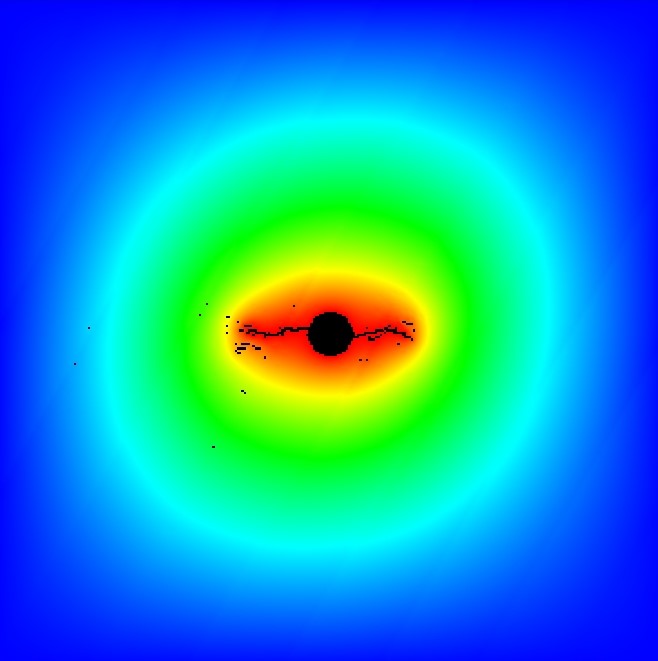

Supplement: S2 Fig — (ZIP) [file pone.0294993.s002.zip › S2_Fig/60°/△σ=8MPa/0048-0008.jpg]

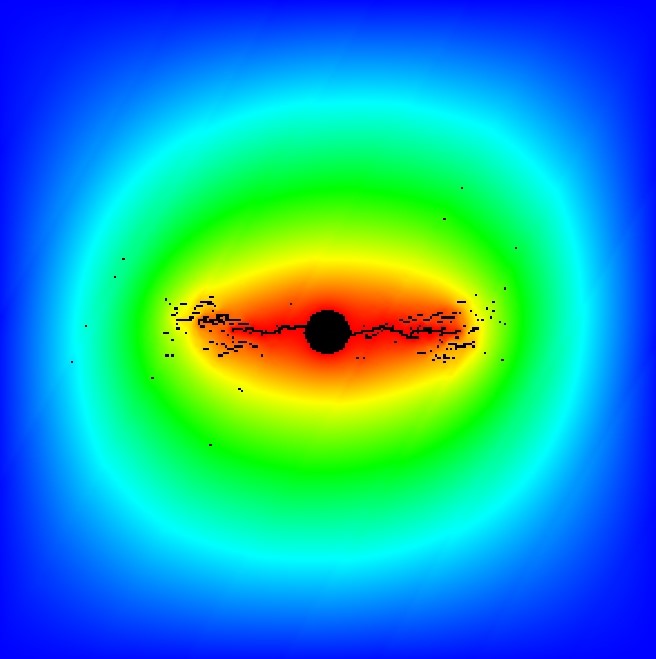

Supplement: S2 Fig — (ZIP) [file pone.0294993.s002.zip › S2_Fig/60°/△σ=8MPa/0048-0015.jpg]

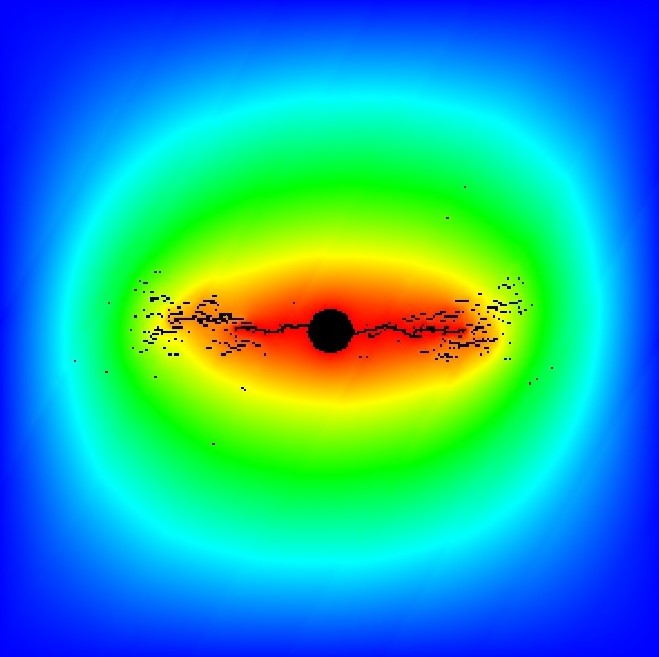

Supplement: S2 Fig — (ZIP) [file pone.0294993.s002.zip › S2_Fig/60°/△σ=8MPa/0048-0019.jpg]

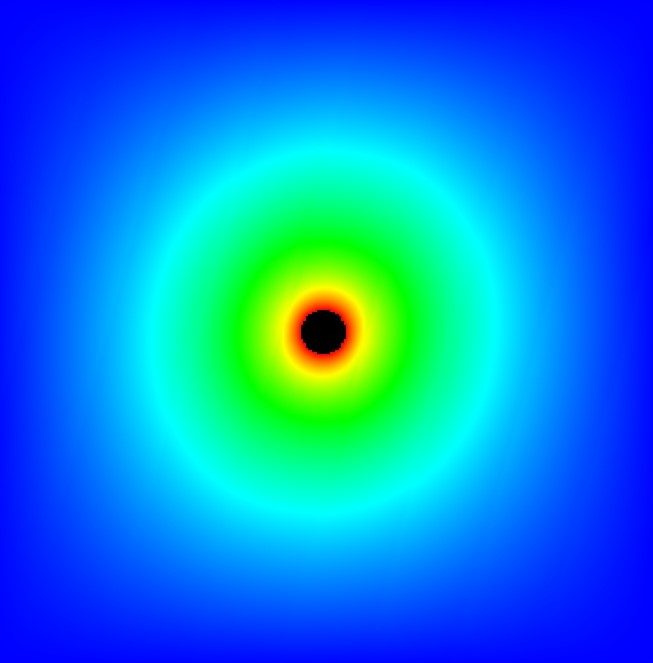

Supplement: S2 Fig — (ZIP) [file pone.0294993.s002.zip › S2_Fig/75°/△σ=2MPa/0001-0001.jpg]

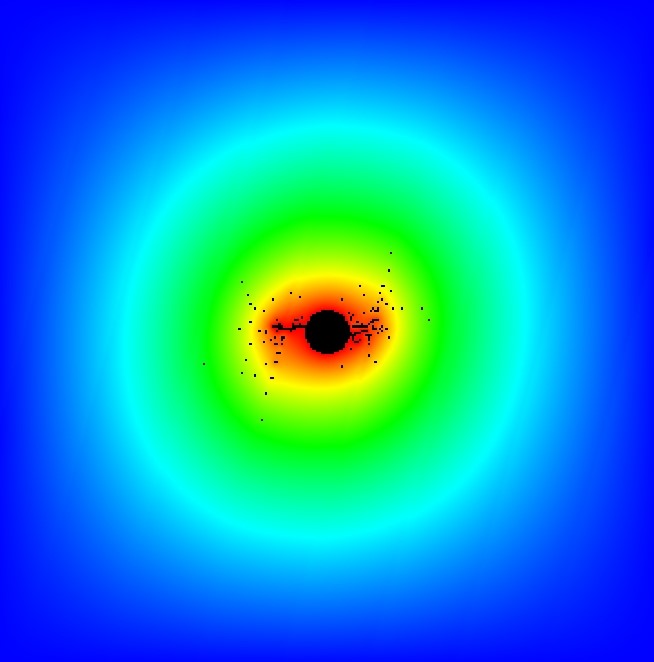

Supplement: S2 Fig — (ZIP) [file pone.0294993.s002.zip › S2_Fig/75°/△σ=2MPa/0047-0002.jpg]

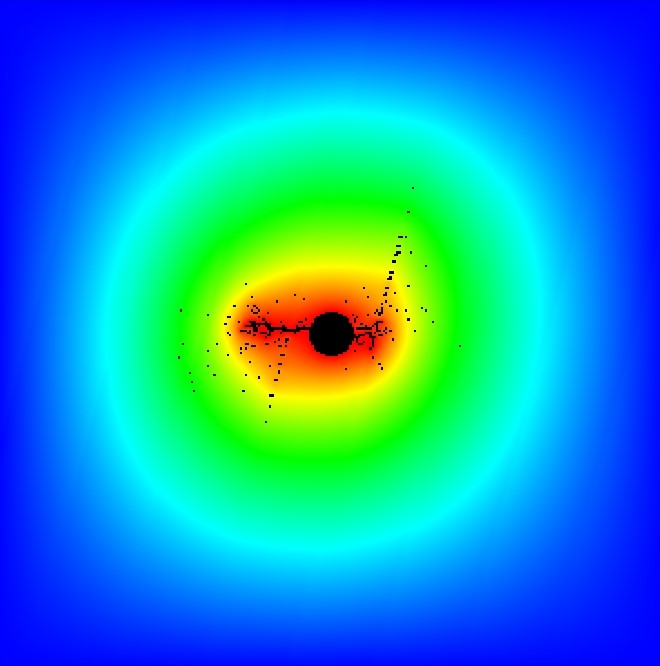

Supplement: S2 Fig — (ZIP) [file pone.0294993.s002.zip › S2_Fig/75°/△σ=2MPa/0054-0001.jpg]

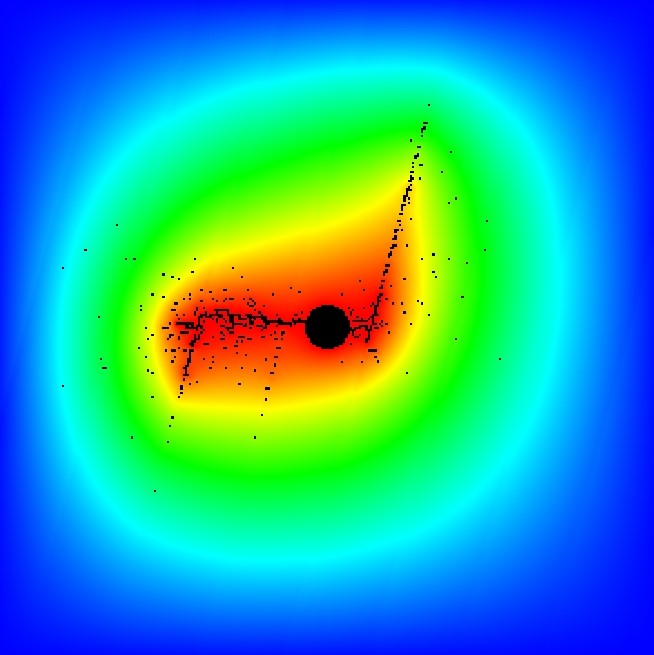

Supplement: S2 Fig — (ZIP) [file pone.0294993.s002.zip › S2_Fig/75°/△σ=2MPa/0059-0001.jpg]

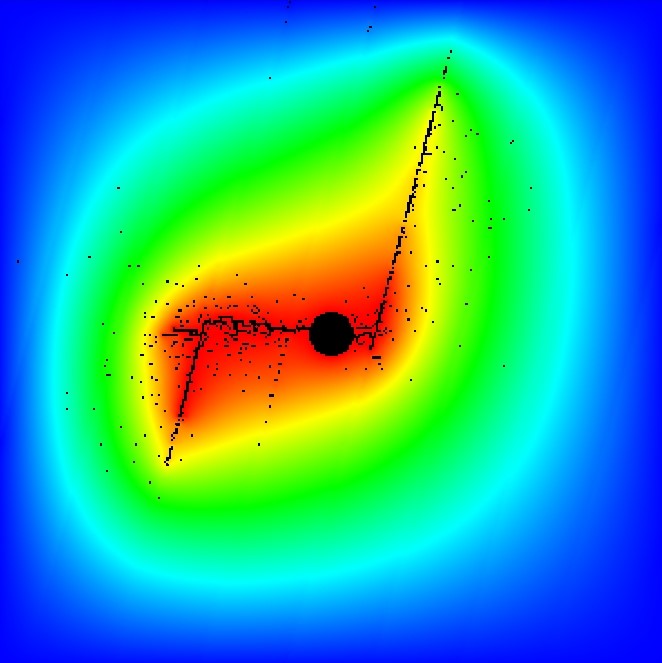

Supplement: S2 Fig — (ZIP) [file pone.0294993.s002.zip › S2_Fig/75°/△σ=2MPa/0059-0004.jpg]

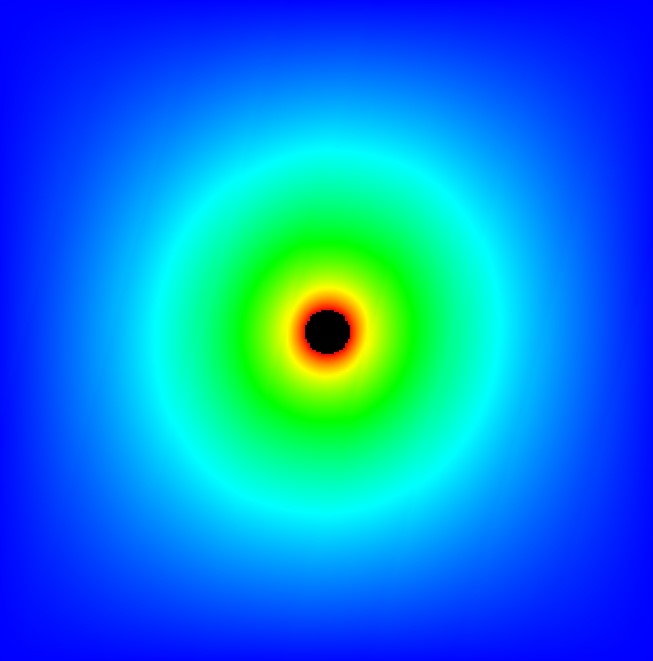

Supplement: S2 Fig — (ZIP) [file pone.0294993.s002.zip › S2_Fig/75°/△σ=4MPa/0001-0001.jpg]

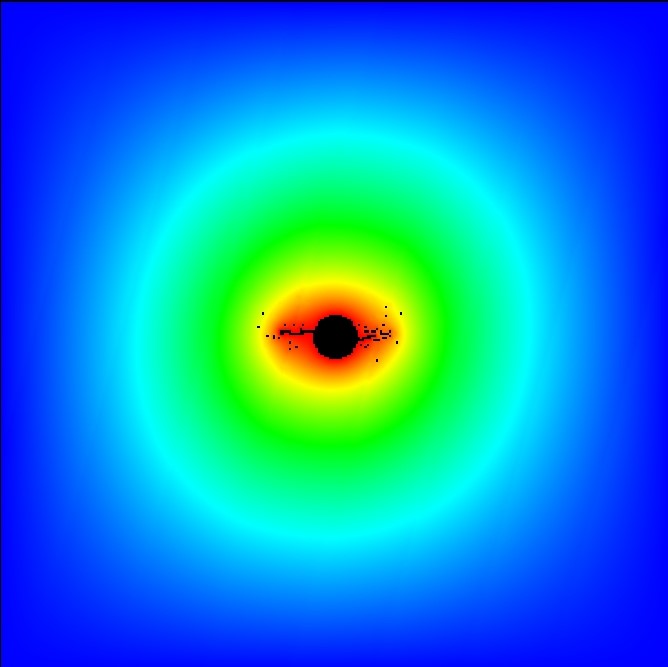

Supplement: S2 Fig — (ZIP) [file pone.0294993.s002.zip › S2_Fig/75°/△σ=4MPa/0042-0001.jpg]

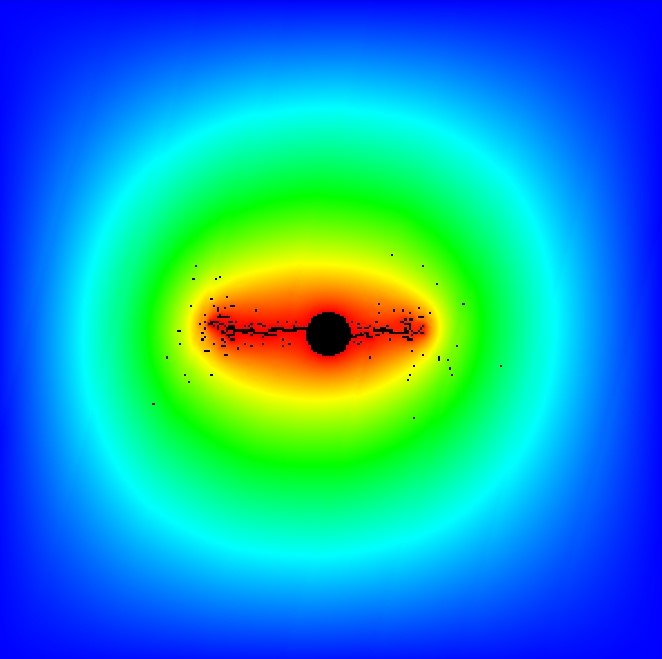

Supplement: S2 Fig — (ZIP) [file pone.0294993.s002.zip › S2_Fig/75°/△σ=4MPa/0053-0001.jpg]

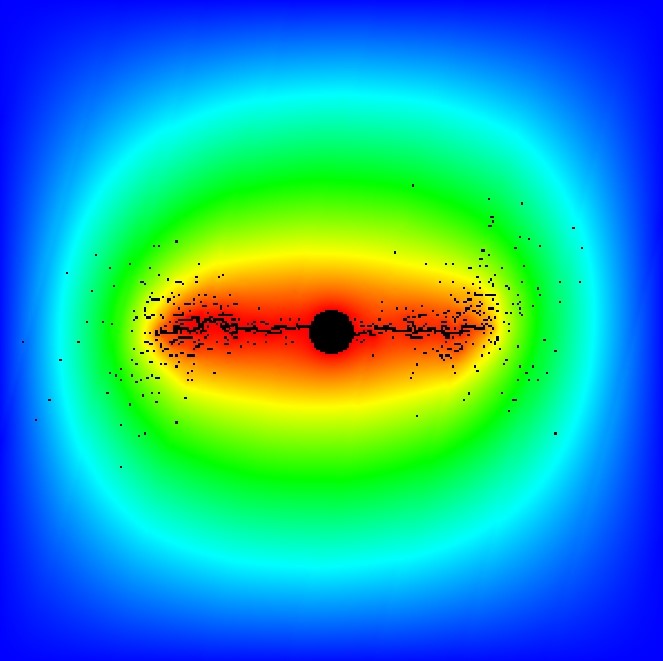

Supplement: S2 Fig — (ZIP) [file pone.0294993.s002.zip › S2_Fig/75°/△σ=4MPa/0057-0002.jpg]

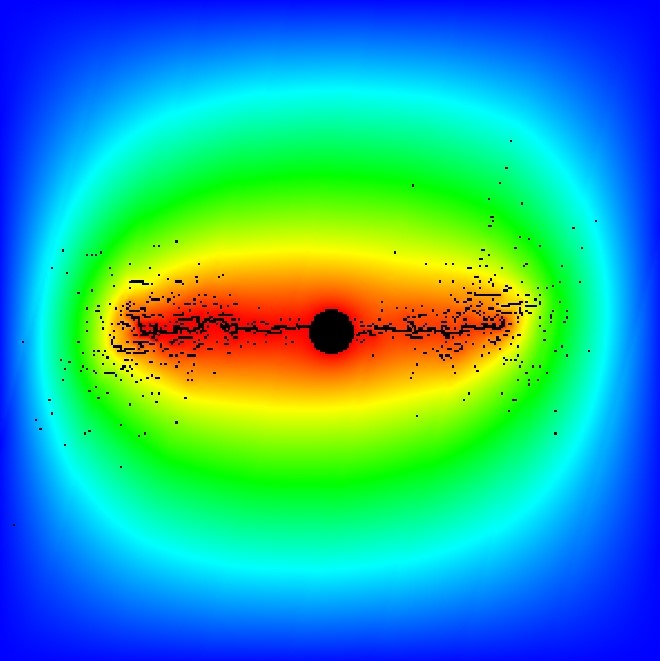

Supplement: S2 Fig — (ZIP) [file pone.0294993.s002.zip › S2_Fig/75°/△σ=4MPa/0057-0008.jpg]

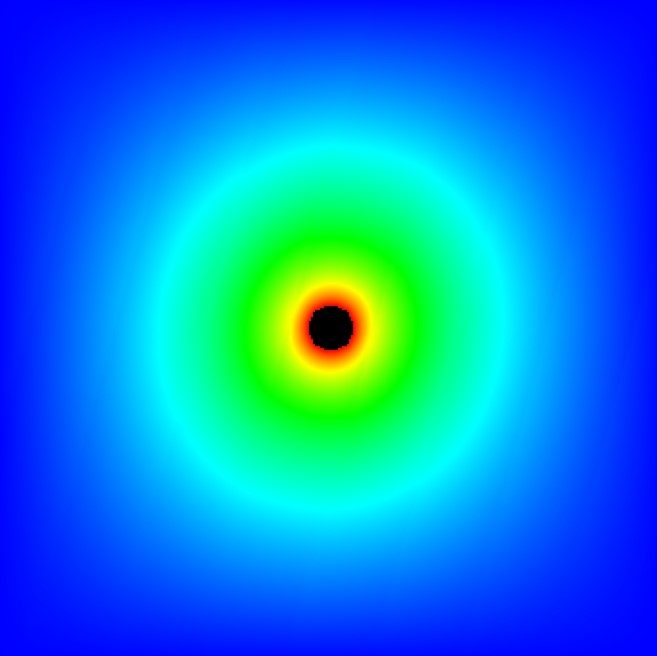

Supplement: S2 Fig — (ZIP) [file pone.0294993.s002.zip › S2_Fig/75°/△σ=6MPa/0001-0001.jpg]

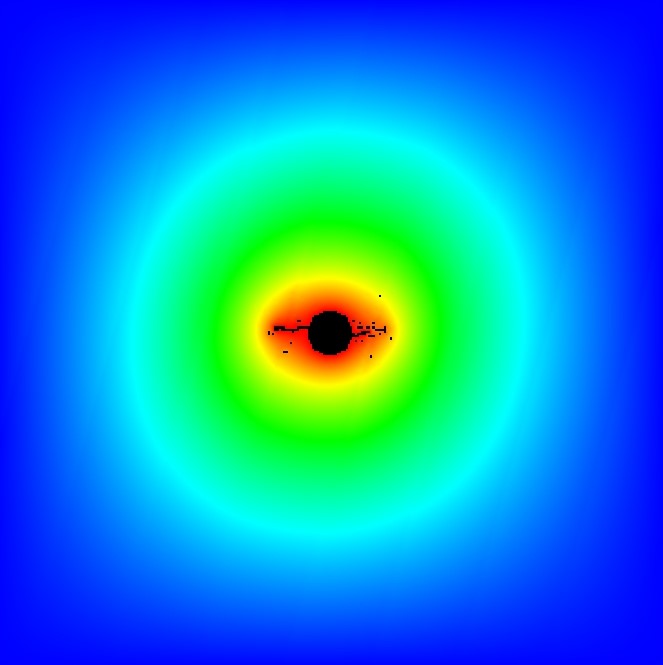

Supplement: S2 Fig — (ZIP) [file pone.0294993.s002.zip › S2_Fig/75°/△σ=6MPa/0027-0001.jpg]

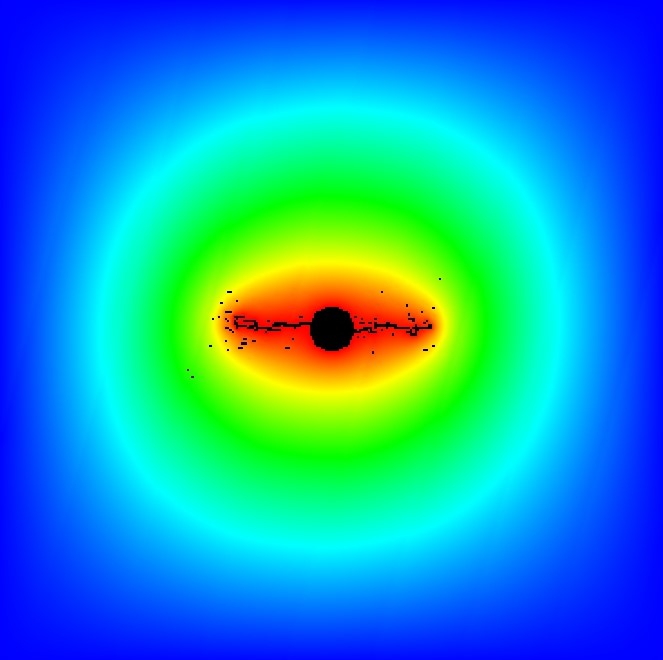

Supplement: S2 Fig — (ZIP) [file pone.0294993.s002.zip › S2_Fig/75°/△σ=6MPa/0036-0002.jpg]

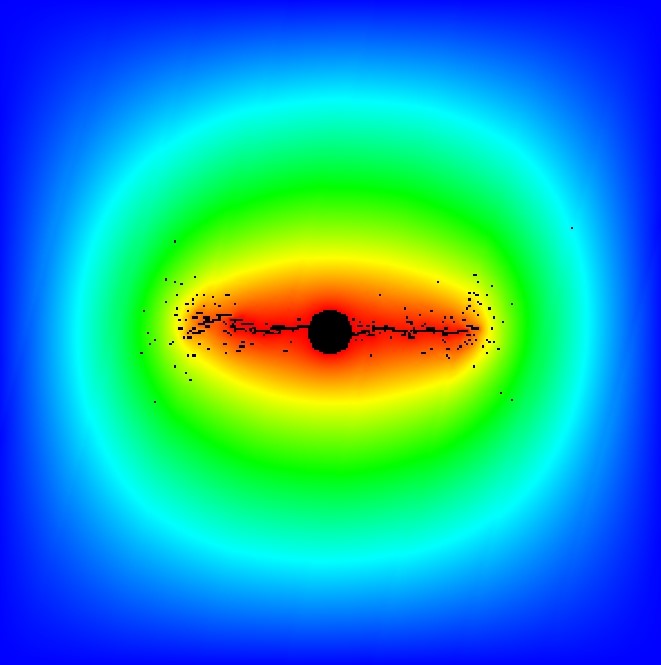

Supplement: S2 Fig — (ZIP) [file pone.0294993.s002.zip › S2_Fig/75°/△σ=6MPa/0041-0001.jpg]

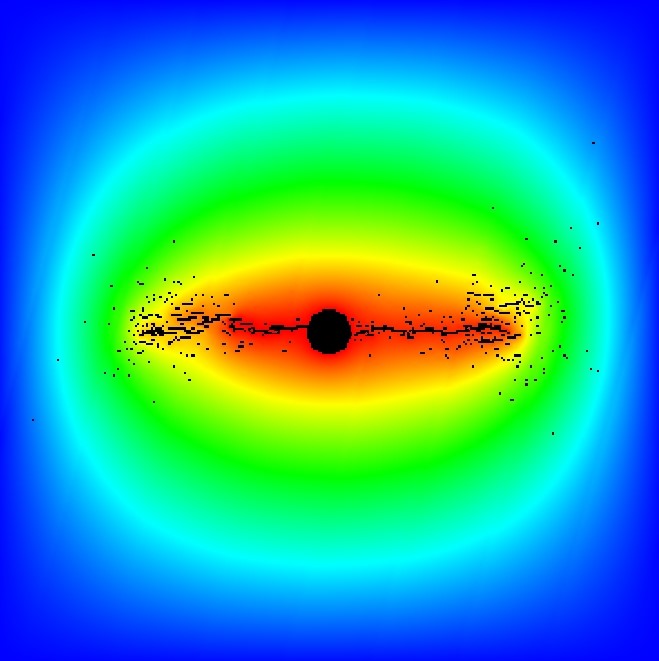

Supplement: S2 Fig — (ZIP) [file pone.0294993.s002.zip › S2_Fig/75°/△σ=6MPa/0042-0005.jpg]

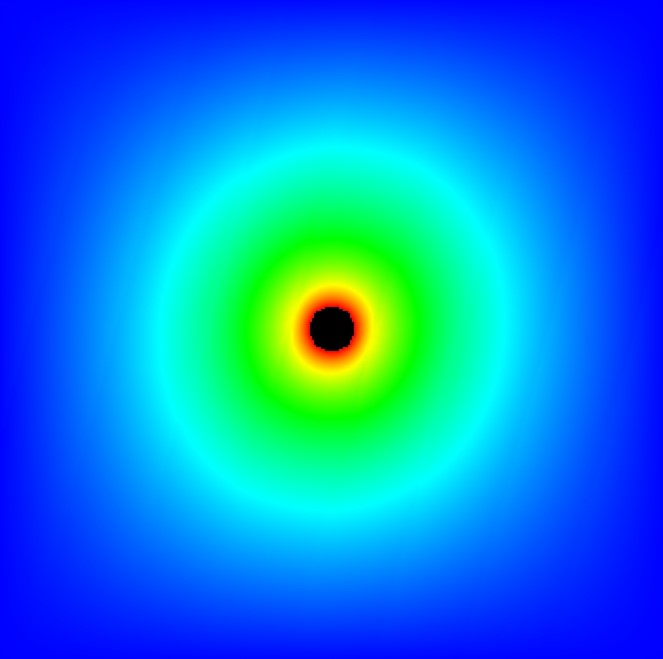

Supplement: S2 Fig — (ZIP) [file pone.0294993.s002.zip › S2_Fig/75°/△σ=8MPa/0001-0001.jpg]

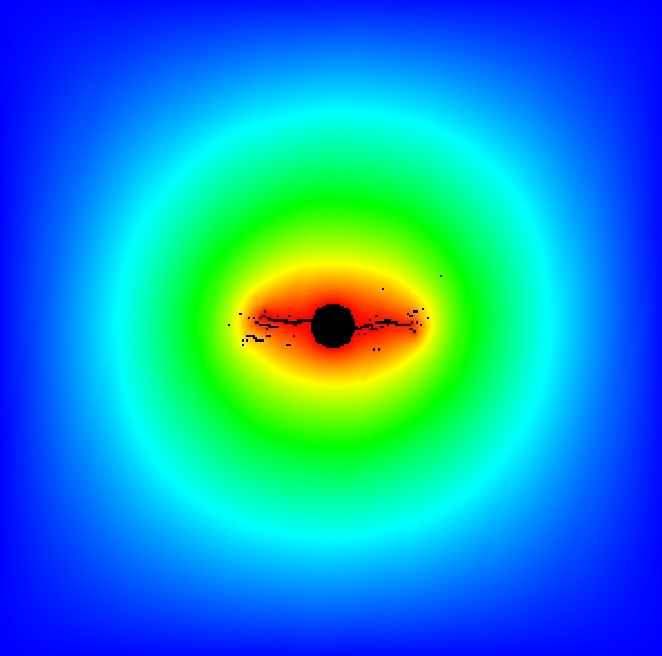

Supplement: S2 Fig — (ZIP) [file pone.0294993.s002.zip › S2_Fig/75°/△σ=8MPa/0038-0001.jpg]

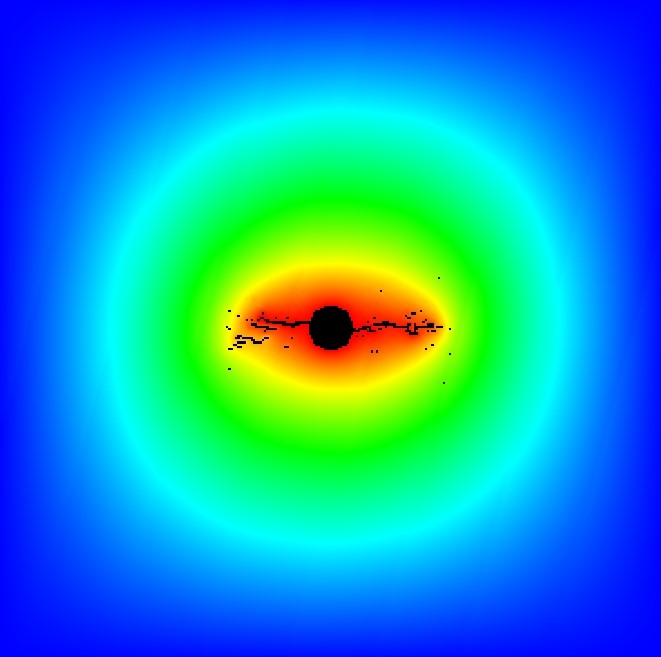

Supplement: S2 Fig — (ZIP) [file pone.0294993.s002.zip › S2_Fig/75°/△σ=8MPa/0040-0003.jpg]

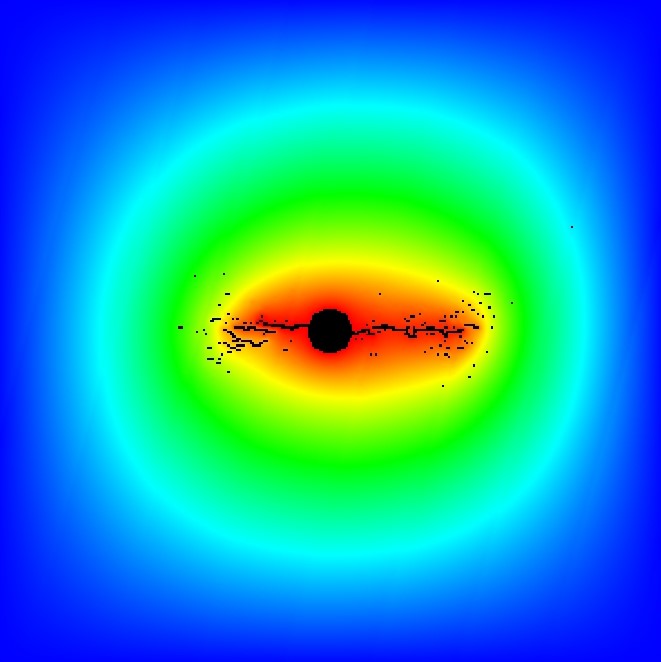

Supplement: S2 Fig — (ZIP) [file pone.0294993.s002.zip › S2_Fig/75°/△σ=8MPa/0042-0002.jpg]

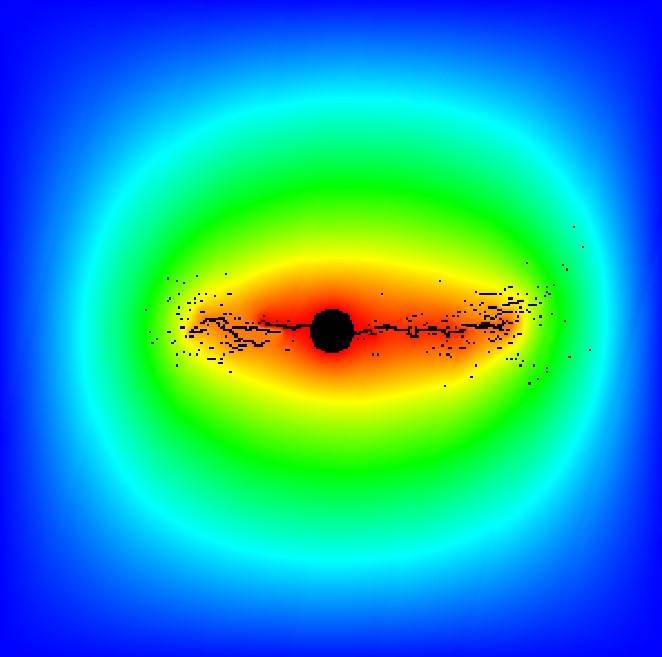

Supplement: S2 Fig — (ZIP) [file pone.0294993.s002.zip › S2_Fig/75°/△σ=8MPa/0043-0007.jpg]
