# Supplementary figures and images for: Prediction criterion and numerical validation for the interaction between hydraulic fractures and bedding planes (part 2 of 2)
Source: PLoS One. 2023 Dec 21;18(12):e0294993. doi: 10.1371/journal.pone.0294993 (PMC10735180; doi:10.1371/journal.pone.0294993)

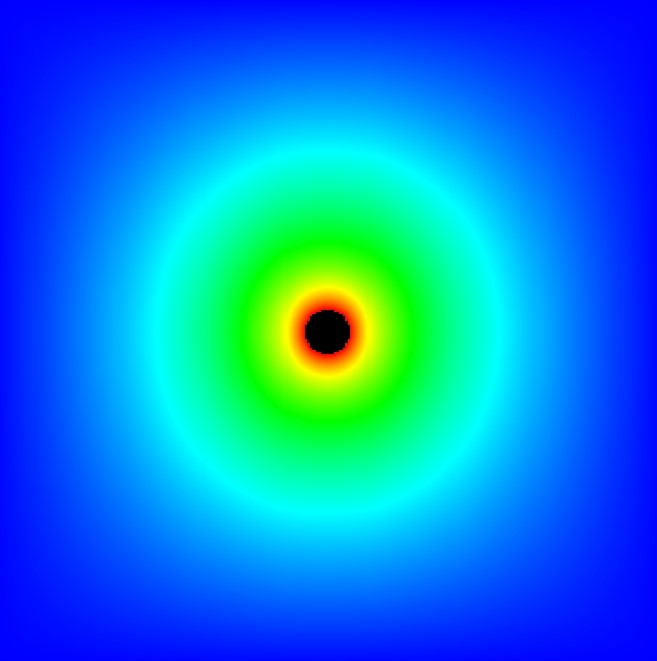

Supplement: S2 Fig — (ZIP) [file pone.0294993.s002.zip › S2_Fig/90°/△σ=2MPa/0001-0001.jpg]

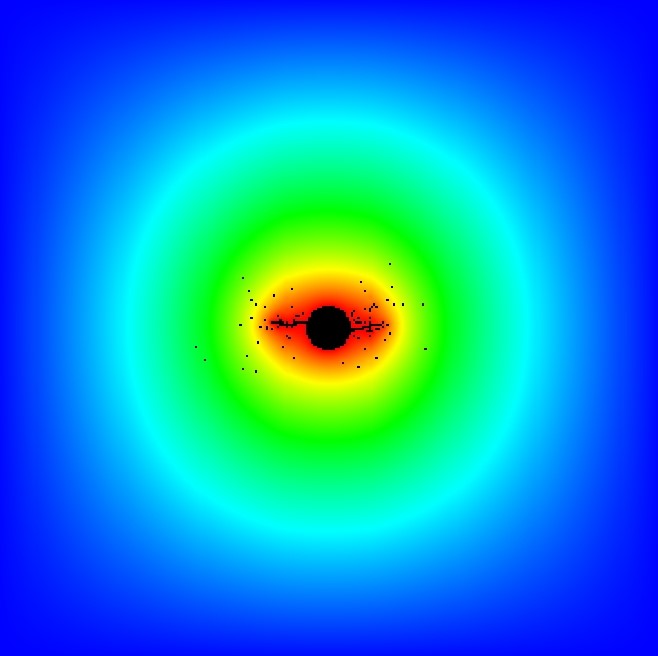

Supplement: S2 Fig — (ZIP) [file pone.0294993.s002.zip › S2_Fig/90°/△σ=2MPa/0037-0002.jpg]

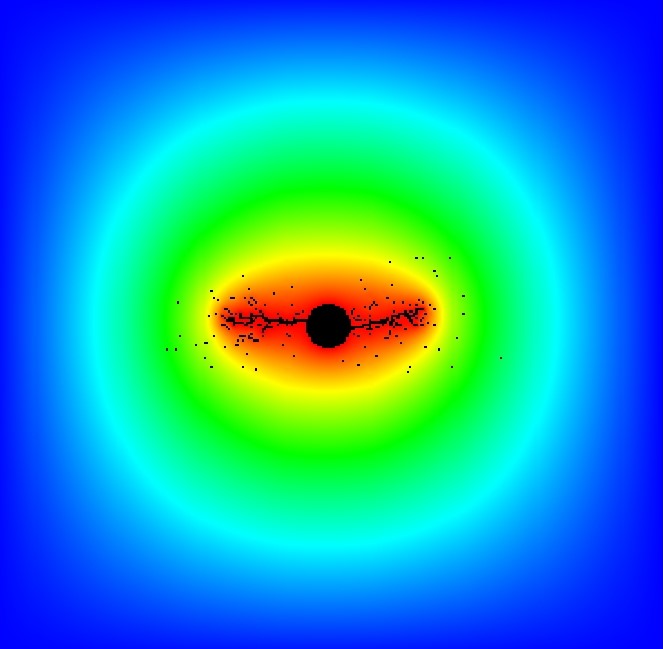

Supplement: S2 Fig — (ZIP) [file pone.0294993.s002.zip › S2_Fig/90°/△σ=2MPa/0043-0003.jpg]

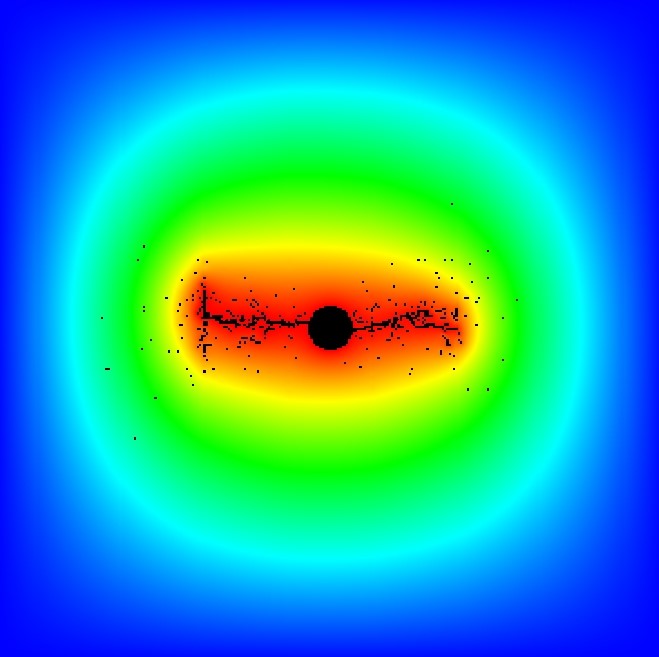

Supplement: S2 Fig — (ZIP) [file pone.0294993.s002.zip › S2_Fig/90°/△σ=2MPa/0048-0001.jpg]

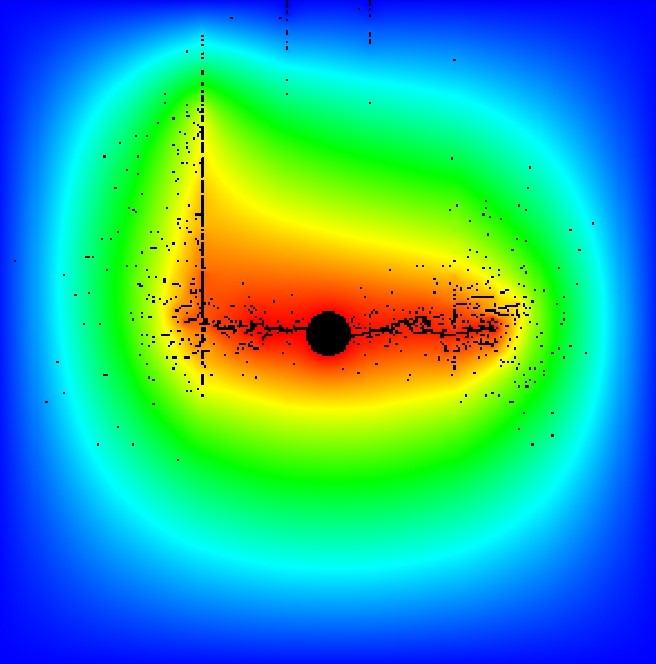

Supplement: S2 Fig — (ZIP) [file pone.0294993.s002.zip › S2_Fig/90°/△σ=2MPa/0052-0002.jpg]

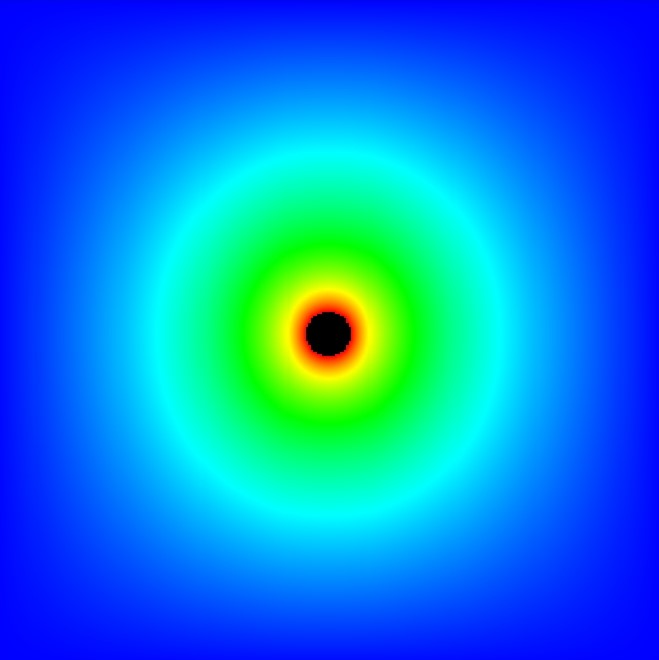

Supplement: S2 Fig — (ZIP) [file pone.0294993.s002.zip › S2_Fig/90°/△σ=4MPa/0001-0001.jpg]

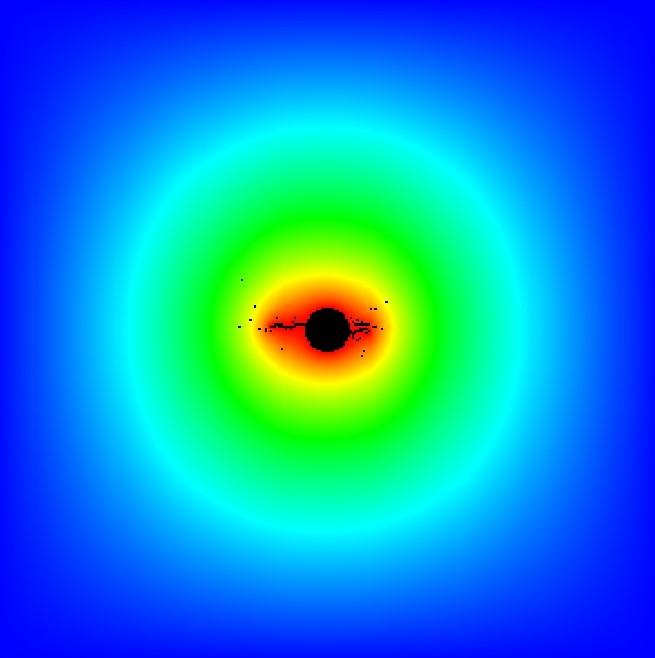

Supplement: S2 Fig — (ZIP) [file pone.0294993.s002.zip › S2_Fig/90°/△σ=4MPa/0037-0001.jpg]

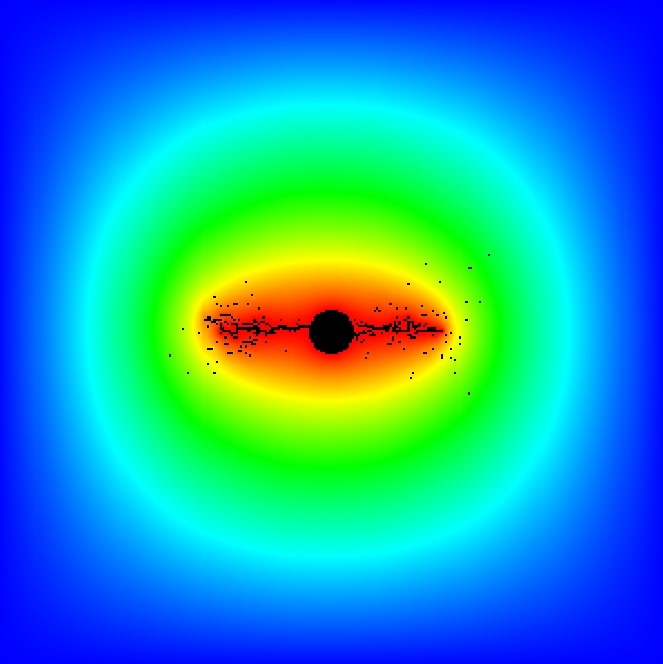

Supplement: S2 Fig — (ZIP) [file pone.0294993.s002.zip › S2_Fig/90°/△σ=4MPa/0043-0005.jpg]

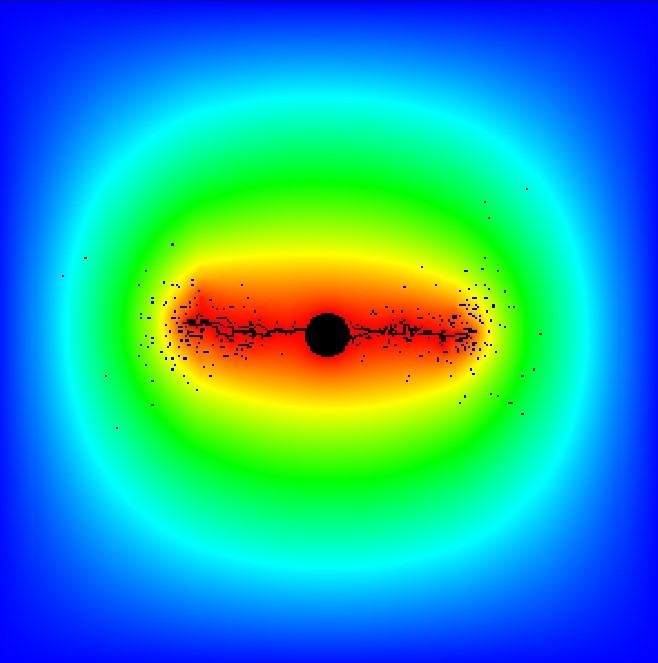

Supplement: S2 Fig — (ZIP) [file pone.0294993.s002.zip › S2_Fig/90°/△σ=4MPa/0045-0002.jpg]

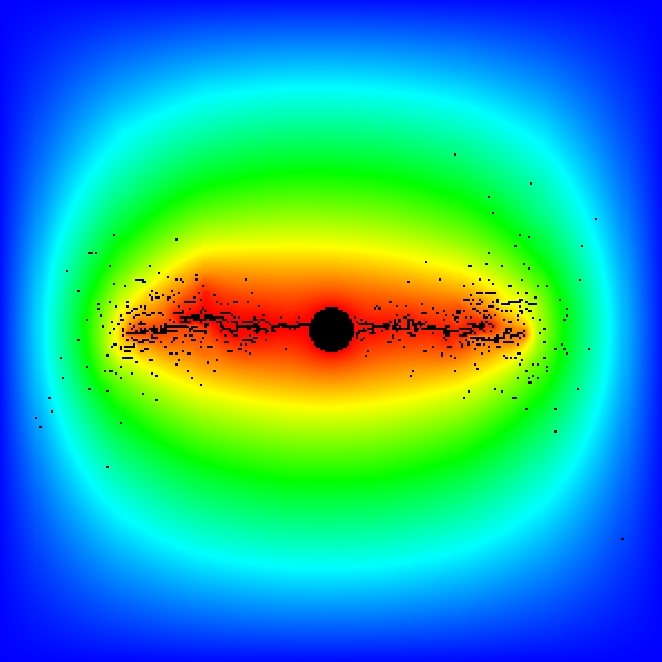

Supplement: S2 Fig — (ZIP) [file pone.0294993.s002.zip › S2_Fig/90°/△σ=4MPa/0047-0009.jpg]

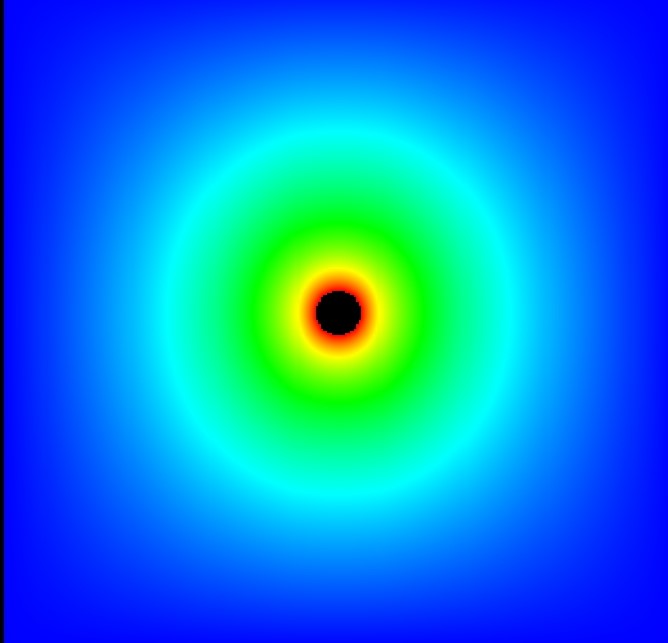

Supplement: S2 Fig — (ZIP) [file pone.0294993.s002.zip › S2_Fig/90°/△σ=6MPa/0001-0001.jpg]

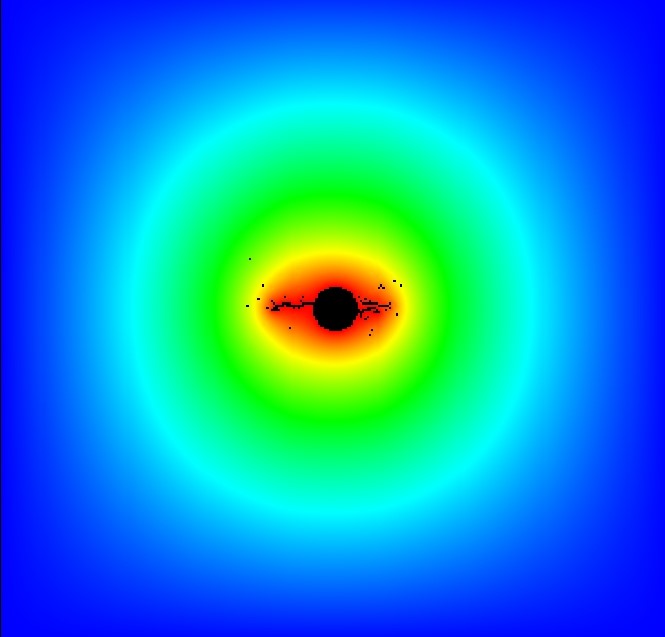

Supplement: S2 Fig — (ZIP) [file pone.0294993.s002.zip › S2_Fig/90°/△σ=6MPa/0037-0002.jpg]

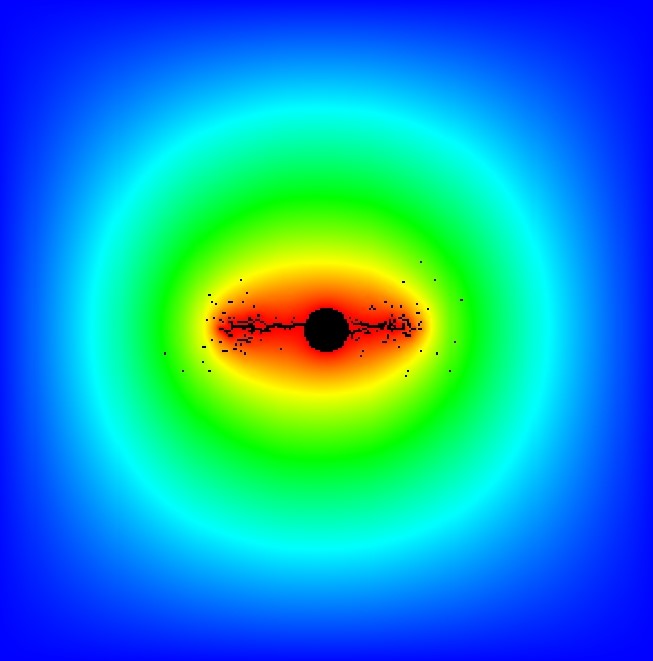

Supplement: S2 Fig — (ZIP) [file pone.0294993.s002.zip › S2_Fig/90°/△σ=6MPa/0043-0001.jpg]

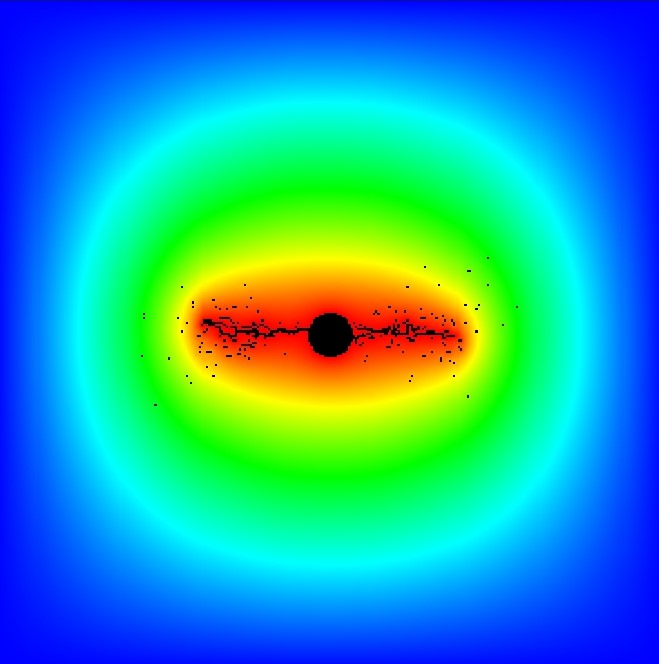

Supplement: S2 Fig — (ZIP) [file pone.0294993.s002.zip › S2_Fig/90°/△σ=6MPa/0044-0001.jpg]

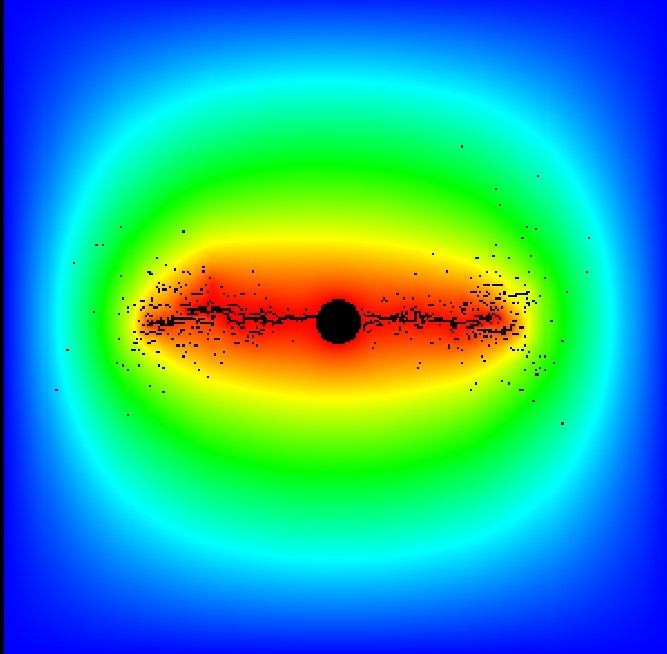

Supplement: S2 Fig — (ZIP) [file pone.0294993.s002.zip › S2_Fig/90°/△σ=6MPa/0047-0005.jpg]

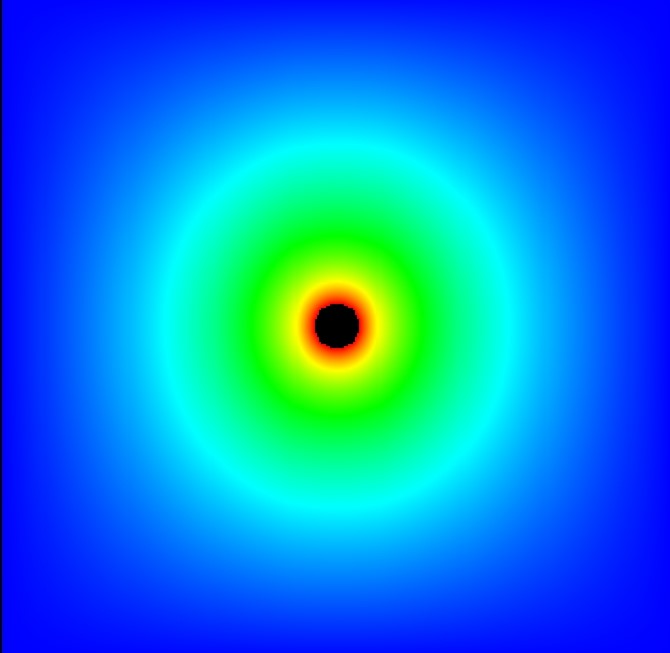

Supplement: S2 Fig — (ZIP) [file pone.0294993.s002.zip › S2_Fig/90°/△σ=8MPa/0001-0001.jpg]

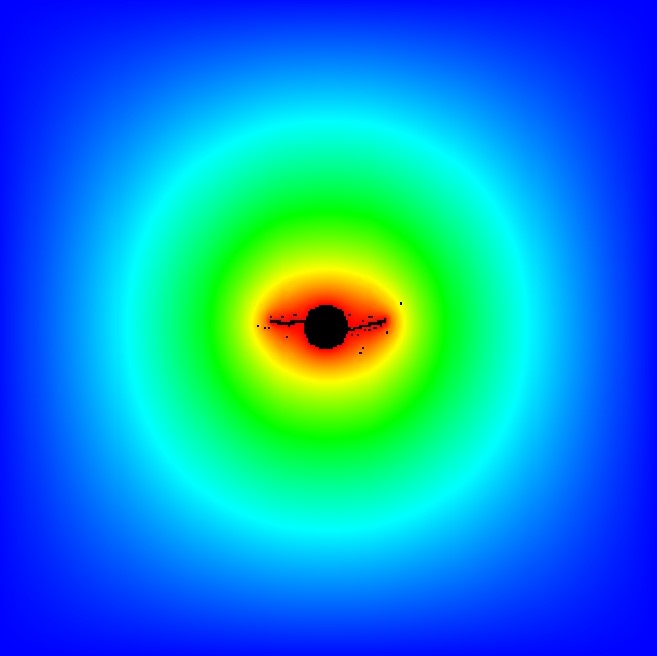

Supplement: S2 Fig — (ZIP) [file pone.0294993.s002.zip › S2_Fig/90°/△σ=8MPa/0029-0001.jpg]

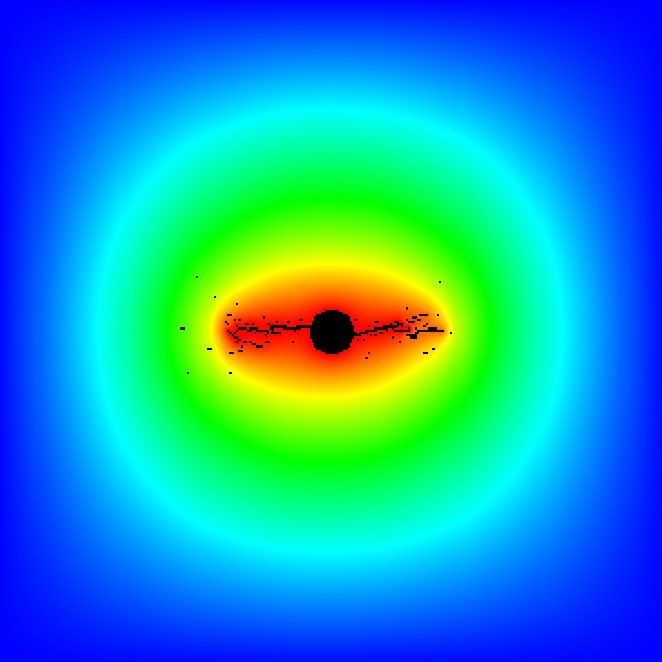

Supplement: S2 Fig — (ZIP) [file pone.0294993.s002.zip › S2_Fig/90°/△σ=8MPa/0039-0001.jpg]

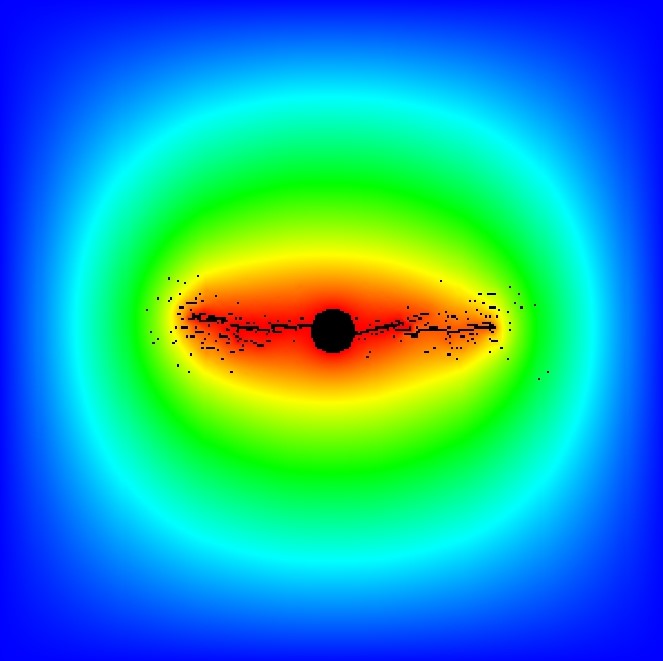

Supplement: S2 Fig — (ZIP) [file pone.0294993.s002.zip › S2_Fig/90°/△σ=8MPa/0041-0002.jpg]

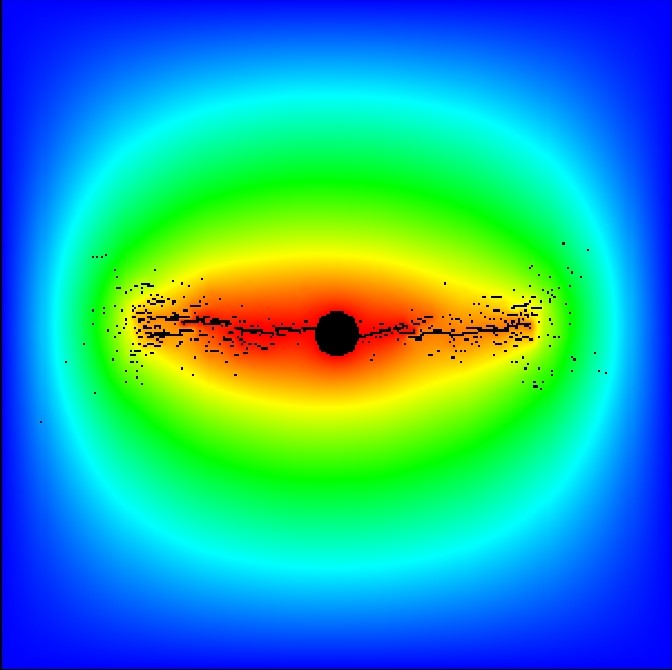

Supplement: S2 Fig — (ZIP) [file pone.0294993.s002.zip › S2_Fig/90°/△σ=8MPa/0041-0011.jpg]

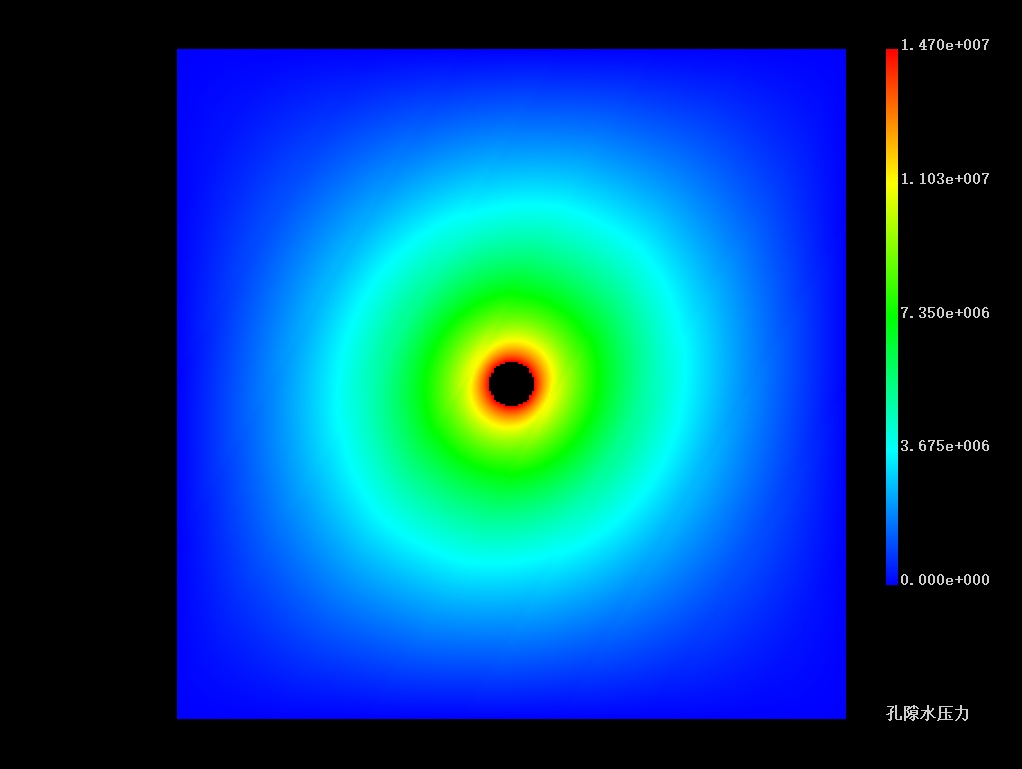

Supplement: S3 Fig — (ZIP) [file pone.0294993.s003.zip › S3_Fig/σ1=10MPa σ3=8MPa/0001-0001.jpg]

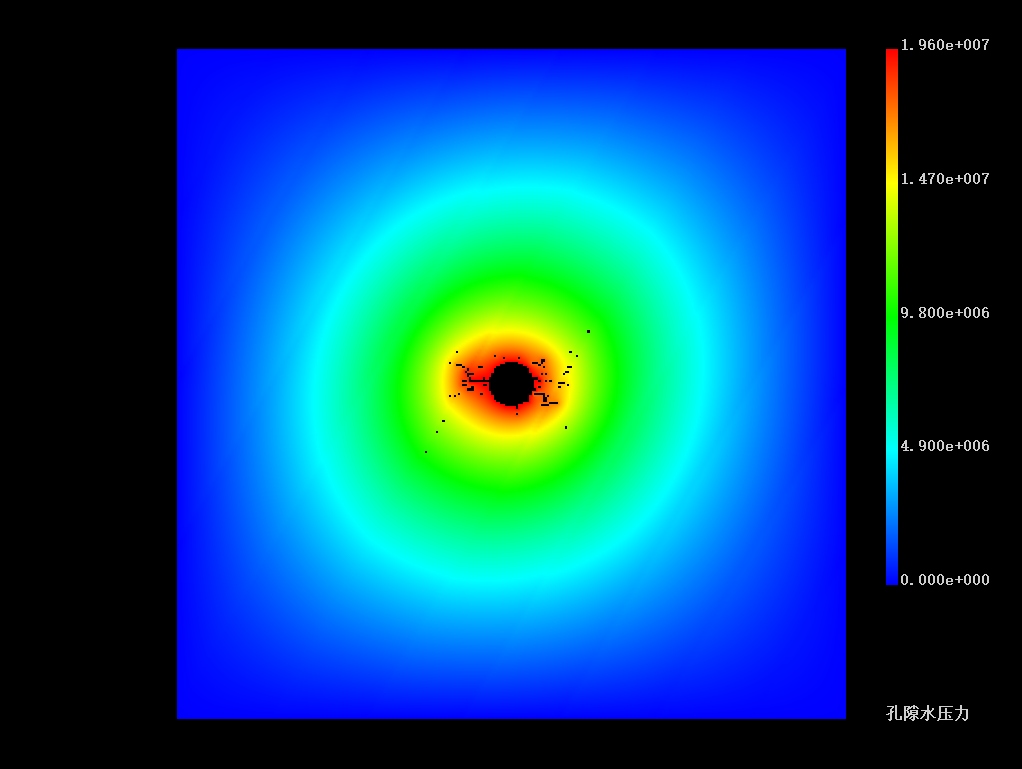

Supplement: S3 Fig — (ZIP) [file pone.0294993.s003.zip › S3_Fig/σ1=10MPa σ3=8MPa/0026-0004.jpg]

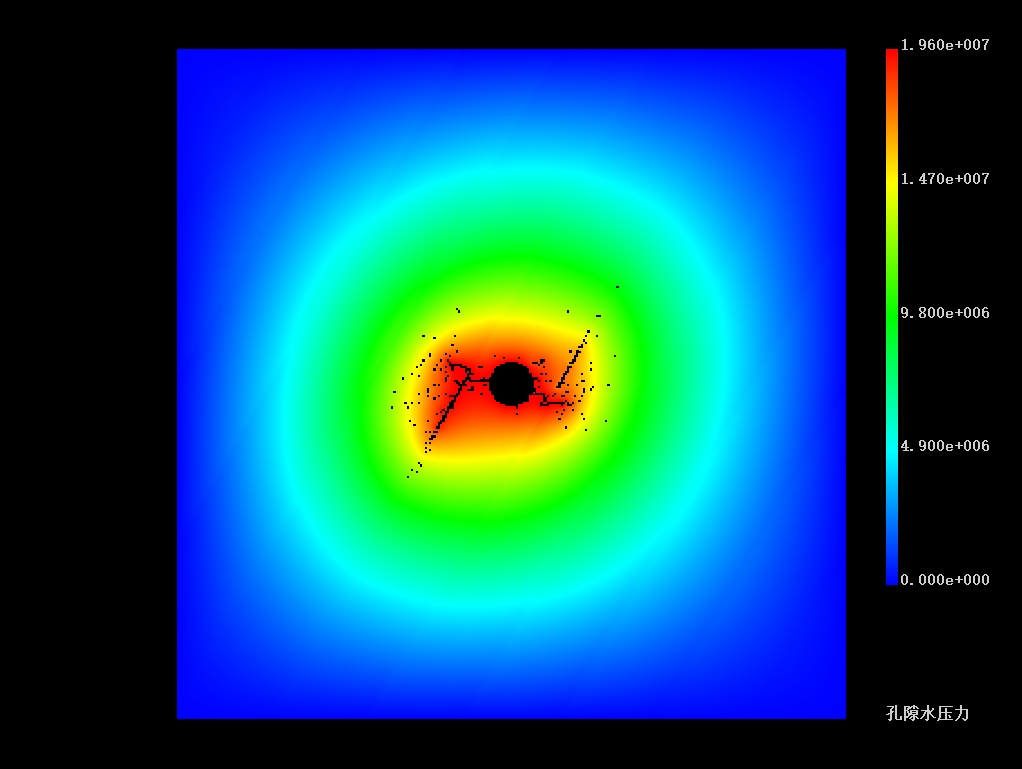

Supplement: S3 Fig — (ZIP) [file pone.0294993.s003.zip › S3_Fig/σ1=10MPa σ3=8MPa/0026-0007.jpg]

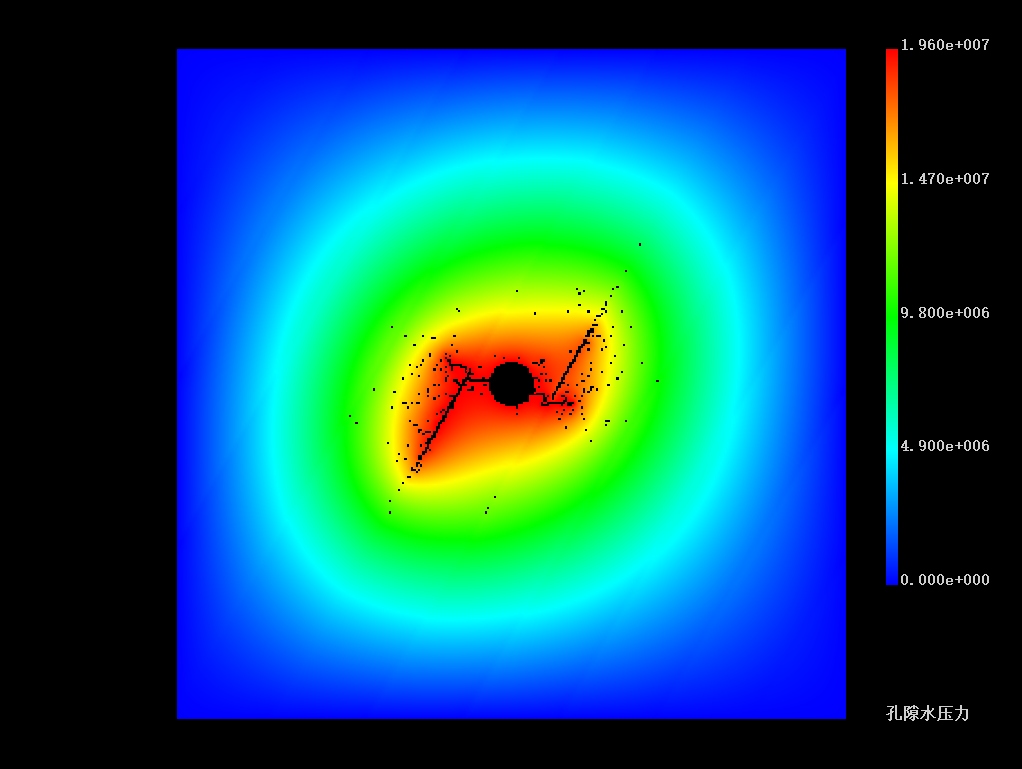

Supplement: S3 Fig — (ZIP) [file pone.0294993.s003.zip › S3_Fig/σ1=10MPa σ3=8MPa/0026-0008.jpg]

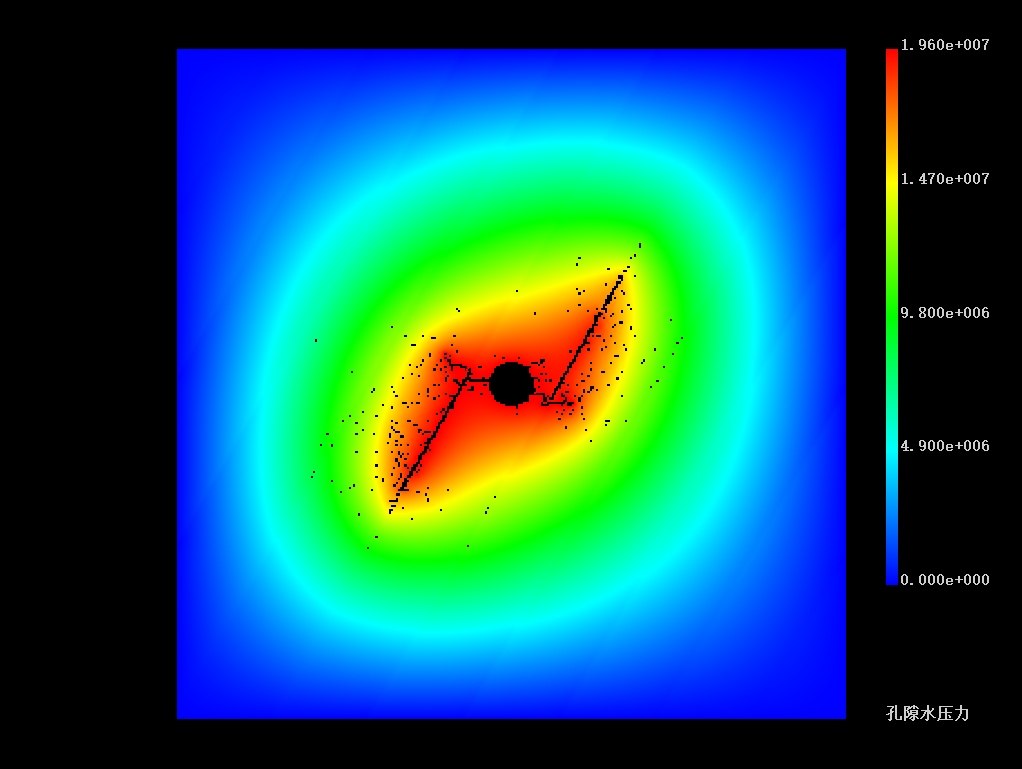

Supplement: S3 Fig — (ZIP) [file pone.0294993.s003.zip › S3_Fig/σ1=10MPa σ3=8MPa/0026-0009.jpg]

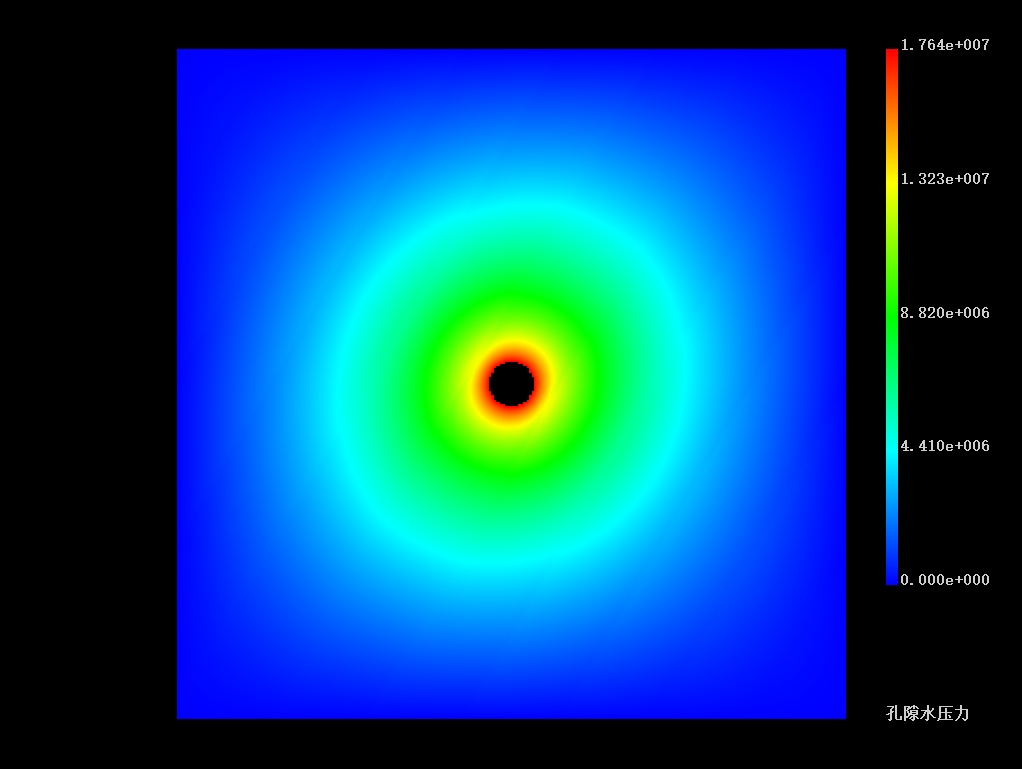

Supplement: S3 Fig — (ZIP) [file pone.0294993.s003.zip › S3_Fig/σ1=12MPa σ3=10MPa/0001-0001.jpg]

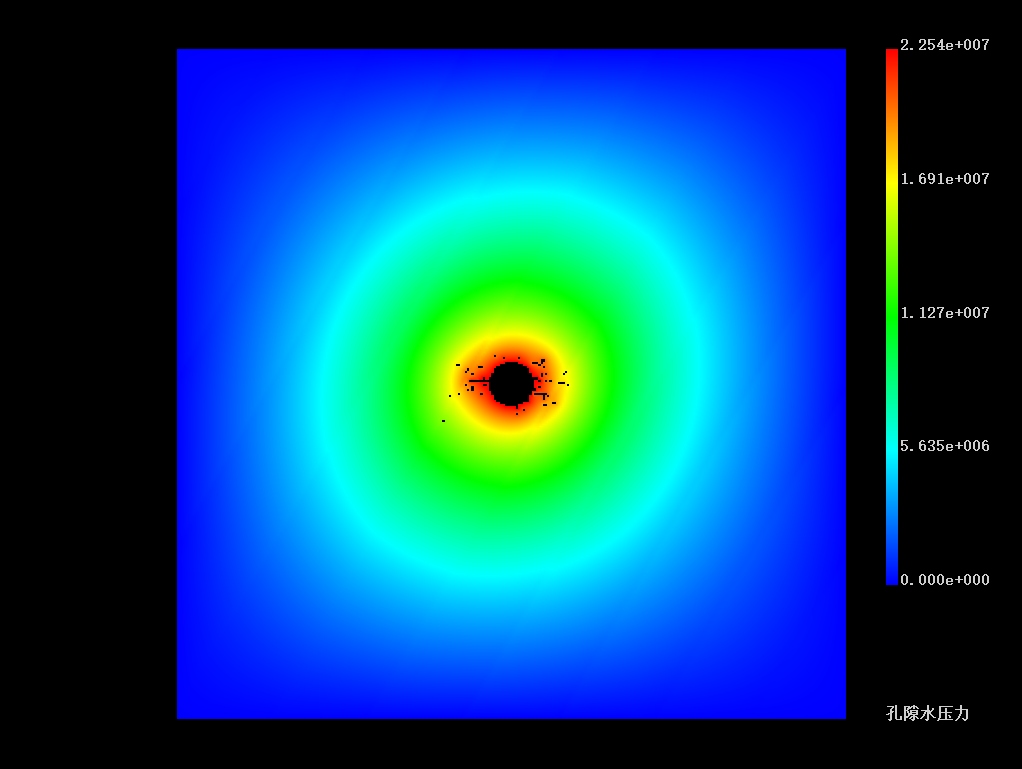

Supplement: S3 Fig — (ZIP) [file pone.0294993.s003.zip › S3_Fig/σ1=12MPa σ3=10MPa/0026-0002.jpg]

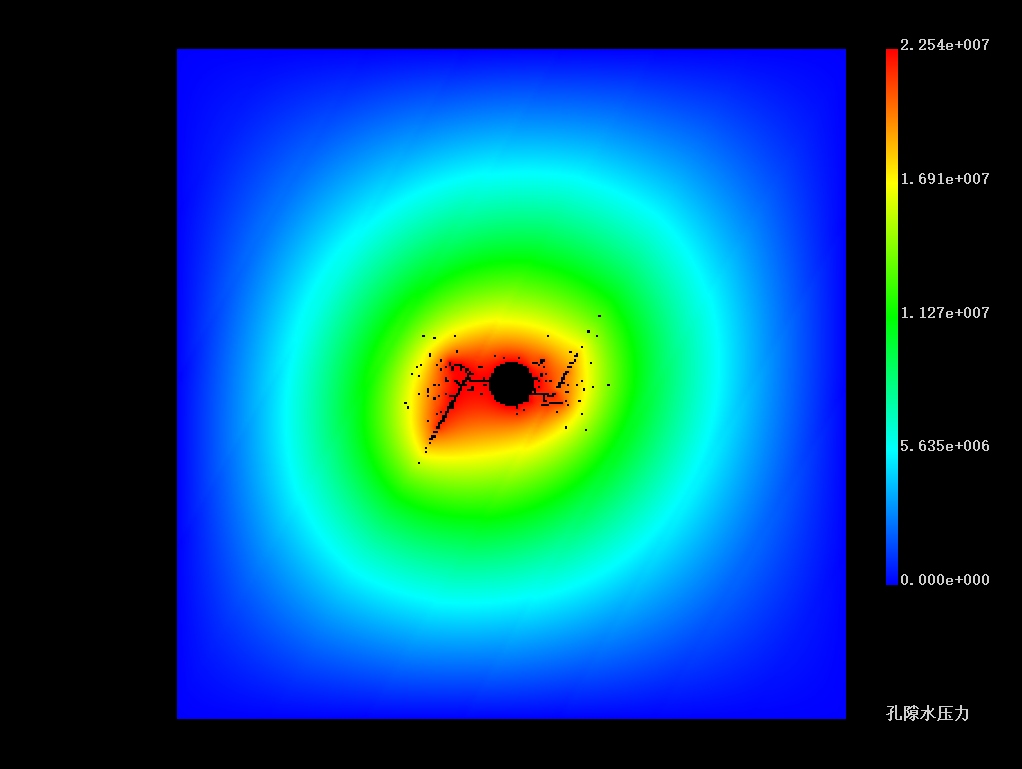

Supplement: S3 Fig — (ZIP) [file pone.0294993.s003.zip › S3_Fig/σ1=12MPa σ3=10MPa/0026-0006.jpg]

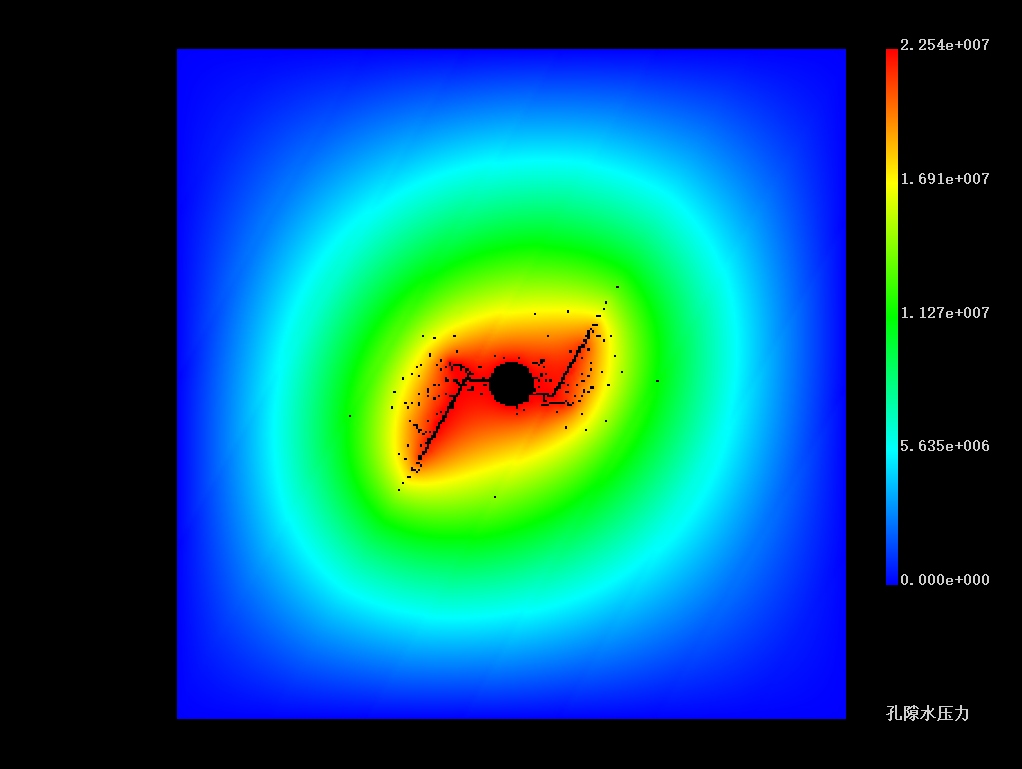

Supplement: S3 Fig — (ZIP) [file pone.0294993.s003.zip › S3_Fig/σ1=12MPa σ3=10MPa/0026-0007.jpg]

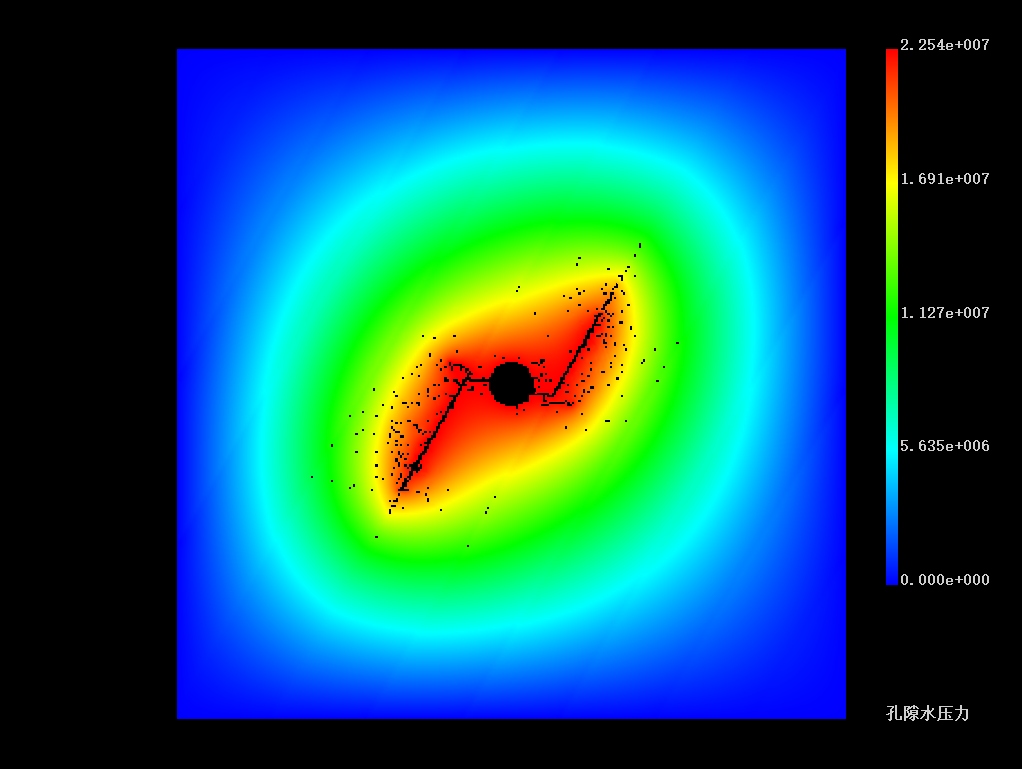

Supplement: S3 Fig — (ZIP) [file pone.0294993.s003.zip › S3_Fig/σ1=12MPa σ3=10MPa/0026-0008.jpg]

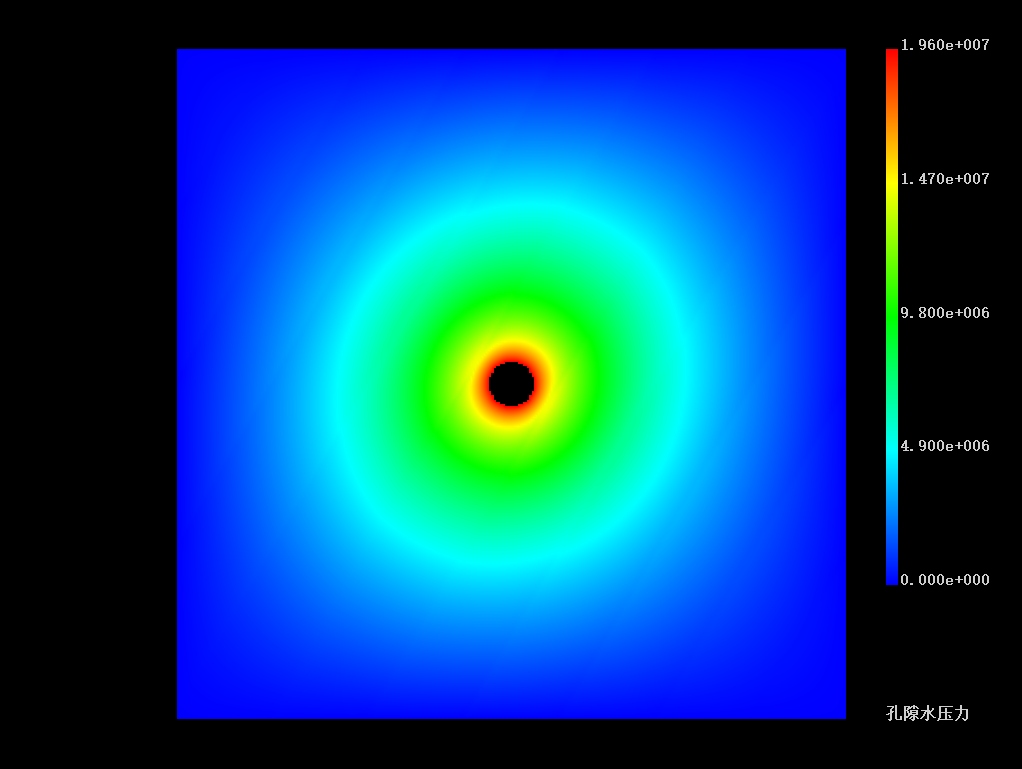

Supplement: S3 Fig — (ZIP) [file pone.0294993.s003.zip › S3_Fig/σ1=14MPa σ3=12MPa/0001-0001.jpg]

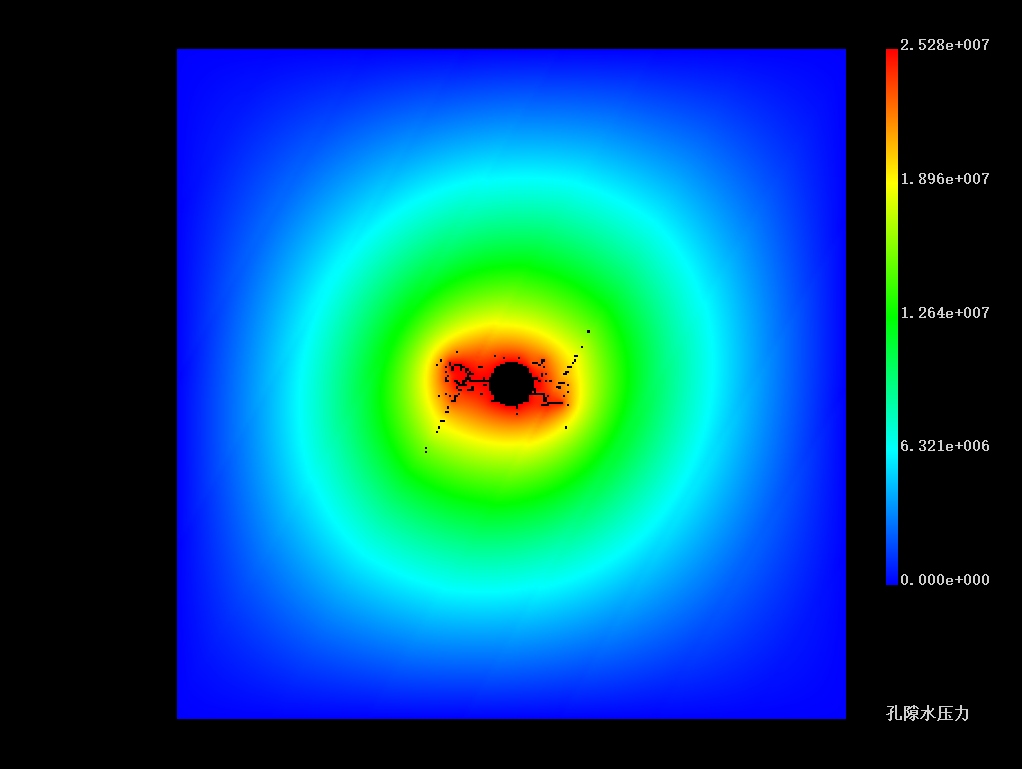

Supplement: S3 Fig — (ZIP) [file pone.0294993.s003.zip › S3_Fig/σ1=14MPa σ3=12MPa/0030-0006.jpg]

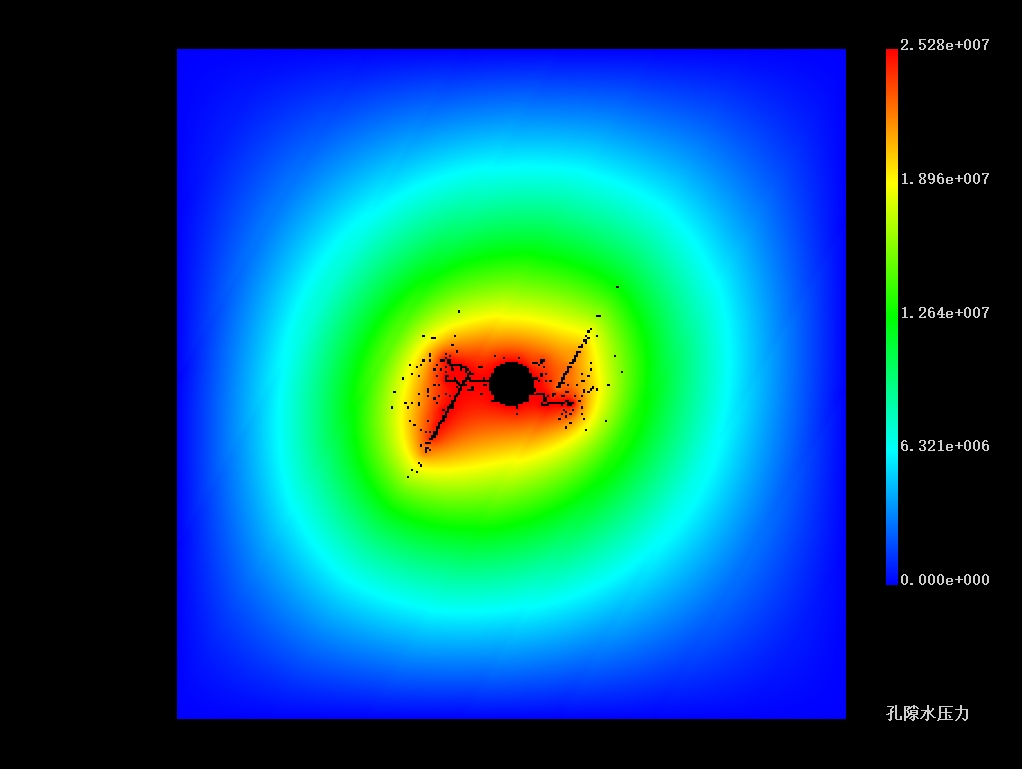

Supplement: S3 Fig — (ZIP) [file pone.0294993.s003.zip › S3_Fig/σ1=14MPa σ3=12MPa/0030-0008.jpg]

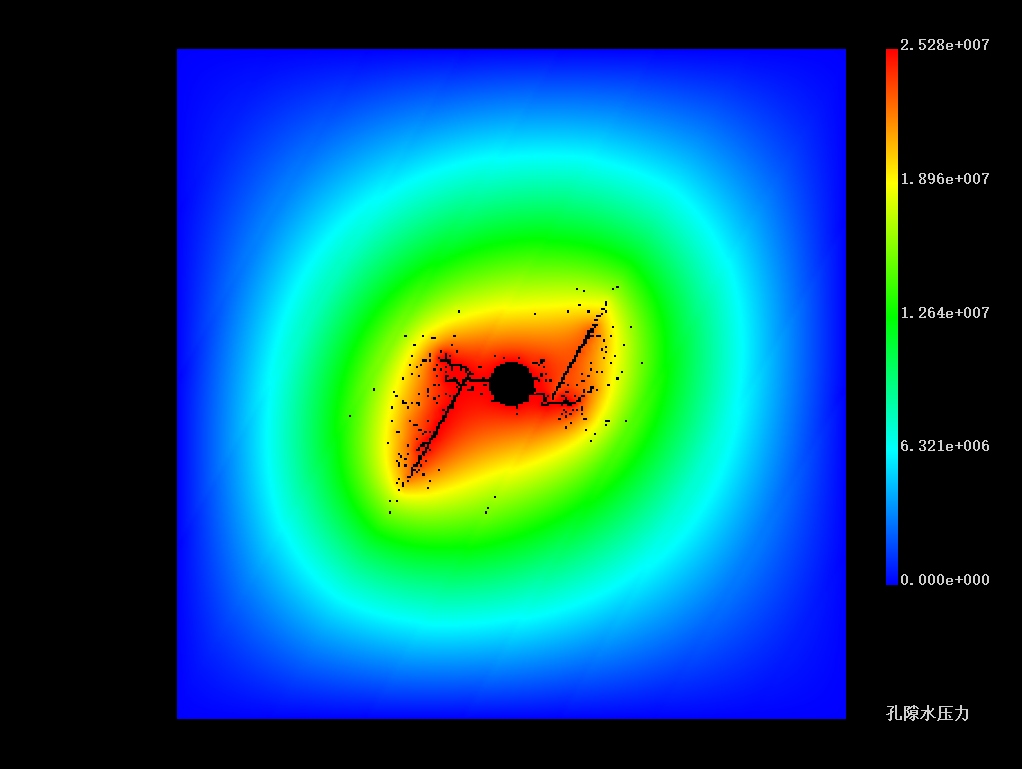

Supplement: S3 Fig — (ZIP) [file pone.0294993.s003.zip › S3_Fig/σ1=14MPa σ3=12MPa/0030-0009.jpg]

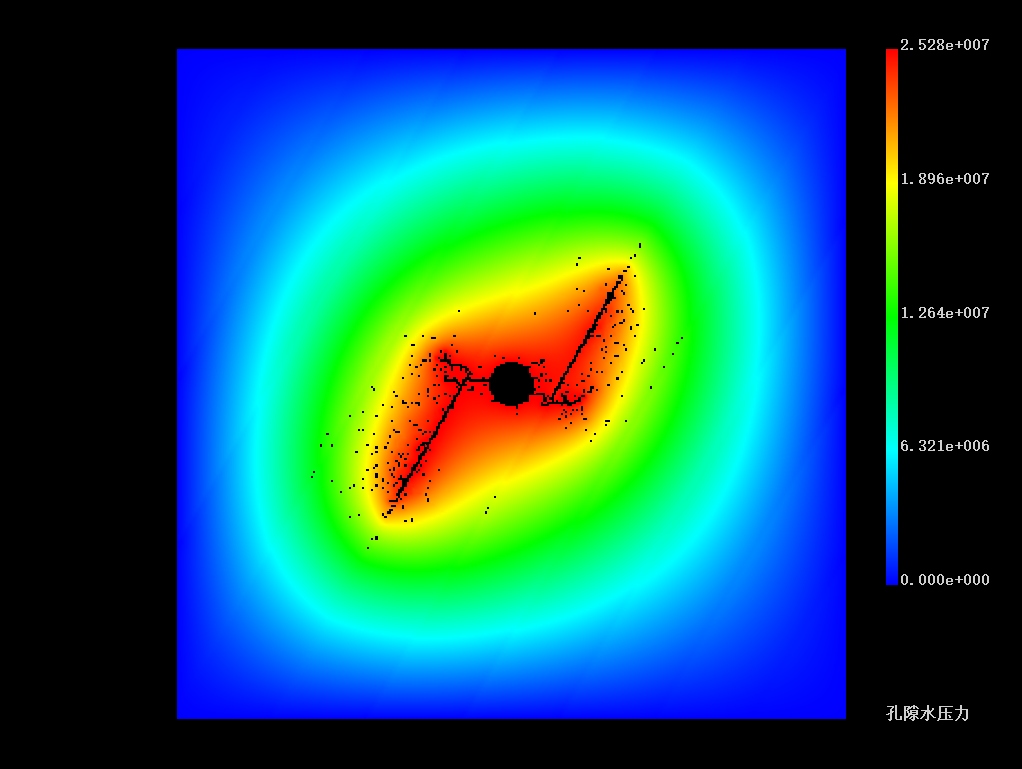

Supplement: S3 Fig — (ZIP) [file pone.0294993.s003.zip › S3_Fig/σ1=14MPa σ3=12MPa/0030-0010.jpg]

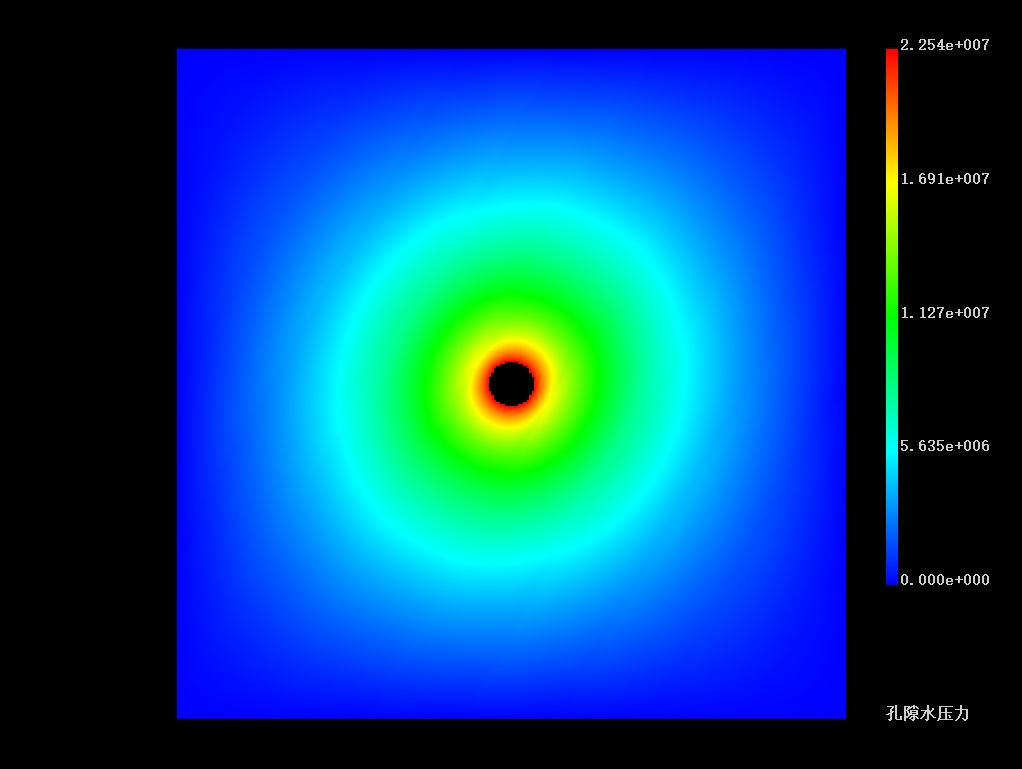

Supplement: S3 Fig — (ZIP) [file pone.0294993.s003.zip › S3_Fig/σ1=16MPa σ3=14MPa/0001-0001.jpg]

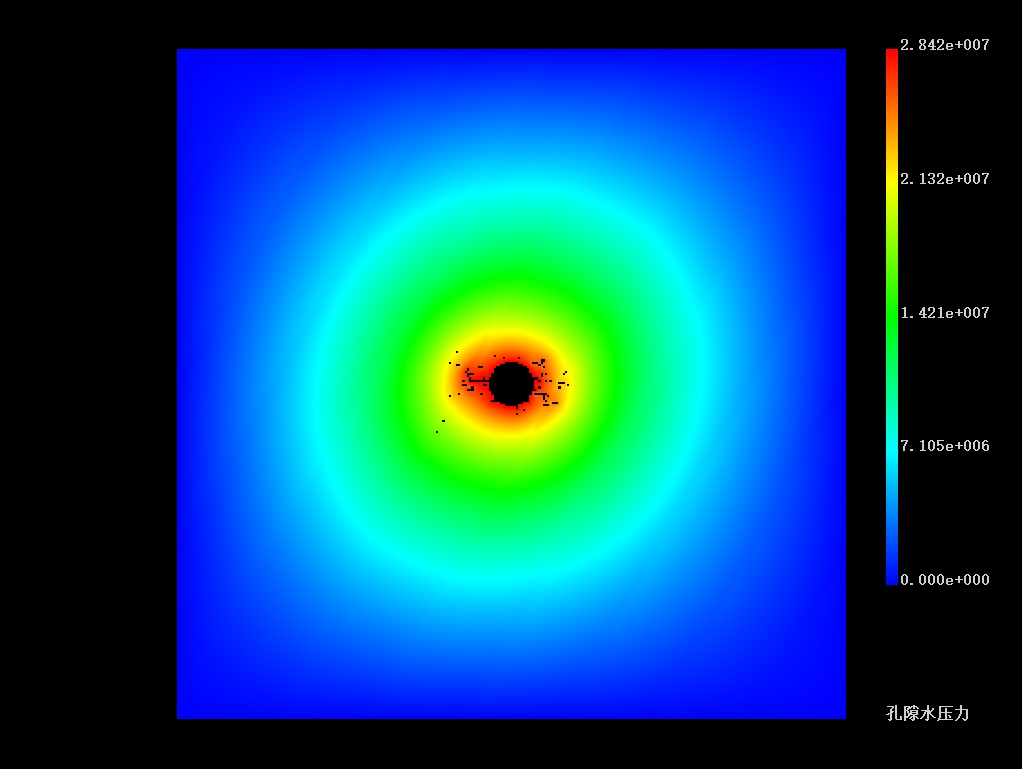

Supplement: S3 Fig — (ZIP) [file pone.0294993.s003.zip › S3_Fig/σ1=16MPa σ3=14MPa/0031-0003.jpg]

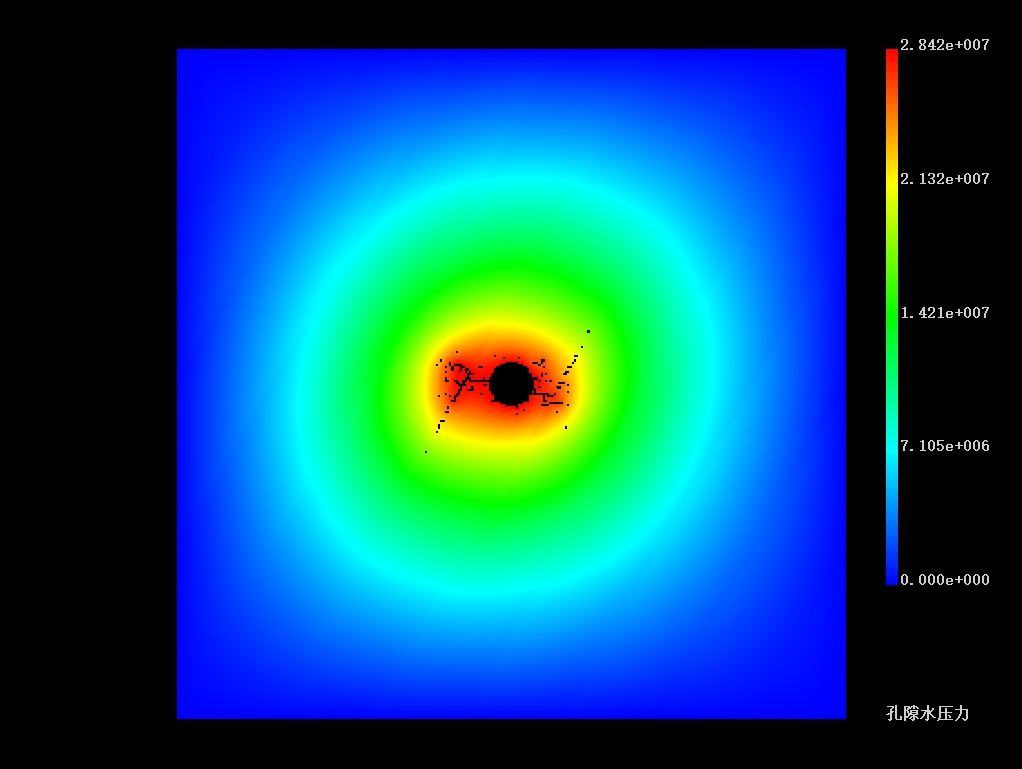

Supplement: S3 Fig — (ZIP) [file pone.0294993.s003.zip › S3_Fig/σ1=16MPa σ3=14MPa/0031-0005.jpg]

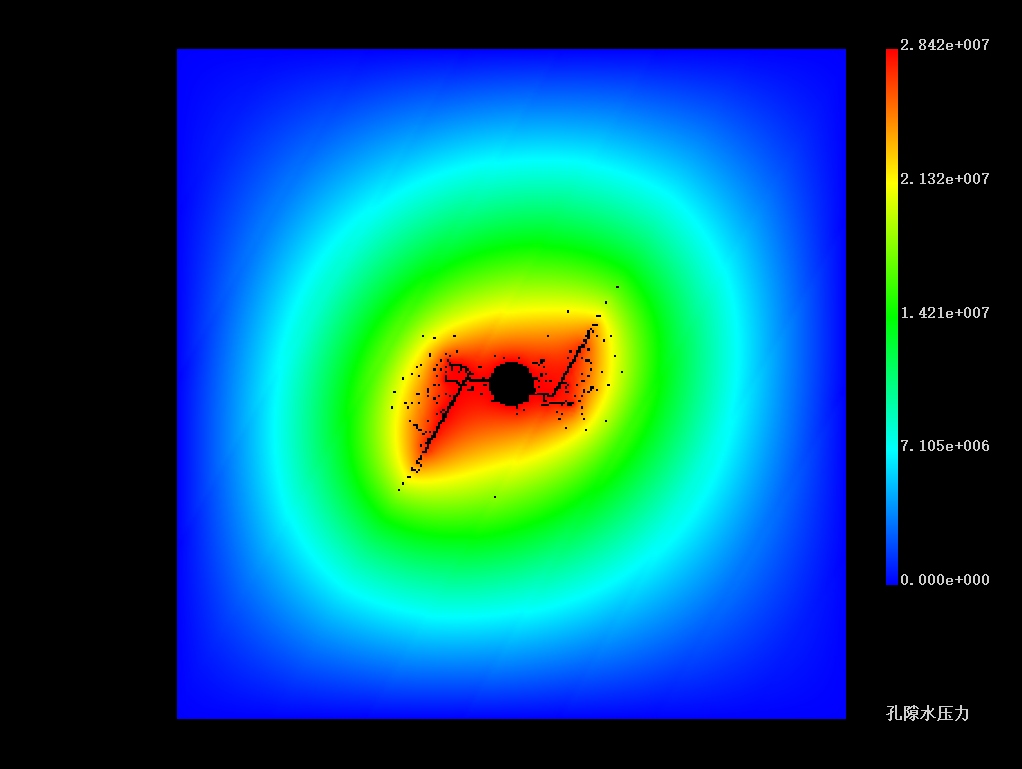

Supplement: S3 Fig — (ZIP) [file pone.0294993.s003.zip › S3_Fig/σ1=16MPa σ3=14MPa/0031-0007.jpg]

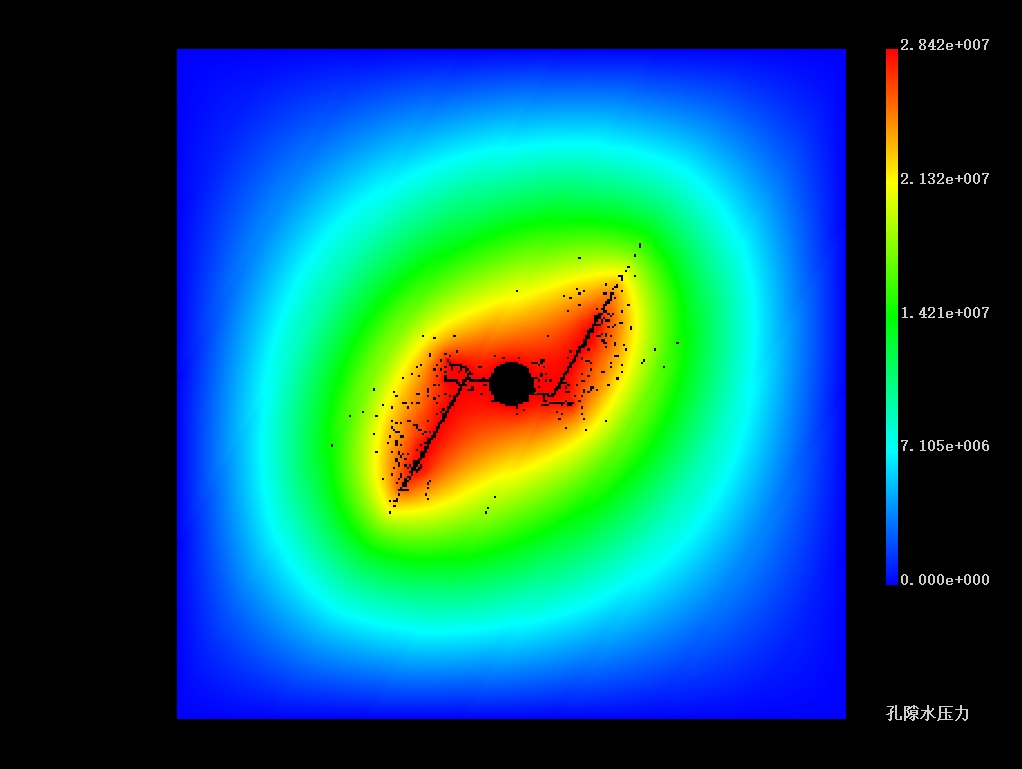

Supplement: S3 Fig — (ZIP) [file pone.0294993.s003.zip › S3_Fig/σ1=16MPa σ3=14MPa/0031-0008.jpg]

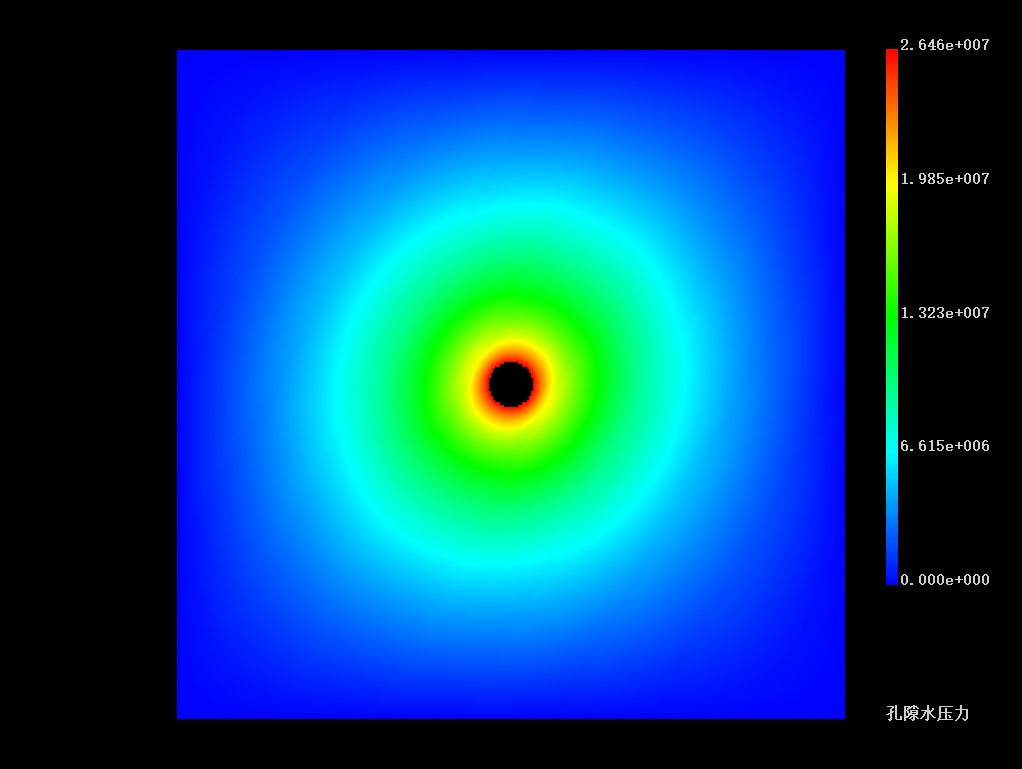

Supplement: S3 Fig — (ZIP) [file pone.0294993.s003.zip › S3_Fig/σ1=18MPa σ3=16MPa/0001-0001.jpg]

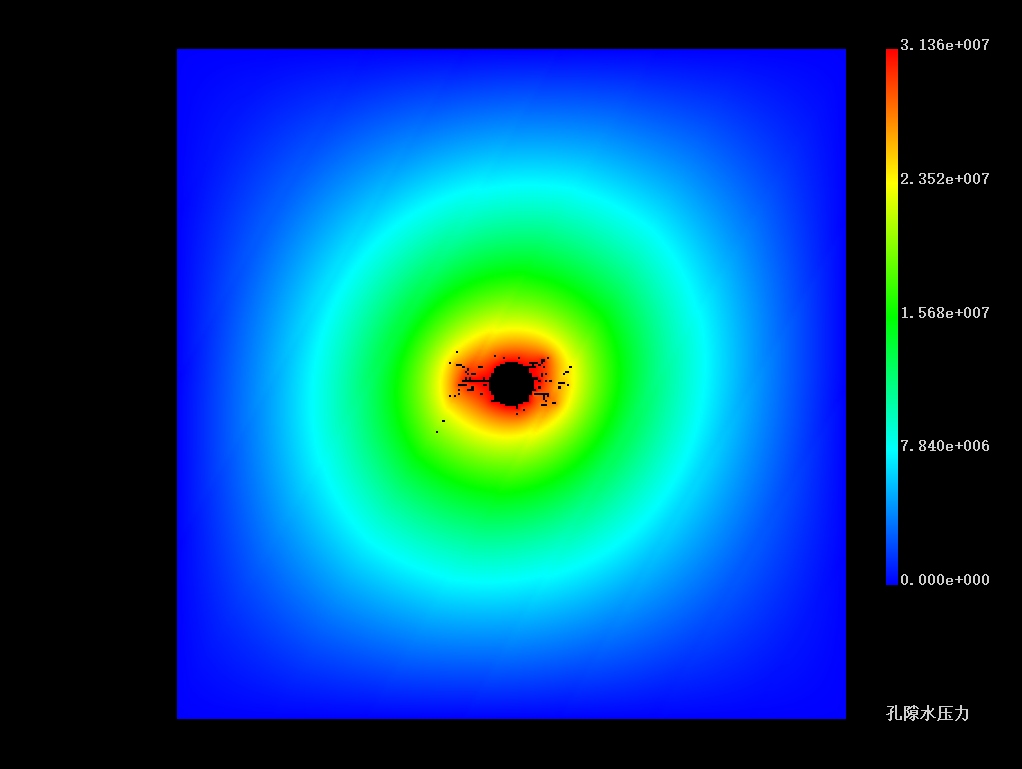

Supplement: S3 Fig — (ZIP) [file pone.0294993.s003.zip › S3_Fig/σ1=18MPa σ3=16MPa/0026-0003.jpg]

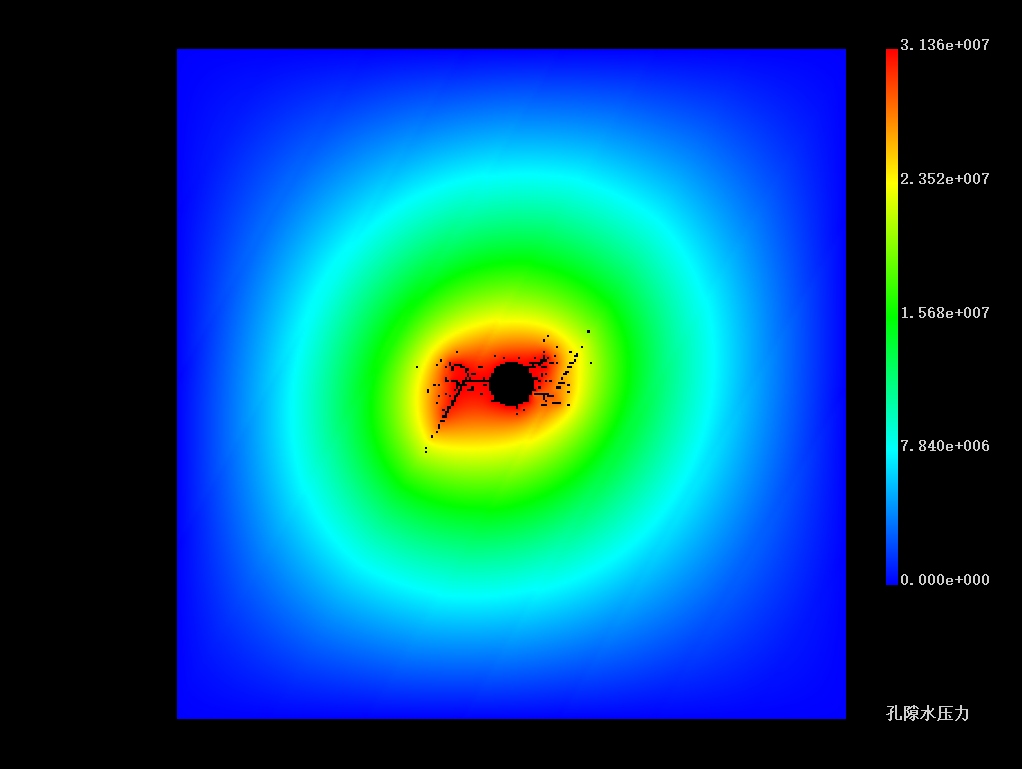

Supplement: S3 Fig — (ZIP) [file pone.0294993.s003.zip › S3_Fig/σ1=18MPa σ3=16MPa/0026-0005.jpg]

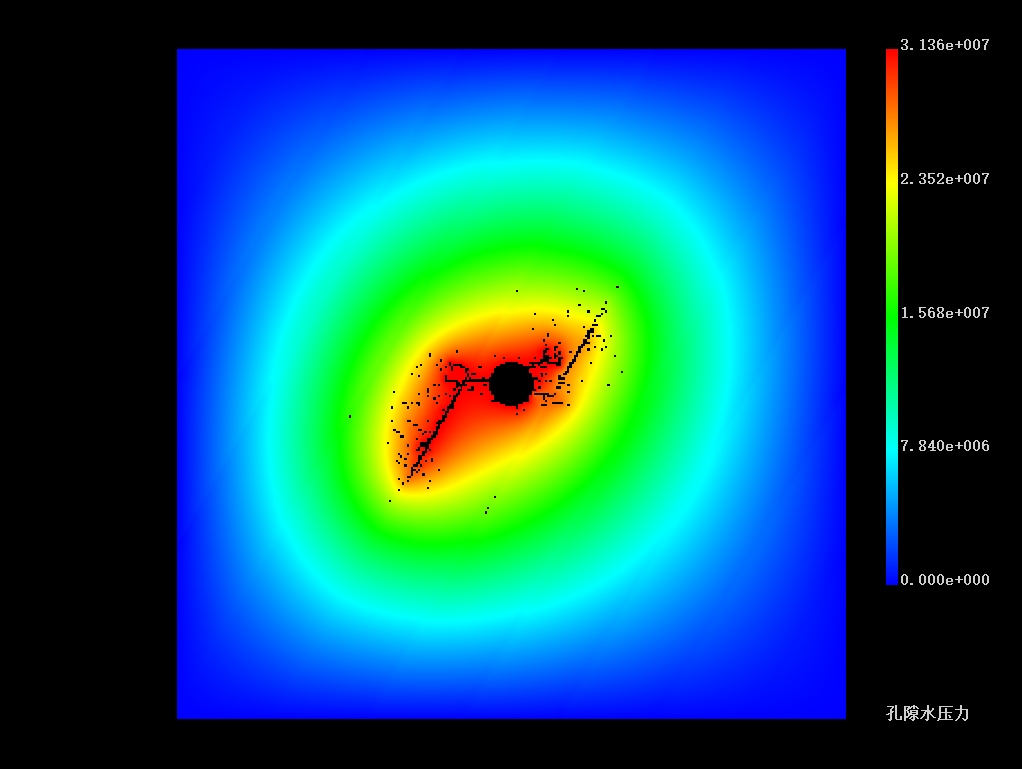

Supplement: S3 Fig — (ZIP) [file pone.0294993.s003.zip › S3_Fig/σ1=18MPa σ3=16MPa/0026-0007.jpg]

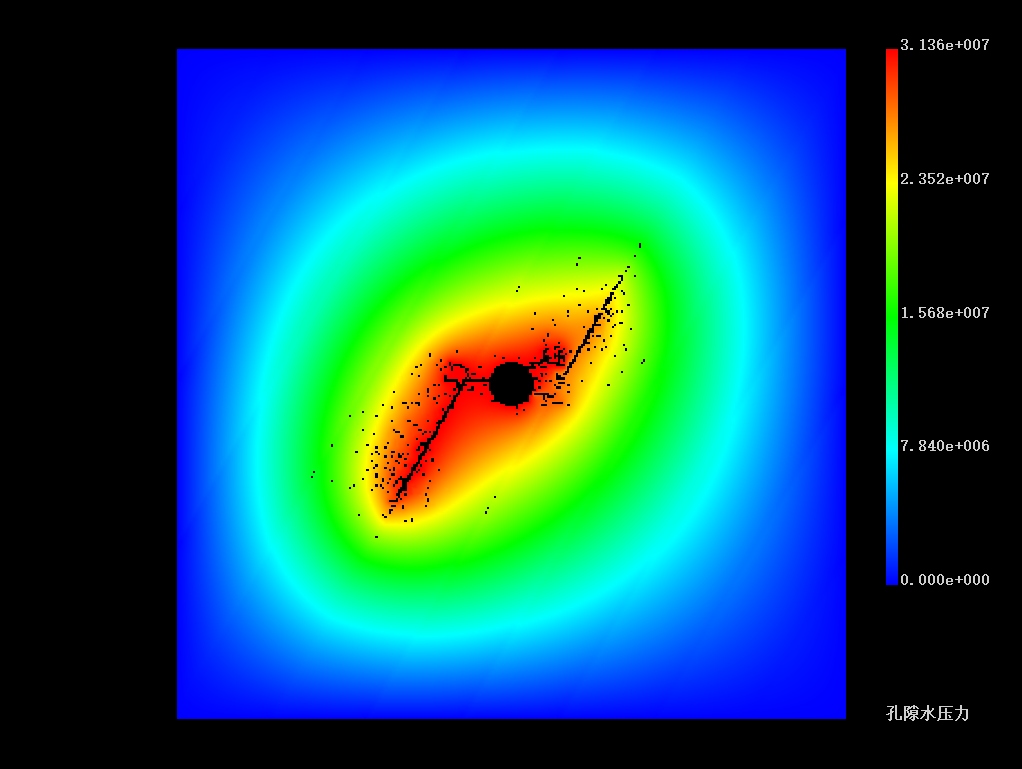

Supplement: S3 Fig — (ZIP) [file pone.0294993.s003.zip › S3_Fig/σ1=18MPa σ3=16MPa/0026-0008.jpg]

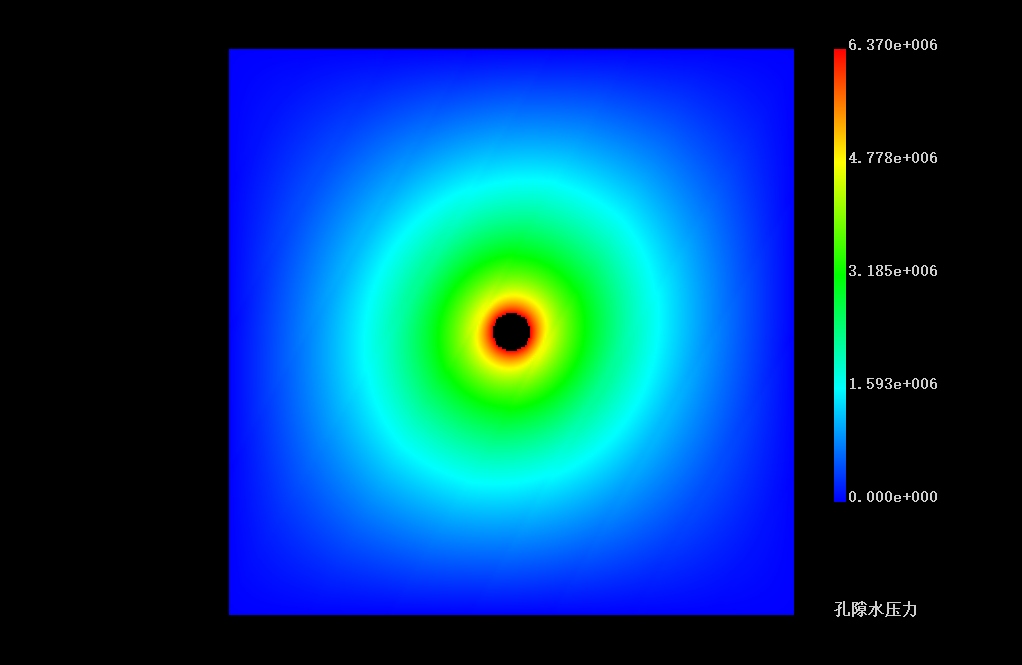

Supplement: S3 Fig — (ZIP) [file pone.0294993.s003.zip › S3_Fig/σ1=4MPa σ3=2MPa/0001-0001.jpg]

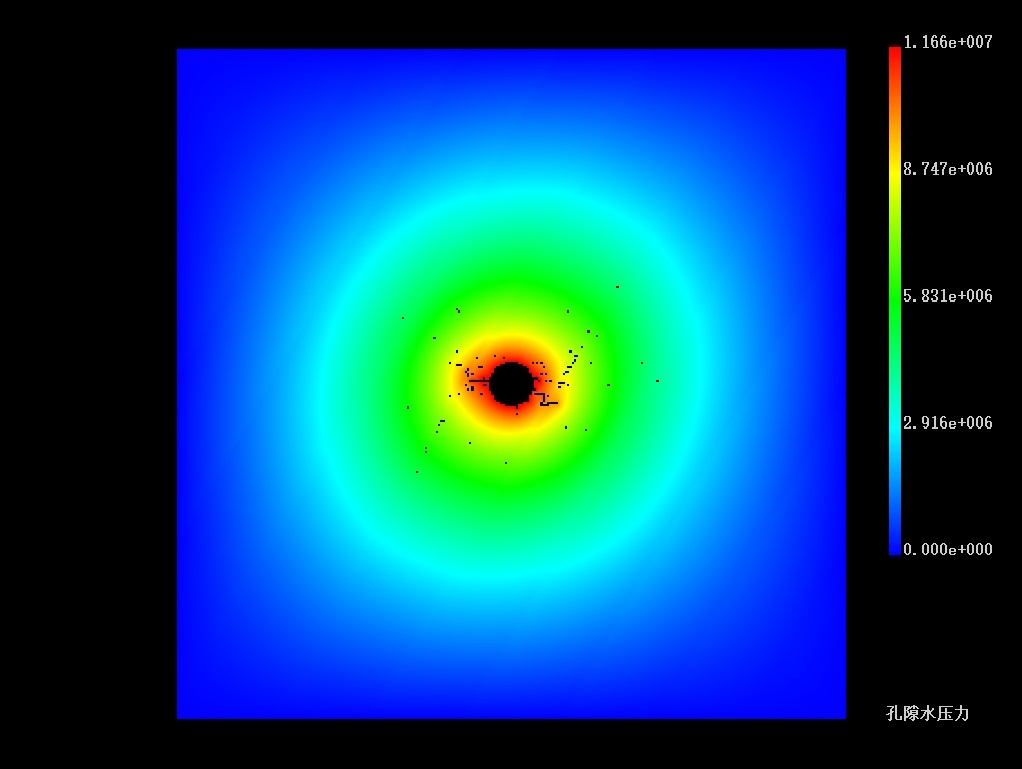

Supplement: S3 Fig — (ZIP) [file pone.0294993.s003.zip › S3_Fig/σ1=4MPa σ3=2MPa/0055-0003.jpg]

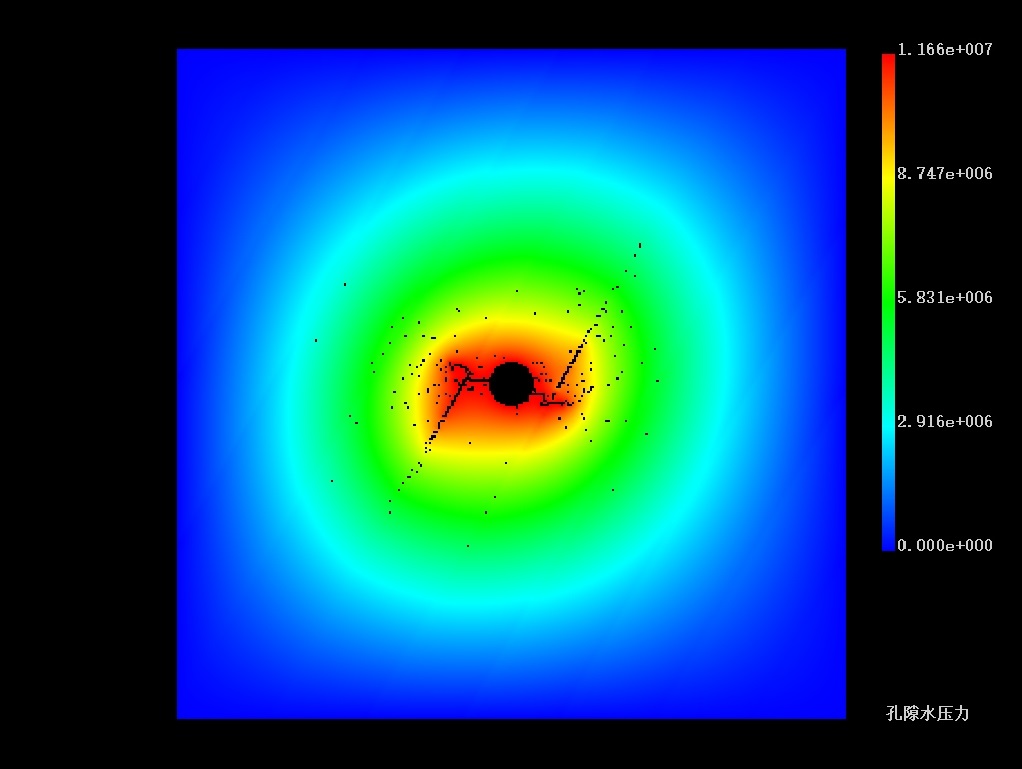

Supplement: S3 Fig — (ZIP) [file pone.0294993.s003.zip › S3_Fig/σ1=4MPa σ3=2MPa/0055-0006.jpg]

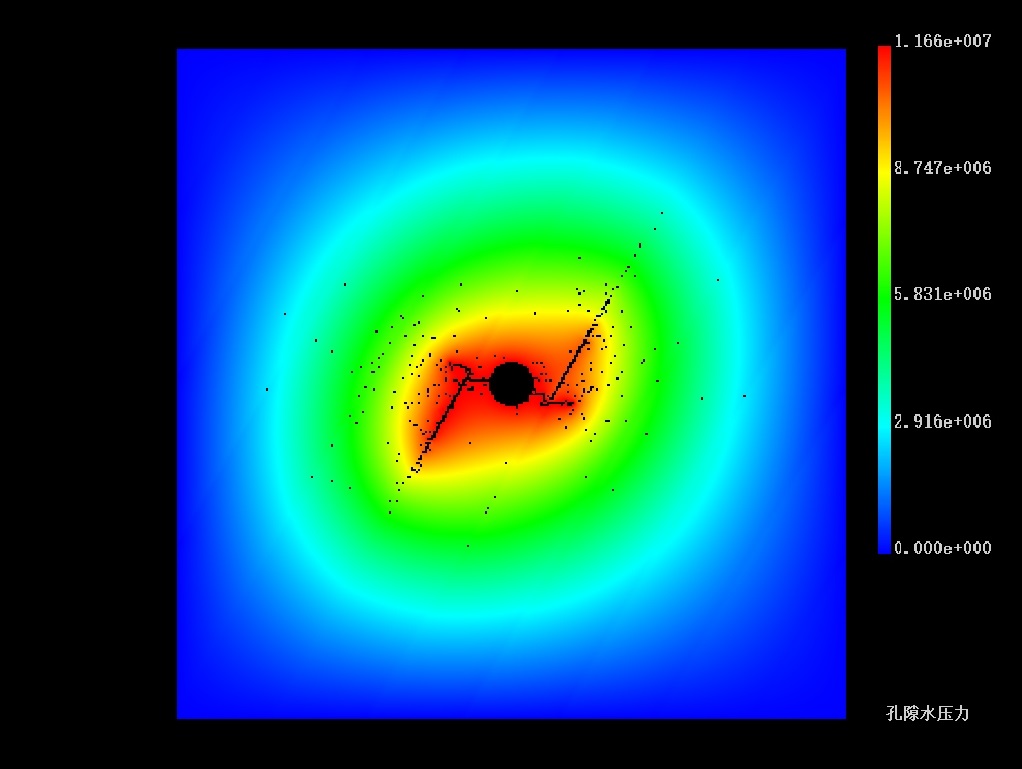

Supplement: S3 Fig — (ZIP) [file pone.0294993.s003.zip › S3_Fig/σ1=4MPa σ3=2MPa/0055-0007.jpg]

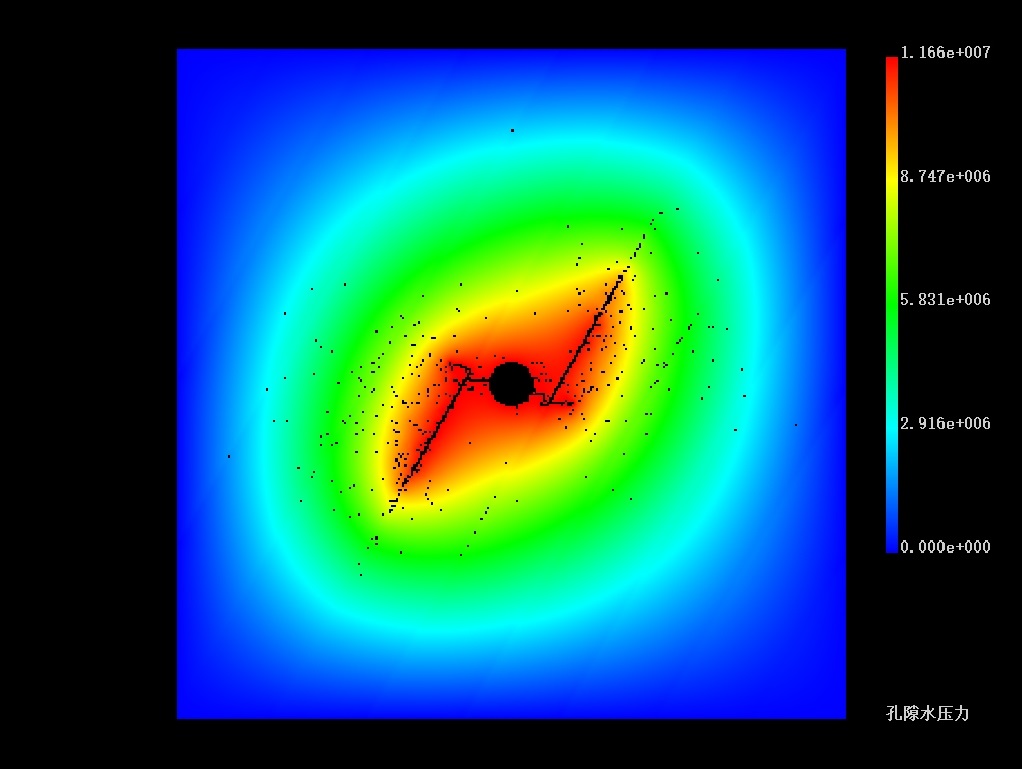

Supplement: S3 Fig — (ZIP) [file pone.0294993.s003.zip › S3_Fig/σ1=4MPa σ3=2MPa/0055-0008.jpg]

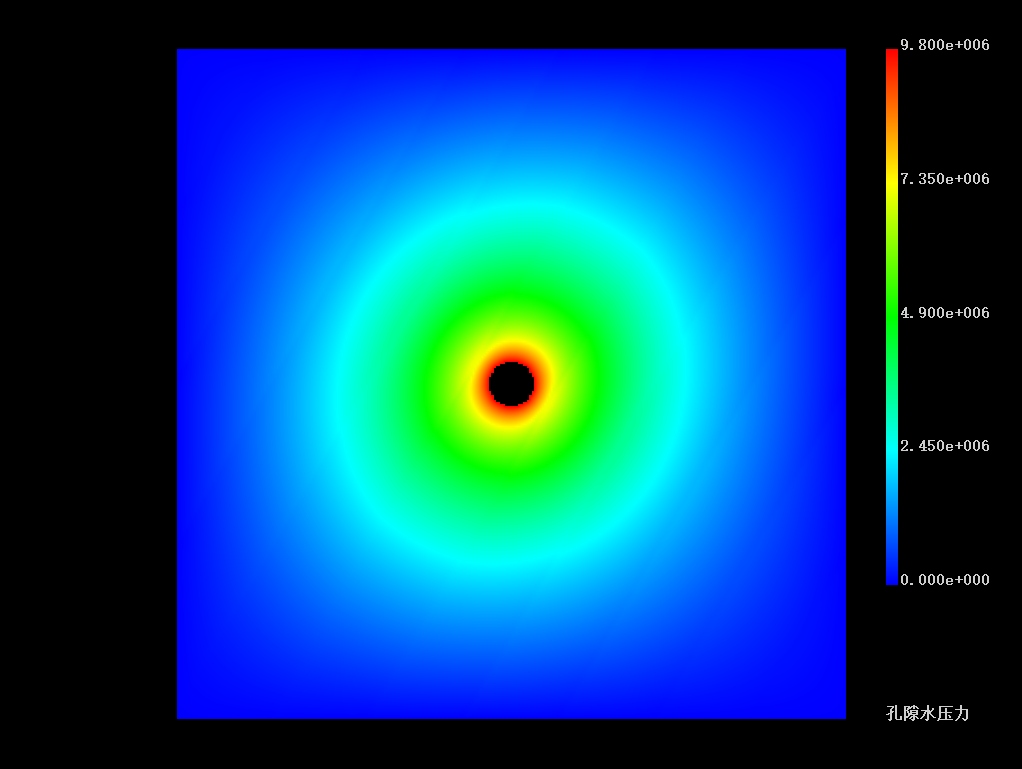

Supplement: S3 Fig — (ZIP) [file pone.0294993.s003.zip › S3_Fig/σ1=6MPa σ3=4MPa/0001-0001.jpg]

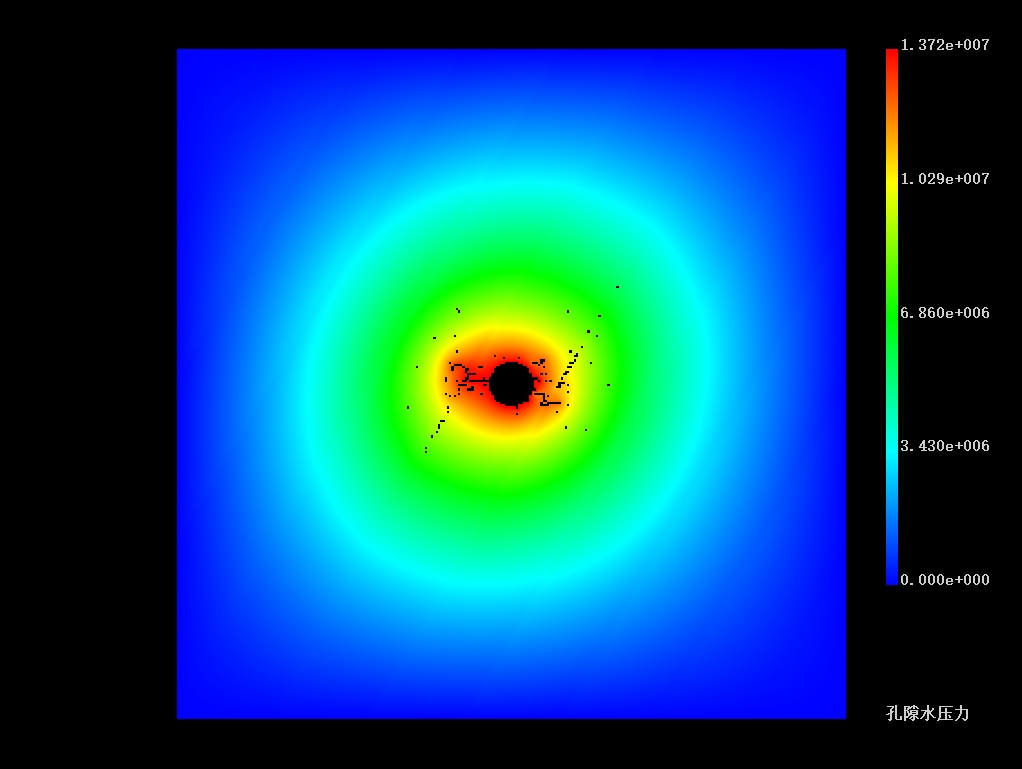

Supplement: S3 Fig — (ZIP) [file pone.0294993.s003.zip › S3_Fig/σ1=6MPa σ3=4MPa/0021-0004.jpg]

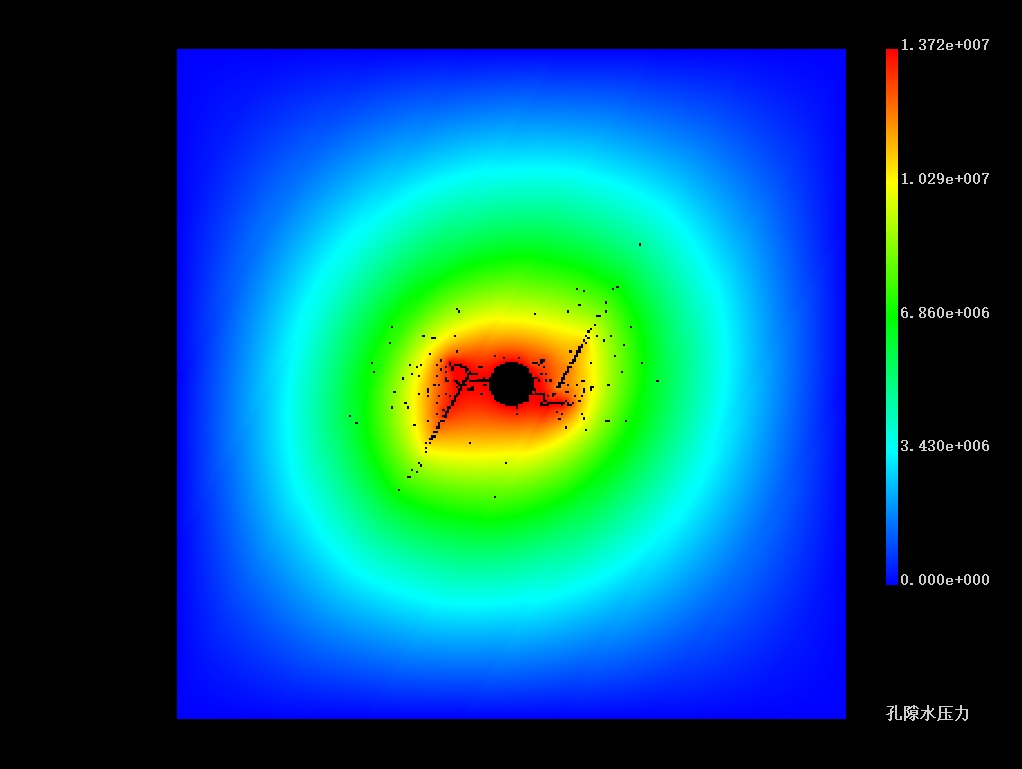

Supplement: S3 Fig — (ZIP) [file pone.0294993.s003.zip › S3_Fig/σ1=6MPa σ3=4MPa/0021-0006.jpg]

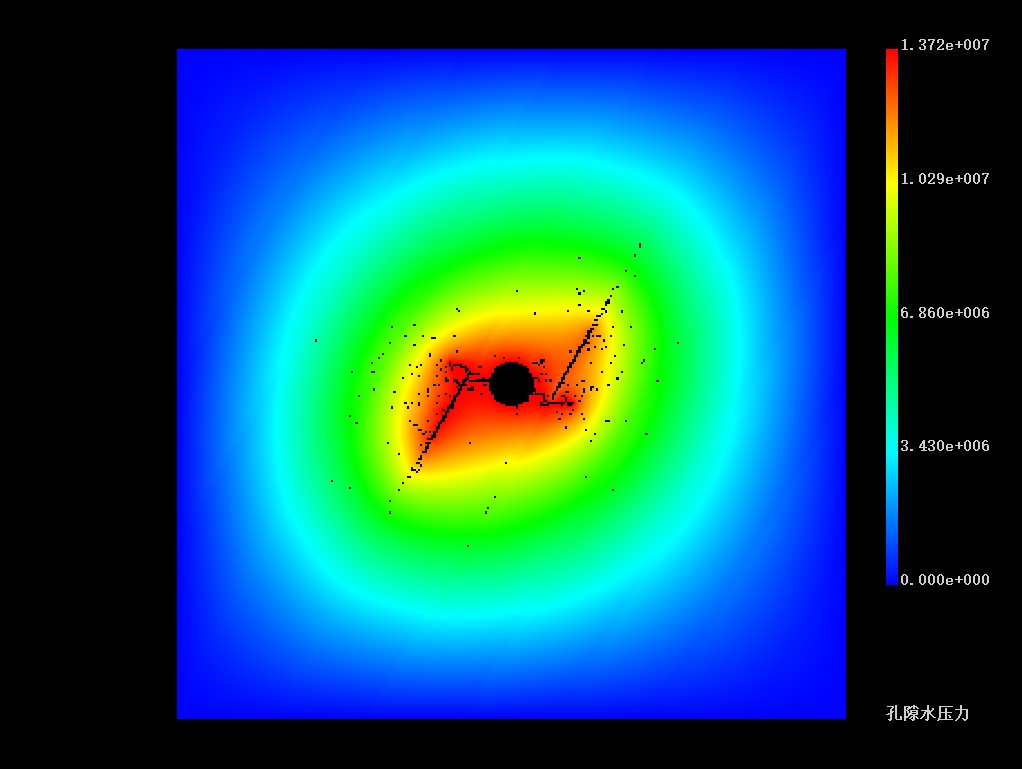

Supplement: S3 Fig — (ZIP) [file pone.0294993.s003.zip › S3_Fig/σ1=6MPa σ3=4MPa/0021-0007.jpg]

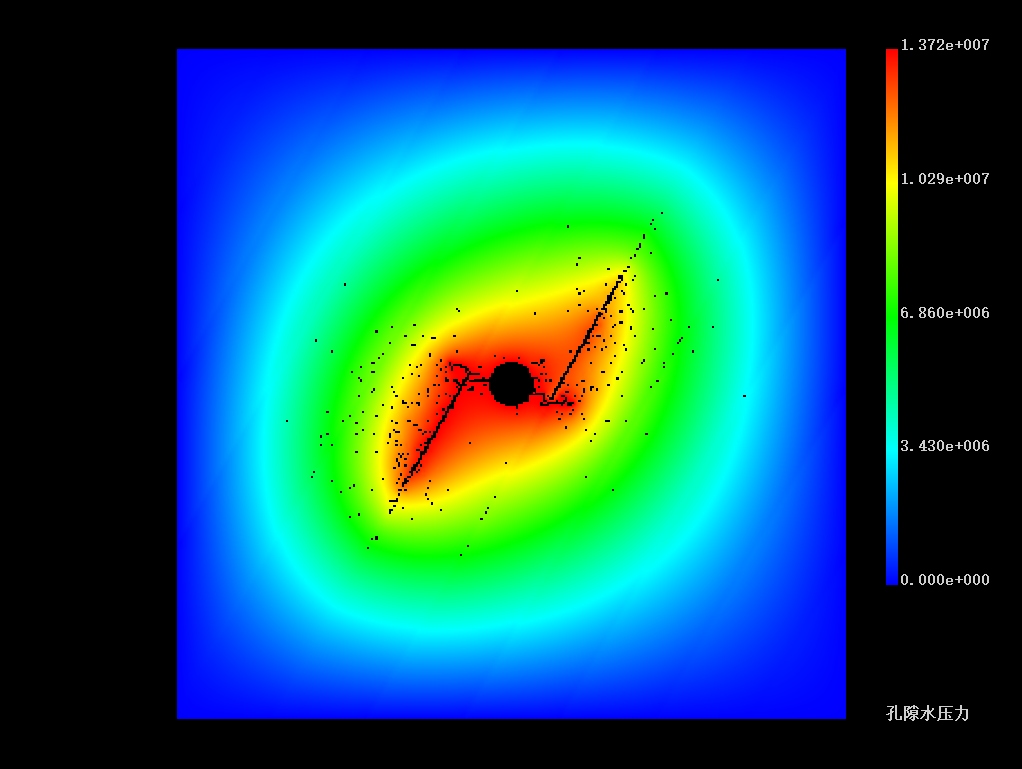

Supplement: S3 Fig — (ZIP) [file pone.0294993.s003.zip › S3_Fig/σ1=6MPa σ3=4MPa/0021-0008.jpg]

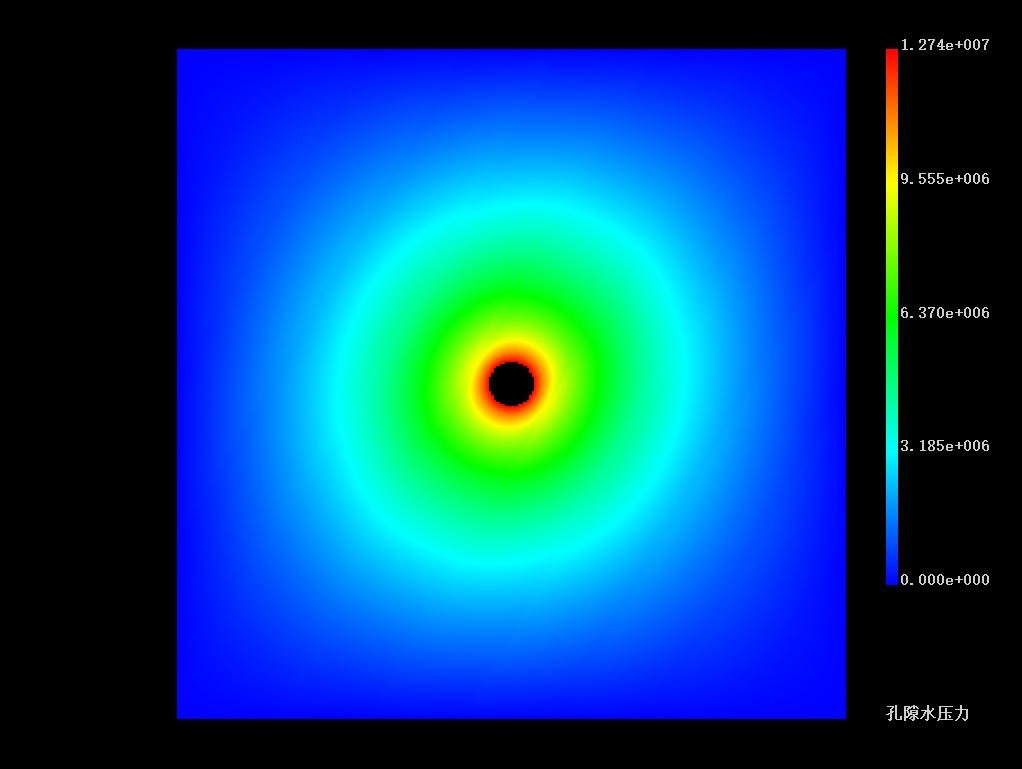

Supplement: S3 Fig — (ZIP) [file pone.0294993.s003.zip › S3_Fig/σ1=8MPa σ3=6MPa/0001-0001.jpg]

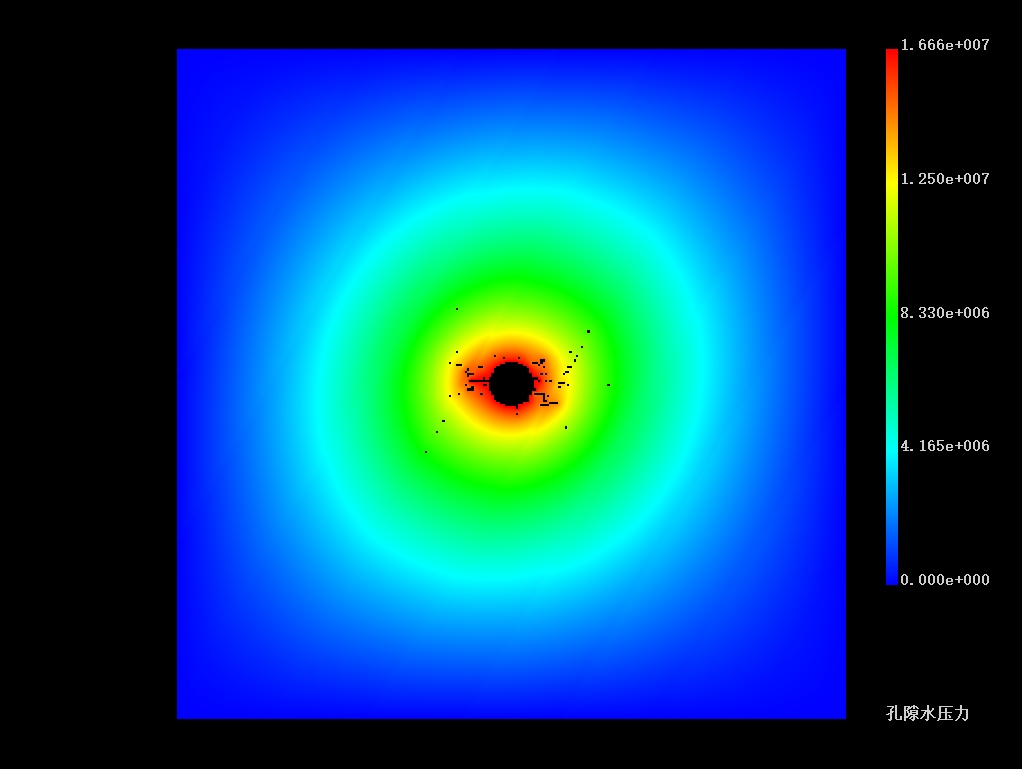

Supplement: S3 Fig — (ZIP) [file pone.0294993.s003.zip › S3_Fig/σ1=8MPa σ3=6MPa/0021-0004.jpg]

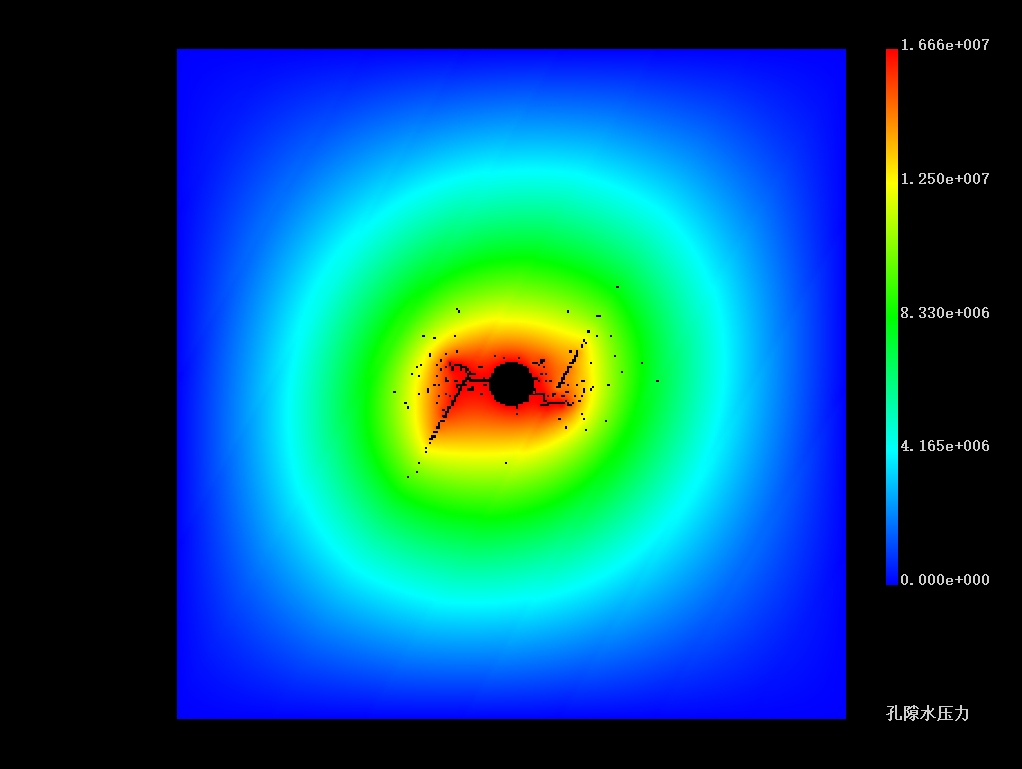

Supplement: S3 Fig — (ZIP) [file pone.0294993.s003.zip › S3_Fig/σ1=8MPa σ3=6MPa/0021-0007.jpg]

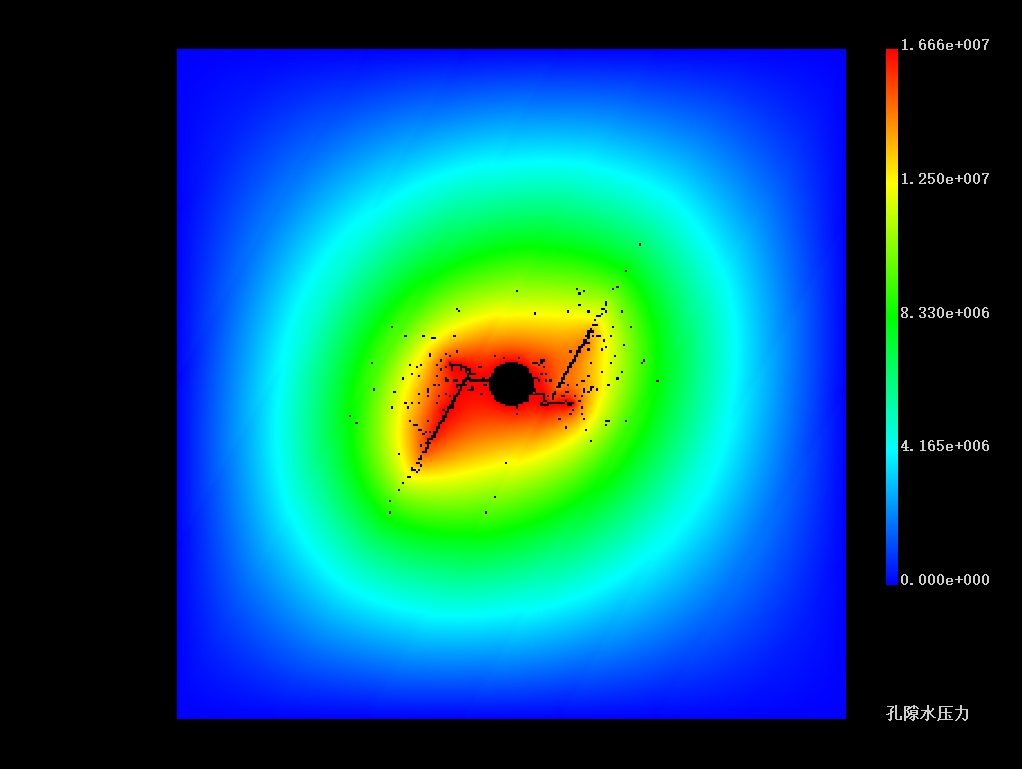

Supplement: S3 Fig — (ZIP) [file pone.0294993.s003.zip › S3_Fig/σ1=8MPa σ3=6MPa/0021-0008.jpg]

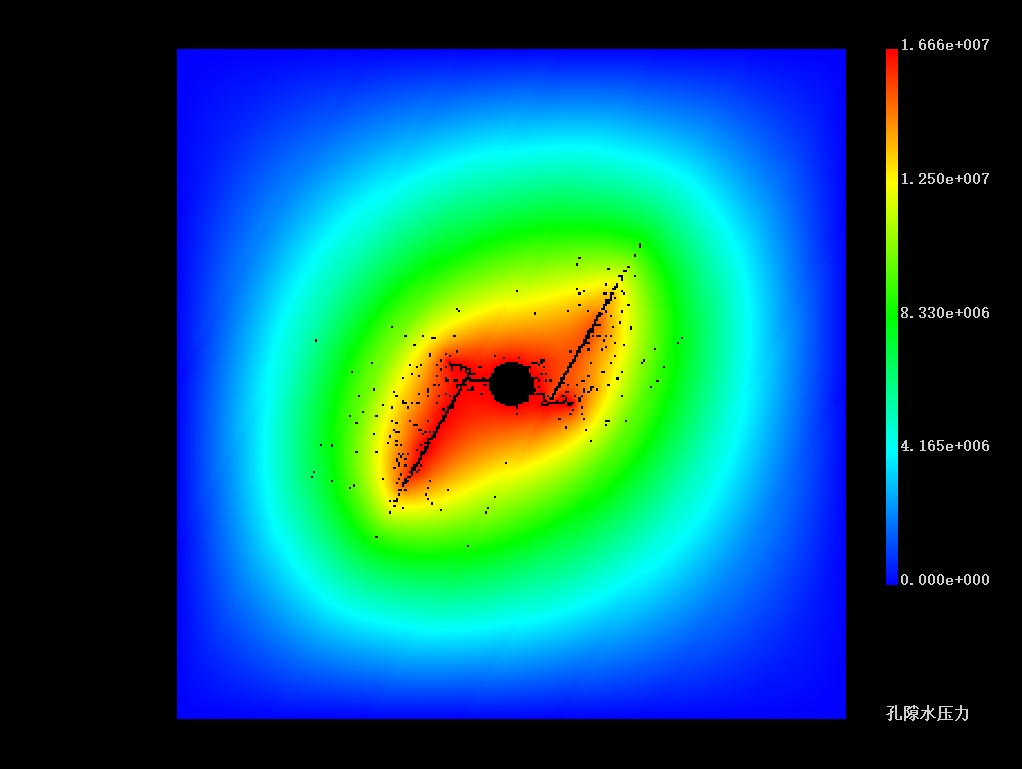

Supplement: S3 Fig — (ZIP) [file pone.0294993.s003.zip › S3_Fig/σ1=8MPa σ3=6MPa/0021-0009.jpg]
